# Current status, challenges and control of human sparganosis worldwide: a scoping review

**DOI:** 10.1186/s40249-026-01434-9

**Published:** 2026-03-31

**Authors:** Mi Li, Sisi Ru, Jingjing Xu, Jiahui Liang, Xi Zhang, Shengxia Chen

**Affiliations:** 1 Department of Laboratory Medicine, School of Medicine, Jiangsu University, Zhenjiang, Jiangsu China; 2 Department of Parasitology, School of Basic Medical Sciences, Zhengzhou University, Zhengzhou, Henan China

**Keywords:** Human sparganosis, *Spirometra* spp., Sparganum, Epidemiology, Global distribution

## Abstract

**Background:**

Human sparganosis is a neglected foodborne/waterborne zoonosis and a rare infectious disease of poverty (rIDP) that is widely distributed globally and threatens human health. Economic development and the expanded dissemination of public health knowledge have fueled increased global focus on rIDPs. The epidemiology of human sparganosis has shifted on the basis of emerging evidence; moreover, comprehensive contemporary data on its global distribution remain outdated. To address this gap, we conducted a systematic retrospective analysis to map the global case distribution of human sparganosis. We then summarized and analyzed the basic demographic characteristics, infection sites, modes of transmission and diagnostic methods.

**Methods:**

We systematically searched the China National Knowledge Infrastructure, Wanfang, PubMed, Web of Science, Scopus and Embase databases for case reports, dissertations, reviews and meeting abstracts. The literature search was conducted up to April 30, 2025, without any restrictions on the year of publication. The analysis focused exclusively on articles on human sparganosis cases, with no restrictions on publication language or country of origin. We followed the inclusion and exclusion criteria to identify relevant studies. The relevant information from the identified studies was subsequently collected and summarized.

**Results:**

A total of 822 articles involving 3472 valid cases and 16 serological survey articles involving 3148 positive subjects were identified and included. Our analysis revealed the global distribution of human sparganosis. The disease is distributed worldwide, while China, the Republic of Korea and Thailand are the top three countries in terms of reported case concentration. However, some African regions, such as South Sudan, Tanzania and Ethiopia, may have the potential for a greater number of human sparganosis cases.

**Conclusions:**

This scoping review provides updates on the global distribution changes and epidemiological status of human sparganosis. East Asia and Southeast Asia continue to be regions where human sparganosis is highly prevalent, while the disease burden in some resource-limited regions may be underestimated. Our findings may receive increasing attention in endemic regions, raise awareness in resource-limited, nonendemic areas. In the future, there is still a need to strengthen the prevention and control of this disease worldwide.

**Supplementary Information:**

The online version contains supplementary material available at 10.1186/s40249-026-01434-9.

## Background

Sparganosis, a disease caused by the plerocercoid larva of diphyllobothroid tapeworms belonging to the genus *Spirometra*, is not only one of the rare infectious diseases of poverty (rIDPs) [1–3] but also a neglected foodborne/waterborne zoonosis [4]. Since a human plerocercoid was first described by Manson in 1882 (Xiamen, China) and later identified and named *Ligula mansoni* by Cobbold, this parasite has been found to have a global distribution, with a high prevalence in Asia [5, 6]. More than 60 species of *Spirometra* have been reported worldwide, but only four of these species are considered valid (*S*. *erinaceieuropaei*,* S*. *mansonoides*,* S*. *pretoriensis* and* S*. *theileri*) [7, 8]. Additionally, there is a relatively rare form called proliferative sparganosis, which is caused by the parasite *Sparganum proliferum*, a diphyllobothriidean cestode distinct from, yet similar to, *Spirometra* tapeworms [9–11].

**Fig. 1:**
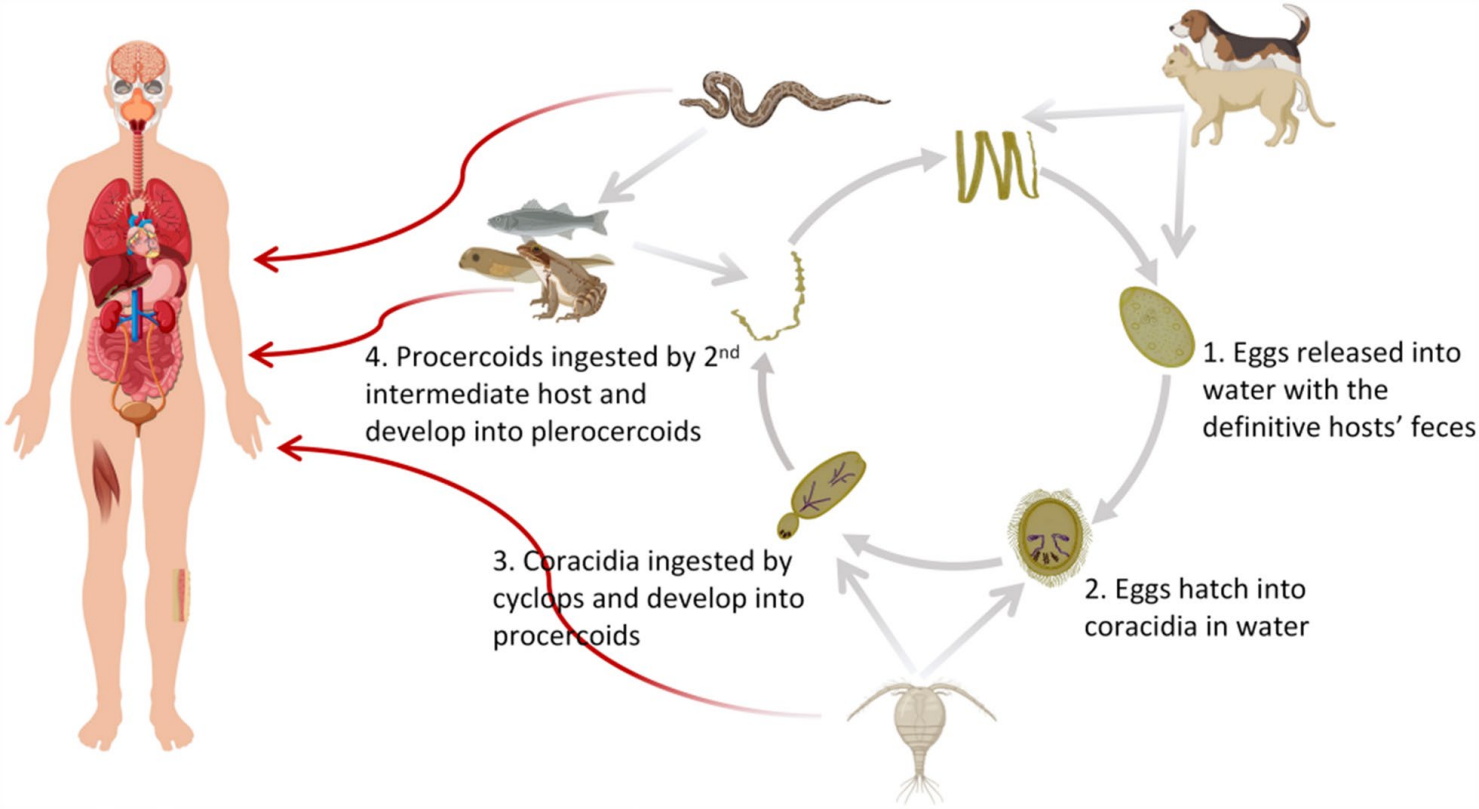
Life cycle of *Spirometra* spp. This complex cycle involves three larval stages, two intermediate hosts and several paratenic hosts, as follows: Stage 1: Adult tapeworms reside in the small intestine of definitive carnivorous mammalian hosts (e.g., cats and dogs), releasing immature eggs via feces into aquatic environments, where the eggs hatch into coracidia. Stage 2: Coracidia are ingested by the first intermediate hosts (copepods, e.g., *Cyclops* spp.) and develop into procercoid larvae within their body cavities. Stage 3: When infected copepods are consumed by the second intermediate hosts (e.g., amphibians or fish), procercoid larvae penetrate the intestinal wall of the second hosts and migrate to subcutaneous tissues or muscles, where they develop into plerocercoid larvae (spargana). Predators of these amphibians (e.g., reptiles) may act as paratenic hosts, harboring infective plerocercoids without further development. Stage 4: Definitive hosts become infected when they ingest the second intermediate hosts or paratenic hosts containing viable plerocercoids. Within the host’s small intestine, the plerocercoids mature into adult tapeworms, completing the cycle through egg production

The life cycle of *Spirometra* spp*.* requires two intermediate hosts and a definitive host, in addition to commonly involving paratenic hosts [4, 7]. The core developmental stages include the egg, coracidium, procercoid, and plerocercoid stages. Larvae do not multiply within intermediate hosts; they develop into adult worms and produce eggs exclusively in the definitive host (Fig. 1). The life cycle of *S. proliferum* remains incompletely characterized but is broadly assumed to follow a pattern comparable to that of *Spirometra* spp*..* Neither adult *S. proliferum* nor procercoids have been observed to date, leaving the mechanisms of human and mammalian infection unresolved [9]. Humans can be infected by plerocercoids through three main routes: (1) waterborne (ingestion of untreated water containing cyclops harboring procercoid larvae); (2) foodborne (ingestion of undercooked meat of second intermediate hosts, such as carnivores, amphibians, reptiles, rodents, or other mammals, harboring plerocercoid larvae); and (3) contact (local application of raw flesh of infected animals, such as frogs and snakes, on the skin or mucosa). Moreover, a rare case of neonatal sparganosis was documented in China in which plerocercoids were transmitted from an infected mother to her infant during pregnancy, confirming vertical transmission as an additional infection route for the disease [12]. Plerocercoids can invade various sites in humans, including the central nervous system (CNS), subcutaneous tissue, visceral organs, eyes, oral and maxillofacial regions, and the urogenital system. Depending on the site of infection, sparganosis can manifest as subcutaneous masses, pleural effusion, seizures, headaches, epilepsy, consciousness disorders, paralysis, blindness, and, in severe cases, death [13]. The incubation period of sparganosis can vary significantly, ranging from a few days to several years. This variability often causes the disease to be overlooked, resulting in delayed treatment. Additionally, nonspecific clinical symptoms frequently cause sparganosis to be confused with tumours and other parasitic diseases, increasing the risk of missed or incorrect diagnoses [14, 15]. The parasitic sites affected by *S. proliferum* are similar to those targeted by *S. mansoni* and *S. erinaceieuropaei*. However, unlike these species, *S. proliferum* exhibits unique capabilities for disseminated migration to various tissues throughout the body, where it undergoes budding proliferation. Affected patients frequently develop progressive emaciation and prostration, with severe cases becoming life-threatening and having an extremely poor prognosis [9].

Although rIDPs have gained increasing attention in recent years, they remain at the margins of the global health agenda. Owing to its sporadic nature and absence of large-scale outbreaks, human sparganosis has long been overlooked in terms of public health priorities. Notably, significant knowledge gaps persist in the global epidemiology of human sparganosis. Given that rIDPs disproportionately affect impoverished populations, there is an urgent need for targeted health interventions and evidence-based resource allocation in regions occupied by these populations to effectively control rIDPs and advance the elimination of all forms of poverty. This scoping review aims to systematically synthesize data on the global distribution data, basic demographic characteristics, infection sites, modes of transmission and diagnostic methods for human sparganosis, thereby addressing this critical evidence gap. Concurrently, it seeks to catalyze international attention and resource mobilization for this neglected disease while promoting the integration of a One Health approach into strategies for achieving the Sustainable Development Goals (SDGs). By bridging critical knowledge gaps, this work can inform policy decisions that align disease control with poverty alleviation, thereby supporting global sustainability agendas.

## Methods

This scoping review was conducted following the guidelines outlined in the Preferred Reporting Items for Systematic Reviews and Meta-Analyses-Extension for Scoping Reviews (PRISMA-ScR) [16].

### Search strategy, inclusion criteria and exclusion criteria

We systematically searched the China National Knowledge Infrastructure (CNKI, https://www.cnki.net/), WanFang (https://www.wanfangdata.com.cn/), PubMed (https://pubmed.ncbi.nlm.nih.gov/), Web of Science (https://www.webofscience.com/), Scopus (https://www.scopus.com/) and Embase (https://www.embase.com/) databases with no limits on the year of publication or language. The search keywords included “lietouyou”, “diegongshu”, “diegongtaochong”, “sparganosis”, “spirometra” and “plerocercoid”. The search terms used within the 6 databases are listed in Additional file 1. The last search was conducted on April 30, 2025.

The studies included case reports, dissertations, reviews and meeting abstracts, and excluded articles without full-text, comments and letters. The literature search and selection process were conducted by three independent researchers, following the steps given below: First, the retrieved documents were all imported into NoteExpress version 3.9.0.9575 (Beijing Aegean Sea Software Co., Ltd., Beijing, China), and duplicates were removed using the software’s duplicate checking function. Second, the titles and abstracts of relevant studies were screened, and articles that did not report human sparganosis cases or the prevalence of human sparganosis were excluded. Finally, the author, author’s affiliation and basic case information were used to exclude possible duplicate cases. Any disagreements encountered at this stage were resolved through consultation with another researcher or team discussion until a consensus was reached.

### Quality assessment of the included literature

The Joanna Briggs Institute Prevalence Critical Appraisal Tool was used for quality assessment of the articles[17]. Articles were scored according to the tool’s recommendations for ten quality control items, with 1 point given for completed items and 0 points given for incomplete items, which in turn classified studies as low (0–3), moderate (4–6) or high (7–10). The quality assessment report for all the articles included in this scoping review is provided in Additional file 2.

### Data extraction and analysis

The data were extracted from the included literature by one reviewer, and a secondary review of the extracted data was conducted by two other reviewers. The extracted information, which included the date of publication, time of diagnosis, regional distribution, age, sex, site of infection, risk factors, diagnostic methods, treatment, presence or absence of parasites, and misdiagnosis, was analyzed in Microsoft Excel 2021 (Microsoft Corp., Redmond, WA, USA). The study results are presented in figures, tables and narratives for descriptive analysis. Continuous variables were compared between two groups using independent samples *t*-test, after verifying normality and homogeneity of variance with Levene’s test. Associations between categorical variables were assessed using Pearson’s chi-square test. Statistical significance was defined as *P* < 0.05 for all analyses.

## Results

### Description of included studies

**Fig. 2:**
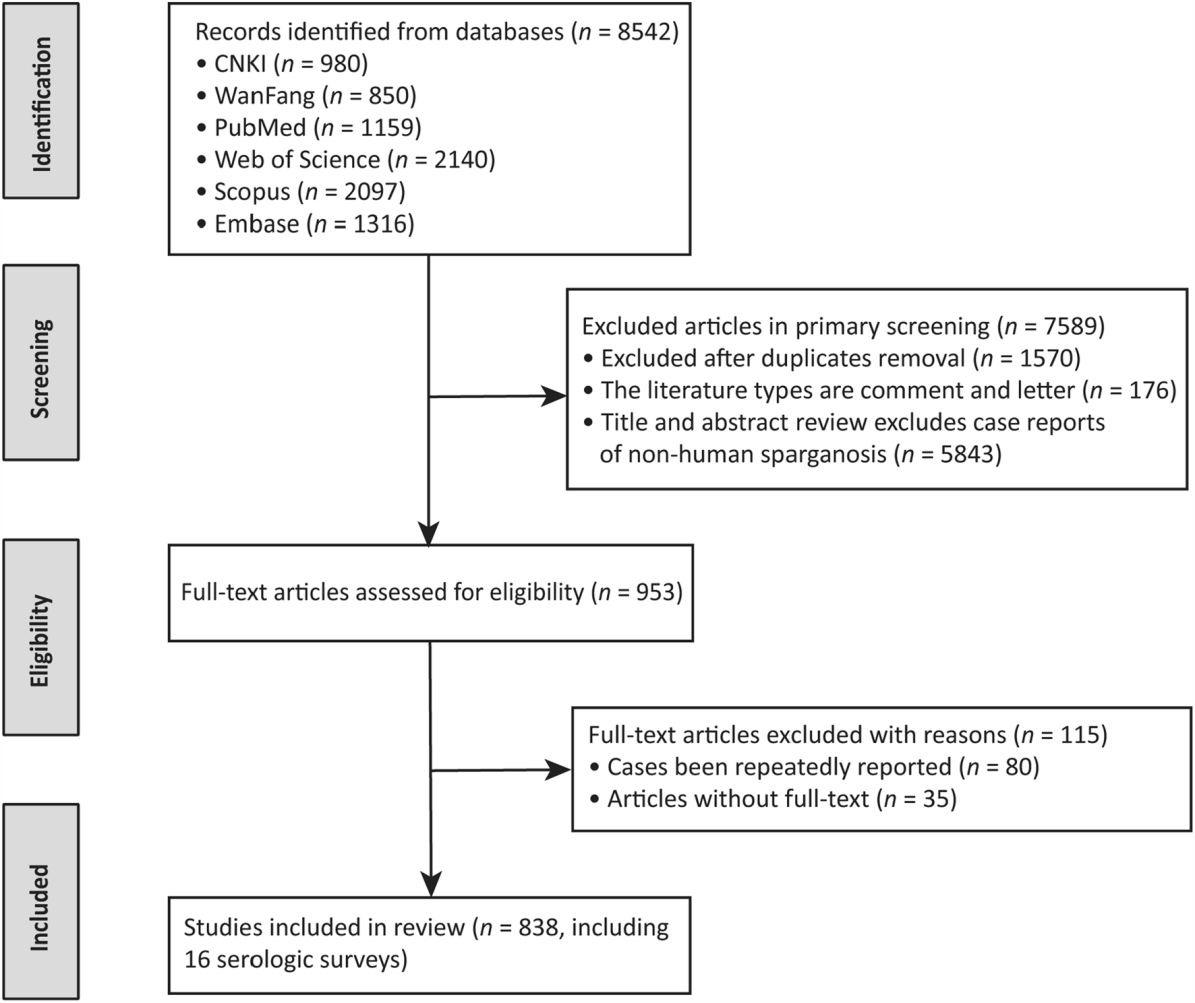
Flow diagram of the literature screening and selection process

In total, 8542 articles were identified through database searching [CNKI (*n* = 980), Wanfang (*n* = 850), PubMed (*n* = 1159), Web of Science (*n* = 2140), Scopus (*n* = 2097), and Embase (*n* = 1316)]. After the removal of 1570 duplicated records, 6972 articles were screened, of which 953 met the inclusion criteria. Following the full-text eligibility assessment, 838 articles were ultimately included in the review, including 16 articles related to serological surveys (Fig. 2). Across 822 articles, a comprehensive total of 3472 valid cases were reported, while the 16 serological survey articles involved 3148 positive subjects. All valid cases exhibited a clear country-level distribution. Among the cases documented in China, 1416 valid cases were accurately mapped to specific provinces. A total of 674 valid cases included a precisely recorded date of diagnosis. A total of 1176 valid cases contained explicit demographic information regarding age and sex. A total of 1968 valid cases documented both patient sex and infection site, and 1265 valid cases concurrently described both modes of transmission and infection sites.

### General characteristics of the included articles

Since 2000, a significant volume of literature has been published on human sparganosis, with more than 90.0% of the articles originating from the Asian regions (Table 1).

**Table 1:** General characteristics of the included articles

	Articles on valid cases number (%)	Articles on serological surveys number (%)
Year of publication		
Before 2000	276 (33.6)	1 (6.2)
after 2000	546 (66.4)	15 (93.8)
Continents		
Asia	757 (92.1)	15 (93.8)
Africa	7 (0.9)	1 (6.2)
South America	15 (1.8)	0 (0.0)
North America	25 (3.0)	0 (0.0)
Europe	14 (1.7)	0 (0.0)
Oceania	4 (0.5)	0 (0.0)

### Geographical distribution of human sparganosis

All valid case records specify country-level locations across all six continents (Table 2): Asia (95.8%, *n* = 3326), Africa (1.4%, *n* = 49), South America (0.6%, *n* = 21), North America (1.7%, *n* = 58), Europe (0.4%, *n* = 14), and Oceania (0.1%, *n* = 4). Asia remains the epicentre of cases, with China and the Republic of Korea together accounting for 90.4% of confirmed infections globally.

**Table 2:** Global distribution of human sparganosis

Continents	Cases (*n*)	Countries (cases)
Asia	3326	China (2381), Republic of Korea (757), Democratic People’s Republic of Korea (6), Japan (48), Thailand (91), India (27), Nepal (4), Viet nam (1), Bangladesh (1), Sri Lanka (4), Lao PDR (1), Philippines (4), Timor-Leste (1)
Africa	49	Egypt (1), Tanzania (7), Uganda (1), Rwanda (1), Ethiopia (4), South Sudan (34), Zimbabwe (1)
South America	21	Peru (6), Argentina (3), Brazil (2), Uruguay (1), Ecuador (3), Venezuela (2), Colombia (1), Paraguay (3)
North America	58	United States of America (55), Cuba (2), Mexico (1)
Europe	14	United Kingdom (1), Poland (1), Germany (2), Italy (2), France (3), Belgium (1), Spain (1), Czech Republic (1), Greece (1), Russia (1)
Oceania	4	Australia (4)

Among the confirmed cases documented in China, 1416 clearly recorded information on their provinces (Table 3). These cases were distributed across the majority of provinces, with the exception of Inner Mongolia, Gansu, Shaanxi, and Tianjin. A total of 78.0% of the cases were concentrated in eastern, central and southern China. The five provinces with the highest case concentrations were Guangdong, Hunan, Jilin, Jiangxi and Fujian.

**Table 3:** Geographical distribution of human sparganosis in China

Area	Cases (*n*)	Provincial-level administrative divisions (cases, *n*)
North China	19	Beijing (10), Hebei (7), Shanxi (2)
Northeast China	131	Jilin (93), Liaoning (36), Heilongjiang (2)
East China	405	Shanghai (47), Jiangsu (50), Zhejiang (76), Anhui (31), Fujian (85), Jiangxi (87), Shandong (5), Taiwan (24)
Central China	315	Henan (75), Hubei (51), Hunan (189)
South China	385	Guangdong (227), Guangxi (82), Hainan (59), Hong Kong (16), Macau (1)
Southwest China	156	Chongqing (66), Sichuan (32), Guizhou (33), Yunnan (24), Xizang (1)
Northwest China	5	Qinghai (3), Ningxia (1), Xinjiang (1)

### Temporal trends

**Fig. 3:**
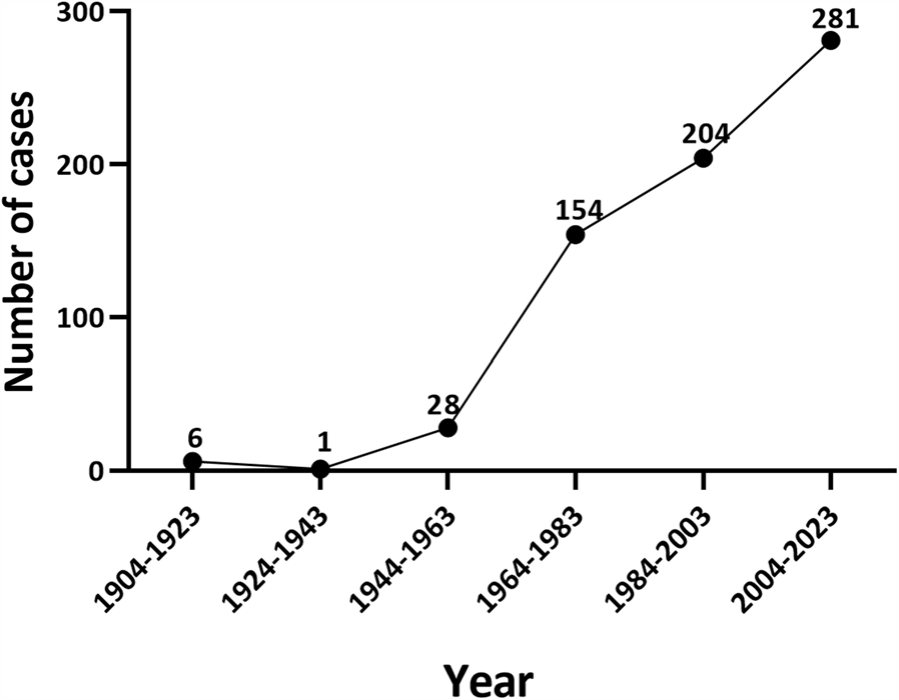
Number of cases reported during different periods

The analysis included 674 valid cases with documented diagnosis dates between 1904 and 2023 (Fig. 3).

### Demographic characteristics: age and sex

**Fig. 4:**
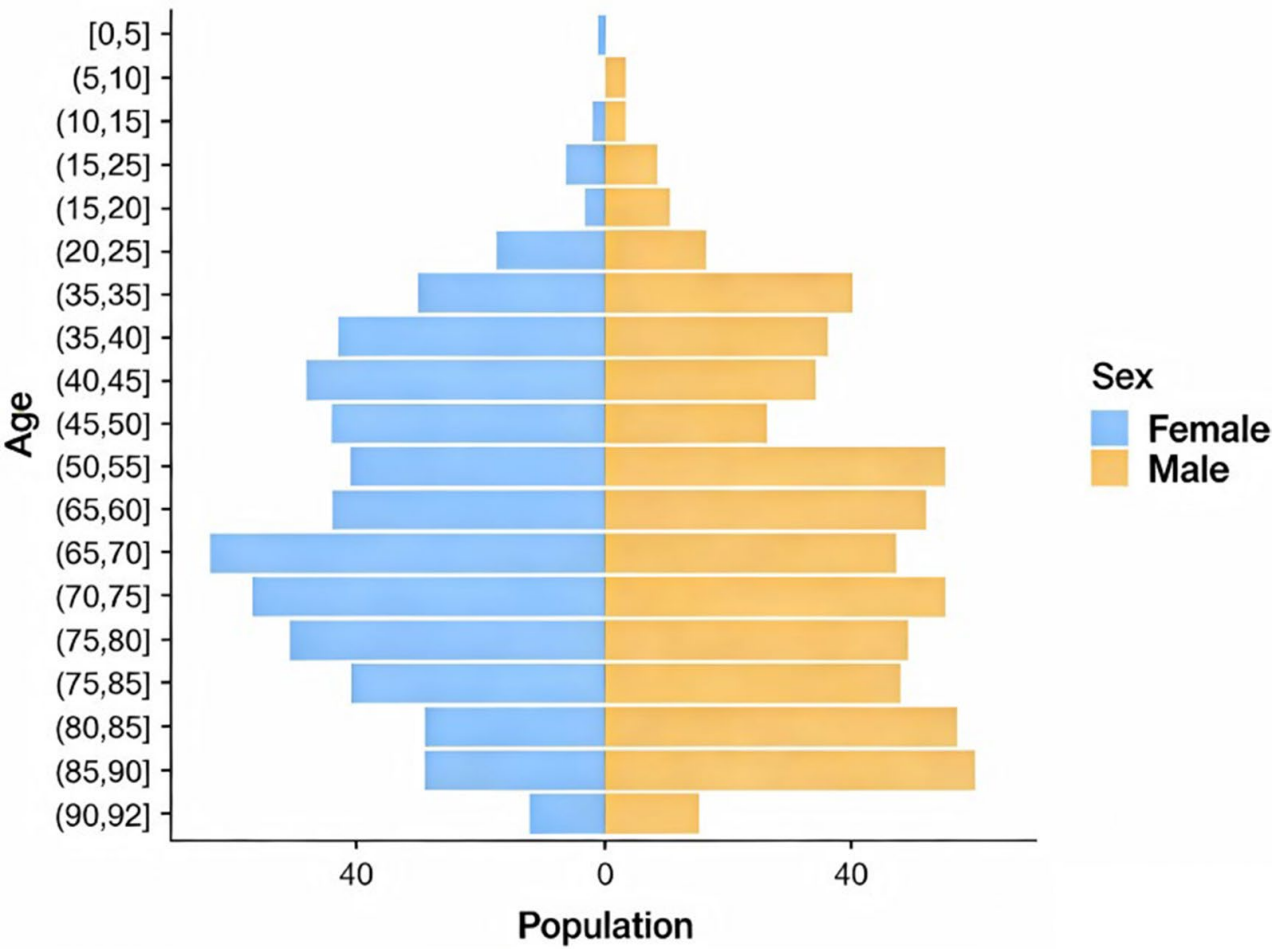
Population data and age-sex pyramid

No obvious difference in sex distribution was found (614 males, 562 females) among the 1176 valid cases with definite age and sex records (Fig. 4). The patients’ ages ranged from newborn to 92 years, and the average age was 35.8 years. The majority of patients were in the 5- to 65- year age group, accounting for 91.8% of the total. The independent samples *t*-test revealed a statistically significant difference in age between male and female patients (*P* < 0.05). Compared with female patients (*M* = 37.0, 95% *CI*: 35.56–38.53), male patients presented a lower mean age (*M* = 34.7, 95% *CI*: 33.16–36.32), with a mean difference of $-$ 2.3 years. The effect size was negligible (Cohen’s *d* = 0.121), indicating that sex was associated with only 0.37% of the age variance, with limited practical significance.

### Relationship of tissues/organs with sex

**Fig. 5:**
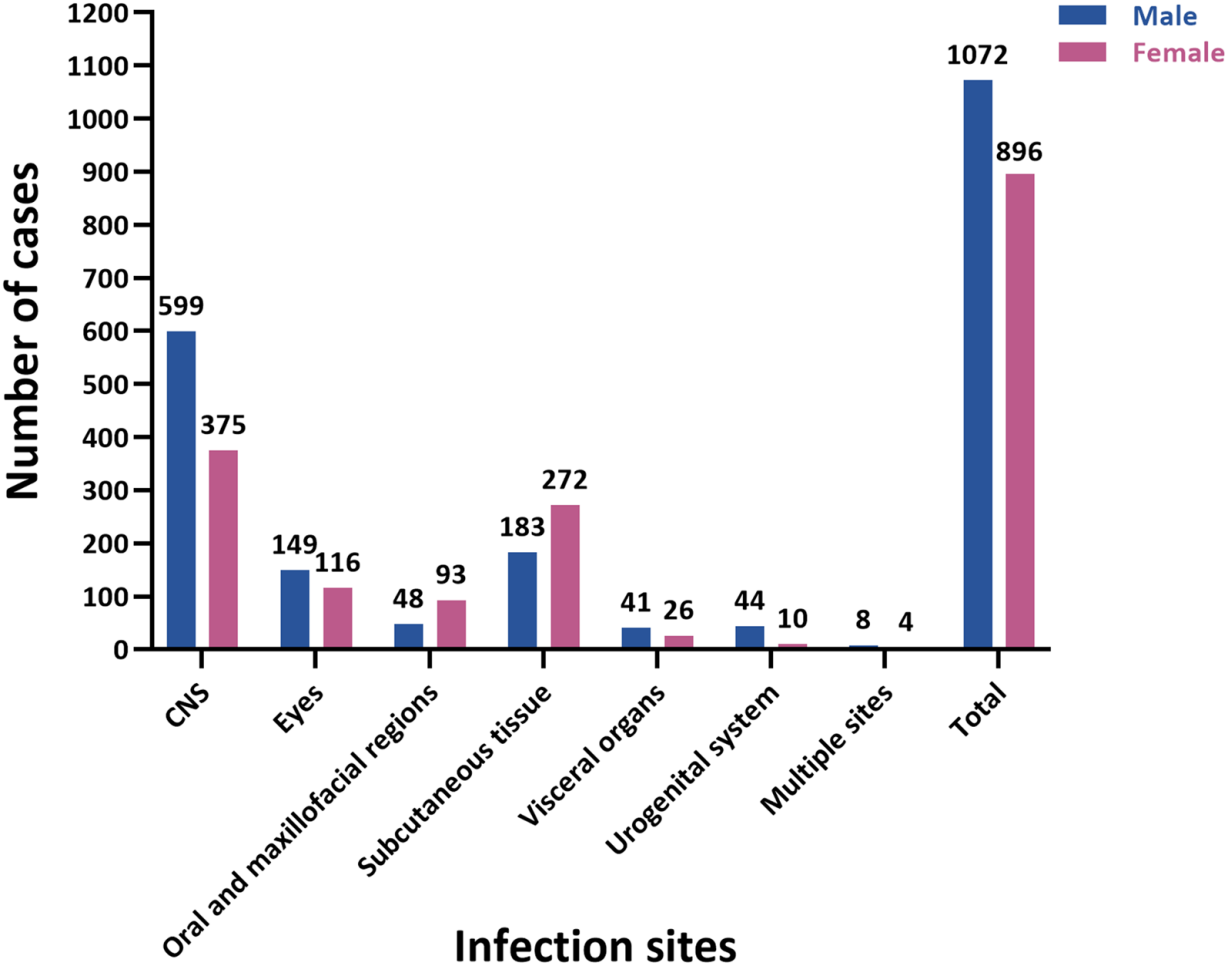
Distribution of infection sites by sex. *CNS* Central nervous system

The infection sites and sex distributions were analyzed in the included 1968 valid cases (Fig. 5). Pearson’s chi-square test revealed a statistically significant difference in infection site distribution between the sexes (*χ*^*2*^ = 98.54, *P* < 0.001). The CNS is the most frequently involved site in male and female patients, followed by the subcutaneous and ocular regions. Male predominance was observed in the CNS and in ocular, visceral, and genitourinary sparganosis, whereas female predominance was characterized by subcutaneous and oral-maxillofacial manifestations. All the results had expected frequencies > 5, thus meeting the test assumptions.

### Relationship of modes of transmission with tissues/organs

**Fig. 6:**
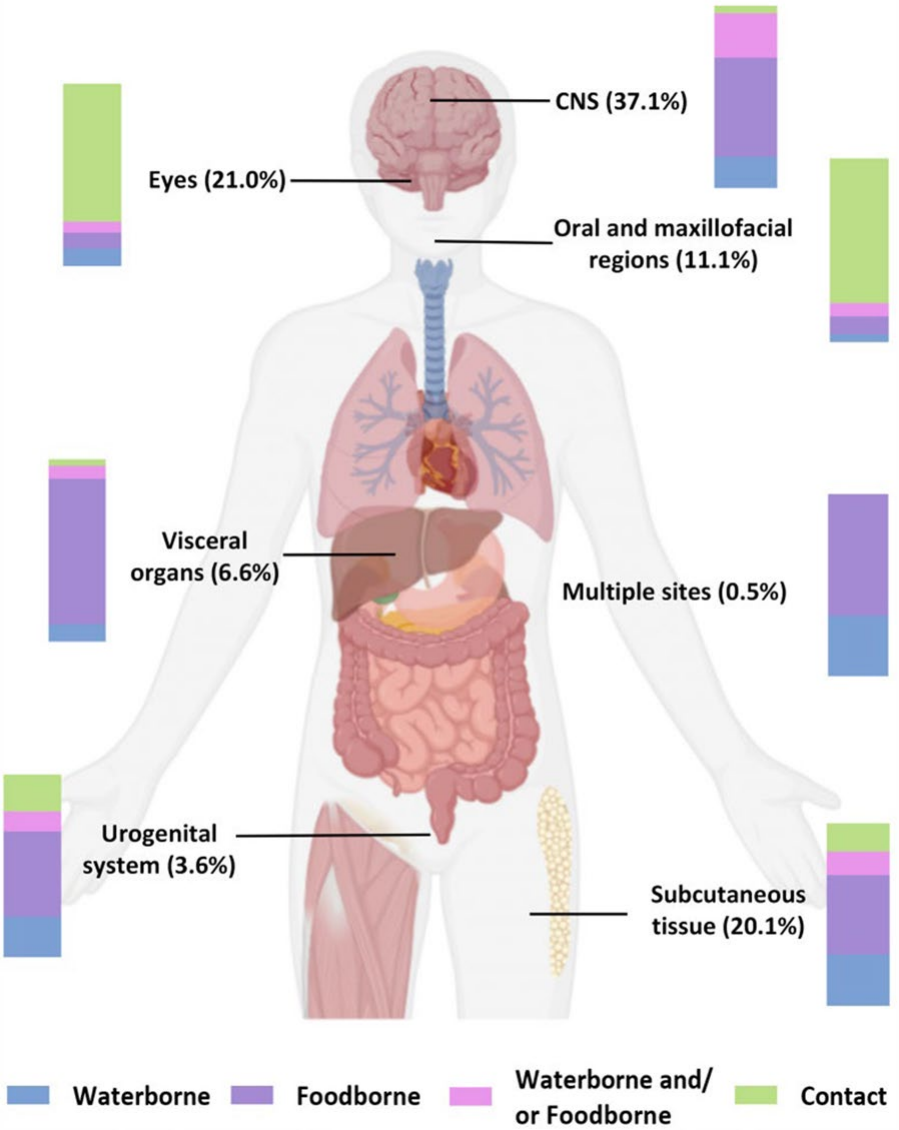
Anatomic distribution and modes of transmission of human sparganosis. The human organ diagram annotated with the proportional distribution (%) of infection sites. The stacked bar chart indicates the proportion of the modes of transmission (Blue: waterborne, purple: foodborne, pink: waterborne and/or foodborne, green: contact) at different sites of infection. *CNS* Central nervous system

The modes of transmission and tissues/organs were analyzed in the 1265 included valid cases (Fig. 6, Table 4). The most commonly involved sites were the CNS (37.1%), followed by the eyes (21.0%), subcutaneous tissue (20.1%), oral and maxillofacial regions (11.1%), visceral organs (6.6%), and the urogenital system (3.6%). Additionally, 0.5% of the cases involved multiple sites. Foodborne transmission (39.0%) was the primary transmission route, followed by contact (30.2%), and waterborne transmission (16.2%), waterborne and/or foodborne transmission (14.6%). Contact transmission was found mainly in China (98.7%, 377/382). Pearson’s chi-square test (after excluding multiple sites) revealed a significant association between infection site and transmission route (*χ*^*2*^ = 701.36; *P* < 0.001). CNS infection is associated with foodborne and waterborne transmission. Ocular and oral-maxillofacial infections are linked to contact transmission. Subcutaneous and visceral infections are correlated with waterborne and foodborne transmission. All results excluding multiple sites had expected frequencies > 5, thus meeting the test assumptions (Table 4).

**Table 4:** Relationship of the modes of transmission with tissues/organs

Sites of infection	Waterborne (%)	Foodborne (%)	Waterborne and/or foodborne (%)	Contact (%)	Total (%)
CNS	81 (17.3)	254 (54.1)	115 (24.5)	19 (4.1)	469 (100.0)
Eyes	26 (9.8)	23 (8.6)	16 (6.0)	201 (75.6)	266 (100.0)
Oral and maxillofacial regions	6 (4.3)	14 (9.9)	10 (7.1)	111 (78.7)	141 (100.0)
Subcutaneous tissue	72 (28.3)	110 (43.3)	33 (13.0)	39 (15.4)	254 (100.0)
Visceral organs	8 (9.5)	67 (79.8)	6 (7.1)	3 (3.6)	84 (100.0)
Urogenital system	10 (22.2)	21 (46.7)	5 (11.1)	9 (20.0)	45 (100.0)
Multiple sites	2 (33.3)	4 (66.7)	0 (0.0)	0 (0.0)	6 (100.0)
Total	205 (16.2)	493 (39.0)	185 (14.6)	382 (30.2)	1265 (100.0)

### Diagnosis of human sparganosis

With the exception of cases in which the diagnostic methods employed were not specified, the majority of valid cases (61.9%, 2149/3472) were diagnosed by optical microscopy and/or molecular biological techniques. By contrast, 25.3% (878/3472) of the sparganosis cases were diagnosed on the basis of clinical manifestations, imaging, and/or immunological findings. Molecular biological techniques specifically confirmed sparganosis in 93 study subjects (Table 5). Polymerase chain reaction (PCR) technology was utilized in 87 cases, and next-generation sequencing (NGS) technology was employed in 6 cases. After excluding 38 cases with unspecified infection sites, the remaining cases were distributed as follows: 33 in the subcutaneous region, 14 in the CNS, 4 in the ocular region, 2 in visceral organs, and 2 in multiple sites. Molecular testing was performed on pathological tissue samples from 21 patients, bronchoalveolar lavage fluid from 1 patient, and on the worm itself in the remaining patients.

**Table 5:** Molecular biological techniques for human sparganosis. *CNS* Central nervous system, *NGS* next-generation sequencing

Countries	Total cases	Site of infection (cases)	Techniques (cases)	Samples (cases)	References
China	28	CNS (6), subcutaneous tissue (17), eyes (2), visceral organs (1), multiple sites (1), unknown (1)	NGS (6), PCR (22)	Tissue (5), worm (22), bronchoalveolar lavage fluid (1)	[18–31]
Japan	7	Subcutaneous tissue (4)	PCR (4)	Tissue (4), worm (3)	[32–36]
Republic of Korea	2	CNS (1), multiple sites (1)	PCR (2)	Worm (2)	[37, 38]
Thailand	16	CNS (6), subcutaneous tissue (7), visceral organs (1), eyes (2)	PCR (16)	Tissue (11), worm (4), unknown (1)	[39–44]
Egypt	1	Subcutaneous tissue (1)	PCR (1)	Worm (1)	[45]
Ethiopia	3	Unknown (3)	PCR (3)	Worm (3)	[46]
South Sudan	34	Unknown (34)	PCR (34)	Worm (34)	[46]
Bangladesh	1	CNS (1)	PCR (1)	Tissue (1)	[47]
Germany	1	Subcutaneous tissue (1)	PCR (1)	Worm (1)	[48]

### Serological surveys on human sparganosis

A total of 16 serological survey studies involved 93,660 participants, of whom 3148 tested positive (Table 6). These studies were confined to three countries: China, the Republic of Korea, and Tanzania. The majority (93.8%, 15/16) of these studies were conducted in China and the Republic of Korea. In Tanzania, the highest percentage of the study population was positive (62.5%)[49]. Study 12 indicated that residents who participated in serological testing also exhibited a high positive rate (11.4%) in Whachon-gun, Republic of Korea[50]. In Studies 4 and 2, serum samples were obtained from hospitals and exhibited high positive rates (21.4% and 17.5%, respectively)[51, 52]. In Study 1, the positive rate of subjects with a history of raw tadpole consumption was 9.0% (17/189), which was significantly higher than that of those without high-risk behaviors (2.4%, 5/213)[53]. In Study 10, the serum positive rate was 2.5% among epileptic patients and 1.9% among normal adults, with no significant difference observed between the two groups[54].

**Table 6:** Geographic and temporal distribution of serological surveys for human sparganosis worldwide

Study ID	Country	Region/Province	Year	Sample Size (*n*)	Positive rate (%)	References
1	China	Henan	2006–2015	402	5.5	[53]
2	China	NA	2011–2013	730	17.5	[52]
3	China	Guangdong	2013	750	4.5	[55]
4	China	NA	2014–2017	1811	21.4	[51]
5	China	Guangdong	2015–2016	757	4.6	[56]
6	China	Henan	2015	298	5.7	[57]
7	China	Guangdong	2017–2019	2058	5.5	[58]
8	China	Henan	2023	649	0.2	[59]
9	China	Zhejiang	Na	136	0.7	[60]
10	Republic of Korea	Nationwide	1987–1990	3517	2.3	[54]
11	Republic of Korea	NA	1993–2006	74,448	2.5	[61]
12	Republic of Korea	Kangwon-do	1995–1999	316	11.4	[50]
13	Republic of Korea	Seoul	1996–2006	6017	4.4	[62]
14	Republic of Korea	Gangweon-do, Hongcheon-gun	1999	719	3.3	[63]
15	Republic of Korea	Cheorwon-gun, Goseong-gun and Ongjin-gun	2007–2014	836	4.2	[64]
16	Tanzania	Monduli, Babati	Na	216	62.5	[49]

a: Seoul, Inchon, Kyonggi, Kangwon, Taejon, Chungbuk, Chungnam, Chunbuk, Chonnam, Kyongbuk, Kyongnam, Cheju. *NA* Not available

## Discussion

Human sparganosis is a neglected foodborne/waterborne zoonosis and an rIDP with dynamic transmission patterns. Our scoping review reveals concordance between the observed geographic distribution of human sparganosis and that described in prior literature: hyperendemicity in East and Southeast Asia contrasts with sporadic occurrence elsewhere. Foodborne transmission represents the primary infection route. Crucially, contact transmission via the application of frog or snake poultices constitutes a distinctive epidemiological pattern concentrated in China, differing significantly from that in other countries. The period from 2004 to 2023 showed the highest case frequency. However, cases with clearly documented diagnostic dates accounted for only 18.0% (674/3472) of all valid cases, failing to provide an explanation for the overall temporal trend.

Only 8 articles on human sparganosis cases in Africa were retrieved, accounting for 1.0% of the total included articles. There were 37 cases of human sparganosis in Ethiopia and South Sudan between 2012 and 2014, accounting for the majority (75.5%) of all documented African cases [46]. However, we speculate that human sparganosis may be a relatively common parasitic disease in this region. A serosurvey of anti-sparganum IgG antibodies in rural Tanzania revealed a high seroprevalence (62.5%) among participants, most of whom (94.9%) drank unboiled water from running springs and rivers [49]. These studies not only indicate the potential for more human sparganosis cases in Africa but also reveal that drinking unboiled water may be a significant cause of sparganum infection in this region.

Sparganosis occurs primarily in the CNS. The migratory nature and nonspecific clinical presentation of CNS sparganosis often lead to misdiagnosis, with an initial misdiagnosis rate as high as 62.0%, posing significant diagnostic and therapeutic challenges [14]. In this retrospective study, 143 cases of CNS sparganosis were documented as misdiagnosed as glioma, astrocytoma, metastatic brain tumors, cerebral lymphoma, dysembryoplastic neuroepithelial tumor, tuberculoma, inflammatory granuloma, encephalomyelitis, cerebrovascular lesions, intracranial infection, intracerebral hemorrhage, or cerebral infarction (see Additional file 3). Clinical physicians should improve their awareness of the clinical manifestations and imaging findings of CNS sparganosis, investigate patients’ epidemiologic histories, integrate routine blood tests, and promptly perform parasite-specific serology screening to minimize misdiagnosis rates.

The definitive diagnosis of human sparganosis was confirmed by optical microscopy. However, significant limitations arise when plerocercoids inhabit deep tissues (e.g., the CNS), necessitating the use of supplementary diagnostic techniques [65, 66]. Immunodiagnostic and imaging methods were used for auxiliary diagnosis. Medical imaging (e.g., computed tomography, magnetic resonance imaging, and ultrasound) facilitates diagnosis by revealing characteristic features, thereby increasing the detection rates compared with optical microscopy methods, which have inherent limitations [67]. Importantly, the limited specificity of medical imaging, in conjunction with its substantial cost, restricts its utility for the diagnosis of early/mild infections and for rapid screening.

Immunological techniques for the diagnosis of sparganosis offer a minimally invasive, sensitive, and cost-efficient alternative, providing value for early-stage, latent, or deep-tissue infections and supporting epidemiological surveillance [53, 64, 68, 69]. We retrieved 16 studies that employed immunological diagnostic methods to assess the seroprevalence of sparganosis across diverse populations. The findings indicated that the total number of seropositive subjects (*n* = 3148) approached that of the valid cases (*n* = 3472), representing a substantial underestimation of the global sparganosis burden when assessed solely from the perspective of case distribution. Furthermore, the results of serological surveys demonstrated higher positive rates among populations at high risk of sparganosis. Immunodiagnostic methods enable cost-effective mass screening in resource-limited settings while efficiently detecting asymptomatic and early infections, thereby offsetting the need for more scarce imaging and pathological diagnostics in impoverished areas [70, 71]. Thus, in high-risk populations and endemic regions (e.g., communities with specific dietary practices), immunodiagnostic methods facilitate the targeted allocation of constrained medical resources and the quantification of subclinical infection rates to refine epidemiological data. Notably, the potential for cross-reactivity with other parasites necessitates careful interpretation of positive results, in conjunction with epidemiological data [72–74].

The advancement of molecular biological techniques (e.g., PCR, DNA sequencing, and omics) provides a new approach for the accurate diagnosis of infectious diseases in clinical practice and advances taxonomic research on *Spirometra* cestodes [18, 22, 24, 44, 75]. For pathological biopsies lacking discernible morphological features, where parasitic involvement is suspected but the species is unidentifiable, these molecular biological techniques enable precise identification [18, 29]. Among the cases included in the study, 93 were subjected to molecular diagnostic techniques, including metagenomics and PCR. In addition, molecular diagnosis at the species level is becoming increasingly important for clarifying the life cycle and transmission routes of *Spirometra* spp. responsible for human sparganosis [37]. However, the clinical scalability of these techniques is hindered by technical complexity, lengthy processing times, and prohibitive costs.

Currently, surgical removal of the parasite is considered the primary treatment for sparganosis. However, the choice of treatment is a challenge for inoperable patients, including those with multifocal and surgically contraindicated lesions. Some studies have suggested that long-term and high-dose praziquantel therapy (e.g., 50 mg/kg per day for 10 days, 75 mg/kg per day for 7 days, 75 mg/kg per day for 10 days, and 50 mg/kg per day for 10 days repeated monthly) is effective and represents a promising alternative for inoperable patients with sparganosis [47, 76–78]. Given the limitations inherent to these studies, including small sample sizes, highly variable patient clinical data, and heterogeneous treatment regimens, the therapeutic value of long-term and high-dose praziquantel for sparganosis remains to be adequately evaluated.

The One Health approach is crucial because *Spirometra* tapeworms have a broad host range and have been documented in domestic and wild animals across the globe except Antarctica, with high infection rates even in regions with fewer human cases [79–84]. Tailored strategies are needed for endemic and nonendemic regions. In endemic regions, such as China and the Republic of Korea, interventions should focus on the life cycle of the parasite. These interventions may include monitoring water sources for cyclops; surveilling intermediate and definitive hosts (e.g., frogs, snakes, cats and dogs) to minimize environmental contamination; conducting public health campaigns to discourage the consumption of raw or undercooked food; and ensuring cross-sectoral collaboration among veterinary, environmental, and health authorities to monitor host populations and habitat quality. In regions where the disease is not endemic, the focus of these measures shifts to the mitigation of importation risks [85, 86]. This involves strengthening of border inspections of wildlife products and aquaculture imports, educating travellers and immigrant communities on potential dietary hazards, and training clinicians to recognize imported cases.

The scarcity of data on sparganosis remains a key barrier to controlling and eliminating this disease. The establishment of an efficient and timely surveillance-response system supported by reliable disease prevalence and spatiotemporal distribution data, with the capacity for rapid intervention, represents a decisive pathway toward effective sparganosis prevention, control, and elimination [87, 88]. Furthermore, innovative technologies such as geographic information systems, global positioning systems, and geostatistical modeling need to be integrated into these surveillance-response systems [89, 90]. Ultimately, standardized diagnostic instruments, cross-border collaboration, and community engagement are imperative for bridging these gaps, reducing transmission in endemic zones and preventing spillover in nonendemic zones.

Although this scoping review provides updates on the spatiotemporal distribution characteristics of human sparganosis, knowledge gaps still persist. First, the literature retrieval was retrieved from the CNKI, Wanfang, PubMed, Web of Science, Scopus and Embase databases. Regional databases (e.g., Latin American and Caribbean Literature on Health Sciences for Latin America and African Journals Online and African Index Medicus for Africa) were not included in this retrieval. This potentially resulted in the omission of studies published in nonindexed journals, thereby underestimating the actual infection burden. This may also result in a bias, whereby the burden is overestimated in Asian regions and underestimated in non-Asian regions. Additionally, the nonspecific clinical presentation coupled with prolonged incubation periods increases the risk of diagnostic oversight. Moreover, the dearth of research beyond Asia may signal underreporting and surveillance gaps in regions that receive less attention. Hence, targeted research and intervention planning in a global context are urgently needed, coupled with continuous optimizing control strategies and promoting health education.

## Conclusions

This scoping review offers updates on global distribution data and the current epidemiological situation of human sparganosis. China, the Republic of Korea and Thailand are the main epidemic countries. However, the incidence of human sparganosis in African regions (such as South Sudan, Tanzania and Ethiopia) may be underestimated. The primary routes of transmission are foodborne, with contact transmission predominantly occurring within China. The CNS is the site where infections occur most frequently. Our findings may receive increasing attention in endemic regions while increasing awareness in resource-limited nonendemic areas. These findings could help policymakers review human sparganosis control strategies, thereby alleviating public health burdens. This study calls for increased attention to rIDPs. A One Health approach is imperative for achieving the SDGs, with the objective of reducing the disease burden and enhancing human health and well-being.

## Supplementary Information

Additional file 1.

Additional file 2.

Additional file 3.

## Data Availability

Not applicable.

## Abbreviations

CNKI: China National Knowledge Infrastructure
rIDPs: Rare infectious diseases of poverty
CNS: Central nervous system
NA: Not available
NGS: Next-generation sequencing
SDGs: Sustainable development goals

## References

[1] Zhou X-N. Prioritizing research for “One health - One world.” Infect Dis Poverty. 2012;1(1):1. 10.1186/2049-9957-1-1. 23849840 10.1186/2049-9957-1-1 PMC3710101

[2] World Health O, Research UNUWBWSPf, Training in Tropical D. Global report for research on infectious diseases of poverty 2012. Geneva: World Health Organization; 2012.

[3] Bao YJ, Li YX, Zhou YB, Qiang N, Li TY, Zhang YZ, et al. Global burden associated with rare infectious diseases of poverty in 2021: findings from the Global Burden of Disease Study 2021. Infect Dis Poverty. 2024;13(1):85. 10.1186/s40249-024-01249-6. 39538351 10.1186/s40249-024-01249-6 PMC11558835

[4] Liu Q, Li MW, Wang ZD, Zhao GH, Zhu XQ. Human sparganosis, a neglected food borne zoonosis. Lancet Infect Dis. 2015;15(10):1226–35. 10.1016/s1473-3099(15)00133-4. 26364132 10.1016/S1473-3099(15)00133-4

[5] Ts C. Description of *Ligula mansoni*, a new human cestode. J Linn Soc Lond Zool. 1883;17(98):78–83.

[6] Lu G, Shi DZ, Lu YJ, Wu LX, Li LH, Rao LY, et al. Retrospective epidemiological analysis of sparganosis in mainland China from 1959 to 2012. Epidemiol Infect. 2014;142(12):2654–61. 10.1017/s0950268814000144. 25372227 10.1017/S0950268814000144 PMC9151273

[7] Liu W, Gong T, Chen S, Liu Q, Zhou H, He J, et al. Epidemiology, diagnosis, and prevention of sparganosis in Asia. Animals (Basel). 2022;12(12):1578. 10.3390/ani12121578. 35739914 10.3390/ani12121578 PMC9219546

[8] Mariaux J, Kuchta R, Hoberg EP. Tetrabothriidea Baer, 1954. In: Planetary biodiversity inventory (2008–2017): tapeworms from vertebrate bowels of the Earth*.* edn. Lawrence, KS (US): University of Kansas, Natural History Museum; 2017.

[9] Kikuchi T, Maruyama H. Human proliferative sparganosis update. Parasitol Int. 2020;75:102036. 10.1016/j.parint.2019.102036. 31841658 10.1016/j.parint.2019.102036

[10] Iijima I. On a new cestode larva parasitic in man (*Plerocercoides prolifer*). J Coll Sci Imp Univ Tokyo. 1905;20(7):1–21.

[11] Stiles CW. The occurrence of a proliferating cestode larva (*Sparganum proliferum*) in man in Florida, vol. 40; 1908.

[12] A case of newborn infant sparganosis. Chin J Paediatr. 1983(02):15. (in Chinese)

[13] Qiu MH, Qiu MD. Human plerocercoidosis and sparganosis: II. a historical review on pathology, clinics, epidemiology and control. Chin J Parasitol Parasit Dis. 2009;27(3):251–60. (in Chinese) 19852371

[14] Shi DM, Wang XL, Chen L, Xie Q. Clinical characteristics and misdiagnosis analysis of sparganosis: a retrospective study of 52 cases. J Diagn Concepts Pract. 2020;19(01):37–43. 10.16150/j.1671-2870.2020.01.009. (in Chinese)

[15] Shen MQ, Mao JQ. Sparganosis Mansoni with urticaria: a case report. China Prev Med J. 2015;27(09):933–4. 10.19485/j.cnki.issn1007-0931.2015.09.022. (in Chinese)

[16] Tricco AC, Lillie E, Zarin W, O’Brien KK, Colquhoun H, Levac D, et al. PRISMA extension for scoping reviews (PRISMA-ScR): checklist and explanation. Ann Intern Med. 2018;169(7):467–73. 10.7326/m18-0850. 30178033 10.7326/M18-0850

[17] Munn Z, Moola S, Riitano D, Lisy K. The development of a critical appraisal tool for use in systematic reviews addressing questions of prevalence. Int J Health Policy Manag. 2014;3(3):123–8. 10.15171/ijhpm.2014.71. 25197676 10.15171/ijhpm.2014.71 PMC4154549

[18] Pang CM, Yang XL, Wang Y, Zhai H, Miao F, Zhang SM. Metagenomic sequencing for diagnosis of sparganosis Mansoni: a case report. Chin J Schistosomiasis Control. 2022;34(05):556–8. 10.16250/j.32.1374.2022035. (in Chinese) 10.16250/j.32.1374.2022035 36464258

[19] Hu D, Jin W, Ding H, Pang Y, Ma S, Yang M, et al. *Spirometra mansoni* sparganosis identified by metagenomic next-generation sequencing: a case report. Int J Infect Dis. 2023;128:128–31. 10.1016/j.ijid.2022.12.038. 36592686 10.1016/j.ijid.2022.12.038

[20] Liu J, Zhang S, Guo H, Feng K, Jiang Q. Free-living sparganosis in the lumbosacral spine with long latency. Lancet Infect Dis. 2022;22(5):742. 10.1016/s1473-3099(22)00150-5. 35460664 10.1016/S1473-3099(22)00150-5

[21] Meng Y, Kuang Z, Liao L, Ma Y, Wang X. Case report: morphologic and genetic identification of cerebral sparganosis. Am J Trop Med Hyg. 2019;101(5):1174–6. 10.4269/ajtmh.19-0468. 31436160 10.4269/ajtmh.19-0468 PMC6838588

[22] Du B, Tao Y, Ma J, Weng X, Gong Y, Lin Y, et al. Identification of sparganosis based on next-generation sequencing. Infect Genet Evol. 2018;66:256–61. 10.1016/j.meegid.2018.10.005. 30315893 10.1016/j.meegid.2018.10.005

[23] Yang P, Zheng W, Wang L. Ultrasonographical and molecular diagnosis of breast sparganosis due to *Spirometra erinaceieuropaei*. Travel Med Infect Dis. 2022;49:102393. 10.1016/j.tmaid.2022.102393. 35752292 10.1016/j.tmaid.2022.102393

[24] Tang THC, Wong SSY, Lai CKC, Poon RWS, Chan HSY, Wu TC, et al. Molecular identification of *Spirometra erinaceieuropaei* tapeworm in cases of human sparganosis, Hong Kong. Emerg Infect Dis. 2017;23(4):665–8. 10.3201/eid2304.160791. 28322697 10.3201/eid2304.160791 PMC5367436

[25] Zhao Q, Li P, Peng R, Huang H, Ding X, Wang J, et al. Disseminated central nervous system sparganosis appearing as meningeal granulomatous disease diagnosed by next-generation sequencing. Neurol Sci. 2023;44(5):1823–6. 10.1007/s10072-022-06596-6. 36637624 10.1007/s10072-022-06596-6

[26] Bennett HM, Mok H, Gkrania-Klotsas E, Tsai IJ, Stanley EJ, Antoun NM, et al. The genome of the sparganosis tapeworm *Spirometra erinaceieuropaei* isolated from the biopsy of a migrating brain lesion. Genome Biol. 2014;15(11):510. 10.1186/preaccept-2413673241432389. 25413302 10.1186/s13059-014-0510-3 PMC4265353

[27] He L, Fang ZM, Xue T, Zhang EF, An CL. Genetic identification of *Spirometra erinaceieuropaei* spargana in Liaoning and Hubei Provinces, P.R. China. Korean J Parasitol. 2019;57(3):309–12. 10.3347/kjp.2019.57.3.309. 31284356 10.3347/kjp.2019.57.3.309 PMC6616165

[28] Xu YW, Cai K, Li HJ, Xu SS, Du HM, Yu HJ, et al. Pulmonary sparganosis in children: a case report and literature review. J Clin Pediatr. 2021;39(12):941–3+55. (in Chinese)

[29] Lan ZH, Su ZS, He M, Zhang ZP, Hu MJ, Zeng QR. Use specific sequences of mitochondrial gene to indentify two cases suspected plerocercoid infection pathological specimens with no worms. Chin J Clin Exp Pathol. 2013;29(07):753–6. 10.13315/j.cnki.cjcep.2013.07.002. (in Chinese)

[30] Qin LP, Lv J, Cheng N, Li JF, Xie LJ, Cheng MH. 18 F-FDG PET/ CT imaging in sparganosis mansoni: a case report. Chin J Nucl Med Mol Imaging. 2019;39(12):754–5. 10.3760/cma.j.issn.2095-2848.2019.12.011. (in Chinese)

[31] Ru SS, Cheng C, Jiang P, Zhang X. Identification of a clinical *Spirometra mansoni* plerocercoid isolate using molecular and morphological data. Acta Parasitol. 2024;69(2):1304–8. 10.1007/s11686-024-00836-9. 38536613 10.1007/s11686-024-00836-9

[32] Nonomura Y, Otsuka A, Endo Y, Fujisawa A, Nakajima N, Minamiguchi S, et al. *Sparganosis mansoni* on abdominal skin, mimicking folliculitis and diagnosed by analysis of the mitochondrial cytochrome c oxidase subunit 1 gene, using polymerase chain reaction. Eur J Dermatol. 2012;22(6):806–7. 10.1684/ejd.2012.1876. 23220202 10.1684/ejd.2012.1876

[33] Okino T, Yamasaki H, Yamamoto Y, Fukuma Y, Kurebayashi J, Sanuki F, et al. A case of human breast sparganosis diagnosed as *Spirometra* Type I by molecular analysis in Japan. Parasitol Int. 2021;84:102383. 10.1016/j.parint.2021.102383. 34044106 10.1016/j.parint.2021.102383

[34] Kudo T, Fujioka A, Korenaga M, Yamasaki H, Morishima Y, Sugiyama H, et al. Molecular identification of intramuscular and subcutaneous *Spirometra erinaceiuropaei* sparganosis in a Japanese patient. J Dermatol. 2017;44(6):e138–9. 10.1111/1346-8138.13739. 28106263 10.1111/1346-8138.13739

[35] Tappe D, Berger L, Haeupler A, Muntau B, Racz P, Harder Y, et al. Case report: molecular diagnosis of subcutaneous *Spirometra erinaceieuropaei* sparganosis in a Japanese immigrant. Am J Trop Med Hyg. 2013;88(1):198–202. 10.4269/ajtmh.2012.12-0406. 23166198 10.4269/ajtmh.2012.12-0406 PMC3541736

[36] Sonosaki T, Okubo Y, Omine T, Miyagi T, Kariya Y, Yamamoto YI, et al. Three cases of sparganosis mansoni : identification of the causative parasite species by PCR methods. Nishinihon J Dermatol. 2016;78(5):522–7. 10.2336/nishinihonhifu.78.522.

[37] Hwang YH, Son W, Kim YW, Kang DH, Chang HH, Goo YK, et al. A retrieved sparganum of *Spirometra erinaceieuropaei* from a korean man during mechanical thrombectomy. Korean J Parasitol. 2020;58(3):309–13. 10.3347/kjp.2020.58.3.309. 32615744 10.3347/kjp.2020.58.3.309 PMC7338899

[38] Yun SJ, Park MS, Jeon HK, Kim YJ, Kim WJ, Lee SC. A case of vesical and scrotal sparganosis presenting as a scrotal mass. Korean J Parasitol. 2010;48(1):57. 10.3347/kjp.2010.48.1.57. 20333286 10.3347/kjp.2010.48.1.57 PMC2843847

[39] Muigg V, Ruf MT, Schwarzkopf S, Huang S, Denisjuk N, Stürmann A, et al. Case report: human subcutaneous sparganosis in a Thai migrant. Am J Trop Med Hyg. 2019;101(5):1170–3. 10.4269/ajtmh.19-0456. 31571569 10.4269/ajtmh.19-0456 PMC6838579

[40] Chotmongkol V, Phuttharak W, Jingjit K, Chaisuriya N, Sanpool O, Chaichan S, et al. Case report: sparganosis of the cauda equina. Am J Trop Med Hyg. 2021;104(1):298–302. 10.4269/ajtmh.20-0712. 33124542 10.4269/ajtmh.20-0712 PMC7790082

[41] Boonyasiri A, Cheunsuchon P, Srirabheebhat P, Yamasaki H, Maleewong W, Intapan PM. Sparganosis presenting as cauda equina syndrome with molecular identification of the parasite in tissue sections. Korean J Parasitol. 2013;51(6):739–42. 10.3347/kjp.2013.51.6.739. 24516282 10.3347/kjp.2013.51.6.739 PMC3916466

[42] Boonyasiri A, Cheunsuchon P, Suputtamongkol Y, Yamasaki H, Sanpool O, Maleewong W, et al. Nine human sparganosis cases in Thailand with molecular identification of causative parasite species. Am J Trop Med Hyg. 2014;91(2):389–93. 10.4269/ajtmh.14-0178. 24842879 10.4269/ajtmh.14-0178 PMC4125267

[43] Koonmee S, Intapan PM, Yamasaki H, Sugiyama H, Muto M, Kuramochi T, et al. Molecular identification of a causative parasite species using formalin-fixed paraffin embedded (FFPE) tissues of a complicated human pulmonary sparganosis case without decisive clinical diagnosis. Parasitol Int. 2011;60(4):460–4. 10.1016/j.parint.2011.07.018. 21835263 10.1016/j.parint.2011.07.018

[44] Saksirisampant W, Eamudomkarn C, Jeon H-K, Eom KS, Assavapongpaiboon B, Sintuwong S, et al. Ocular sparganosis: the first report of *Spirometra ranarum* in Thailand. Korean J Parasitol. 2020;58(5):577–81. 10.3347/kjp.2020.58.5.577. 33202511 10.3347/kjp.2020.58.5.577 PMC7672231

[45] Omar HM, Fahmy M, Abuowarda M. Hand palm sparganosis: morphologically and genetically confirmed *Spirometra erinaceieuropaei* in a fourteen-year-old girl, Egypt. J Parasit Dis. 2023;47(4):859–64. 10.1007/s12639-023-01623-5. 38009142 10.1007/s12639-023-01623-5 PMC10667186

[46] Eberhard ML, Thiele EA, Yembo GE, Yibi MS, Cama VA, Ruiz-Tiben E. Thirty-seven human cases of sparganosis from Ethiopia and South Sudan caused by *Spirometra *Spp. Am J Trop Med Hyg. 2015;93(2):350–5. 10.4269/ajtmh.15-0236. 26055739 10.4269/ajtmh.15-0236 PMC4530760

[47] Gonzenbach RR, Kong Y, Beck B, Buck A, Weller M, Semmler A. High-dose praziquantel therapy for cerebral sparganosis. J Neurol. 2013;260(5):1423–5. 10.1007/s00415-013-6901-7. 23546305 10.1007/s00415-013-6901-7

[48] Schauer F, Poppert S, Technau-Hafsi K, Häcker G, Jakob T. Subcutaneous infection with *Spirometra* spp. after vacation in South America. J Dtsch Dermatol Ges. 2013;11:150–1. 10.1111/ddg.12063.

[49] Kavana N, Sonaimuthu P, Kasanga C, Kassuku A, Al-Mekhlafi HM, Fong MY, et al. Seroprevalence of sparganosis in rural communities of northern Tanzania. Am J Trop Med Hyg. 2016;95(4):874–6. 10.4269/ajtmh.16-0211. 27481059 10.4269/ajtmh.16-0211 PMC5062792

[50] Park HY, Lee SU, Kim SH, Lee PC, Huh S, Yang YS, et al. Epidemiological significance of sero-positive inhabitants against sparganum in Kangwon-do, Korea. Yonsei Med J. 2001;42(4):371–4. 10.3349/ymj.2001.42.4.371. 11519077 10.3349/ymj.2001.42.4.371

[51] Song P, Li H, Guo J, Ai L, Lu Y, Cai Y, et al. Laboratory detection of parasitic infections in clinical samples from hospitals in Shanghai during 2014–2017. Chin J Parasitol Parasit Dis. 2018;36(5):489–94. (in Chinese)

[52] Chen S, Zhang Y, Li H, Cai Y, Chen J. Analysis on parasitic infection of clinical samples from hospitals in shanghai during 2011–2013. Chin J Parasitol Parasit Dis. 2014;32(6):446–51. (in Chinese) 25902676

[53] Zhang Y, Li J, Deng Y, Chen W, Zhu Y, Lin X. Evaluation of key parasitic diseases control program in Henan Province. Modern Dis Control Prev. 2020;31(11):814–8. 10.13515/j.cnki.hnjpm.1006-8414.2020.11.005. (in Chinese)

[54] Kong Y, Cho SY, Kang WS. Sparganum infections in normal adult population and epileptic patients in Korea: a seroepidemiologic observation. Korean J Parasitol. 1994;32(2):85–92. 10.3347/kjp.1994.32.2.85. 8025037 10.3347/kjp.1994.32.2.85

[55] Zhang Q, Huang D, Zhang R, Deng P, Yang D, Yang X, et al. Survey of infectious status of food -borne parasitic diseases in Shenzhen City. Chin Trop Med. 2011;11(08):951–3. (in Chinese)

[56] Jiang D, Tang Y, Zhang R, Yang F, Wu C, Li Y, et al. Infection status of food-borne parasitic diseases among outpatients in Shenzhen. Electron J Emerg Infect Dis. 2017;2(4):210–3. (in Chinese)

[57] Wang ZQ, Lin XM, Zhang HW, Xu BL, Zhang X, Jiang P, et al. Serological survey for sparganum infection in people of central China. Helminthologia. 2014;51(2):158–61. 10.2478/s11687-014-0223-x.

[58] Zhang Z, Ye Y, Tang Y, Zhang Z, Lin X. Serological analysis of suspected parasitic cases in Shenzhen from 2017 to 2019 years. Electron J Emerg Infect Dis. 2021;6(2):125–8. (in Chinese)

[59] Zhang Y, Jiang T, Ma X, Deng Y, Chen W, Zhu Y, et al. Risk assessment of human *Spirometra mansoni* infections and cross⁃sectional study on knowledge, attitude and practice towards sparganosis in endemic areas of Henan Province. Chin J Schistosomiasis Control. 2025;37(2):190–5. (in Chinese) 10.16250/j.32.1374.2024096 40425502

[60] Wang J, Xu W, Zhu S, Wang H, Tang Y, Wu L. Survey on knowledge, attitudes, practices, and infection status of sparganosis among rural primary school students in Hangzhou. Chin J Sch Health. 2010;31(01):117–8. 10.16835/j.cnki.1000-9817.2010.01.059. (in Chinese)

[61] Lee MK, Hong SJ, Kim HR. Seroprevalence of tissue invading parasitic infections diagnosed by ELISA in Korea. J Korean Med Sci. 2010;25(9):1272–6. 10.3346/jkms.2010.25.9.1272. 20808668 10.3346/jkms.2010.25.9.1272 PMC2923801

[62] Jin Y, Kim EM, Choi MH, Oh MD, Hong ST. Significance of serology by multi-antigen ELISA for tissue helminthiases in Korea. J Korean Med Sci. 2017;32(7):1118–23. 10.3346/jkms.2017.32.7.1118. 28581268 10.3346/jkms.2017.32.7.1118 PMC5461315

[63] Lee KJ, Bae YT, Kim DH, Deung YK, Ryang YS. A seroepidemiologic survey for human sparganosis in Gangweon-do. Korean J Parasitol. 2002;40(4):177–80. 10.3347/kjp.2002.40.4.177. 12509101 10.3347/kjp.2002.40.4.177 PMC2721028

[64] Lee MR, Ju JW, Yang HW, Kim TS, Park MY, Cho SH. Seropositivity and identification of paramyosin for sparganosis in the Kangwon and Incheon Provinces of the Republic of Korea. J Helminthol. 2017;91(5):642–6. 10.1017/S0022149X16000584. 27628641 10.1017/S0022149X16000584

[65] Liu C, Chen Z, Shaban UT, Wang M, Zhou G, Wang N, et al. Misdiagnosis of cerebral sparganosis coexisting with HIV/AIDS: a case report. Int J Infect Dis. 2022;117:264–6. 10.1016/j.ijid.2022.02.016. 35151855 10.1016/j.ijid.2022.02.016

[66] Pan LY, Yang DF. A case of cerebral sparganosis misdiagnosed as intracranial infection. Chin J Parasitol Parasit Dis. 2021;39(05):598–9. (in Chinese)

[67] Yi ZS, Zeng XY, Wu ZQ, Chen H, Chen Y, Zhong JM. A case analysis of misdiagnosis of cerebral sparganosis in a child. Jiangxi Med J. 2024;59(11):1080–2. (in Chinese)

[68] Xu YM, Xie Q, Li SB, Zhuge CD. Analysis of clinical features in 12 patients with sparganosis mansoni. J Diagn Concepts Pract. 2008;03:326–9. 10.16150/j.1671-2870.2008.03.010. (in Chinese)

[69] Hu J, Liao K, Feng X, Jiang D, Liu H, Zheng Q, et al. Surgical treatment of a patient with live intracranial sparganosis for 17 years. BMC Infect Dis. 2022;22(1):353. 10.1186/s12879-022-07293-7. 35397512 10.1186/s12879-022-07293-7 PMC8994396

[70] Dhanoa A, Hassan SS, Jahan NK, Reidpath DD, Fatt QK, Ahmad MP, et al. Seroprevalence of dengue among healthy adults in a rural community in Southern Malaysia: a pilot study. Infect Dis Poverty. 2018;7(1):1. 10.1186/s40249-017-0384-1. 29335021 10.1186/s40249-017-0384-1 PMC5769361

[71] Mahdy MAK, Alareqi LMQ, Abdul-Ghani R, Al-Eryani SMA, Al-Mikhlafy AA, Al-Mekhlafi AM, et al. A community-based survey of *Toxoplasma gondii* infection among pregnant women in rural areas of Taiz governorate, Yemen: the risk of waterborne transmission. Infect Dis Poverty. 2017;6(1):26. 10.1186/s40249-017-0243-0. 28190399 10.1186/s40249-017-0243-0 PMC5304399

[72] Chen LF, Chen WF, Li XJ, Gao YY, Peng BW, Yang SD. Clinical analysis of 9 cases of parasitic encephalopathy in children. J Clin Pediatr. 2019;37(11):827–32. (in Chinese)

[73] Yong TS, Yeo IS, Seo JH, Chang JK, Lee JS, Kim TS, et al. Serodiagnosis of cysticercosis by ELISA-inhibition test using monoclonal antibodies. Korean J Parasitol. 1993;31(2):149. 10.3347/kjp.1993.31.2.149. 8343457 10.3347/kjp.1993.31.2.149

[74] Nishiyama T, Ide T, Himes SR Jr, Ishizaka S, Araki T. Immunodiagnosis of human sparganosis mansoni by micro-chemiluminescence enzyme-linked immunosorbent assay. Trans R Soc Trop Med Hyg. 1994;88(6):663–5. 10.1016/0035-9203(94)90218-6. 7886764 10.1016/0035-9203(94)90218-6

[75] Oh Y, Kim JT, Kim MK, Chang YJ, Eom K, Park JG, et al. Eosinophilic pleuritis due to sparganum: a case report. Korean J Parasitol. 2014;52(5):541–3. 10.3347/kjp.2014.52.5.541. 25352705 10.3347/kjp.2014.52.5.541 PMC4210739

[76] Hong D, H HX, Zhu M, Wan H, Xu R, Wu Y. Cerebral sparganosis in mainland Chinese patients. J Clin Neurosci. 2013;20(11):1514–9. 10.1016/j.jocn.2012.12.018. 23911107 10.1016/j.jocn.2012.12.018

[77] Hong D, Xie H, Wan H, An N, Xu C, Zhang J. Efficacy comparison between long-term high-dose praziquantel and surgical therapy for cerebral sparganosis: a multicenter retrospective cohort study. PLoS Negl Trop Dis. 2018;12(10):e0006918. 10.1371/journal.pntd.0006918. 30346956 10.1371/journal.pntd.0006918 PMC6211769

[78] Zhang P, Zou Y, Yu FX, Wang Z, Lv H, Liu XH, et al. Follow-up study of high-dose praziquantel therapy for cerebral sparganosis. PLoS Negl Trop Dis. 2019;13(1):e0007018. 10.1371/journal.pntd.0007018. 30640909 10.1371/journal.pntd.0007018 PMC6331082

[79] Badri M, Olfatifar M, Karimipoursaryazdi A, Zaki L, Madeira De Carvalho LM, Fasihi Harandi M, et al. The global prevalence of *Spirometra* parasites in snakes, frogs, dogs, and cats: a systematic review and meta‐analysis. Vet Med Sci. 2022;8(6):2785–805. 10.1002/vms3.932. 36084292 10.1002/vms3.932 PMC9677416

[80] Badri M, Eslahi AV, Majidiani H, Pirestani M. *Spirometra erinaceieuropaei* in a wildcat (*Felis silvestris*) in Iran. Vet Parasitol Reg Stud Rep. 2017;10:58–61. 10.1016/j.vprsr.2017.08.004. 10.1016/j.vprsr.2017.08.004 31014600

[81] Alvarado-Hidalgo I, Campos-Camacho J, Arguedas-Morales Y, Romero-Vega LM, Alfaro-Alarcón A, Anchia-Ureña G, et al. Molecular, morphological and histopathological evidence of *Spirometra mansoni* in wild and domestic animals from Costa Rica. Vet Parasitol Reg Stud Rep. 2024;51:101030. 10.1016/j.vprsr.2024.101030. 10.1016/j.vprsr.2024.101030 38772646

[82] Li T, Zhou XN, Tanner M. One Health: enabler of effective prevention, control and elimination of emerging and re-emerging infectious diseases. Infect Dis Poverty. 2025;14(1):77. 10.1186/s40249-025-01337-1. 40745334 10.1186/s40249-025-01337-1 PMC12312372

[83] Laing G, Vigilato MAN, Cleaveland S, Thumbi SM, Blumberg L, Salahuddin N, et al. One health for neglected tropical diseases. Trans R Soc Trop Med Hyg. 2021;115(2):182–4. 10.1093/trstmh/traa117. 33169163 10.1093/trstmh/traa117 PMC7842102

[84] Berger L, Skerratt LF, Zhu XQ, Young S, Speare R. Severe sparganosis in Australian tree frogs. J Wildl GIS. 2009;45(4):921–9. 10.7589/0090-3558-45.4.921. 10.7589/0090-3558-45.4.921 19901368

[85] Trupti B, Shirish N, Maneesha P, Santosh A. An unusual case of urinary sparganosis in the Indian subcontinent. Indian J Urol. 2018;34(2):158–60. 10.4103/iju.IJU_273_17. 29692513 10.4103/iju.IJU_273_17 PMC5894292

[86] Alvarez P, Melgarejo C, Beltran G, Santos R, Ferrer K, Elescano I, et al. Four case reports of cutaneous sparganosis from Peruvian Amazon. Am J Dermatopathol. 2022;44(7):510–4. 10.1097/dad.0000000000002205. 35503880 10.1097/DAD.0000000000002205

[87] Tambo E, Ai L, Zhou X, Chen JH, Hu W, Bergquist R, et al. Surveillance-response systems: the key to elimination of tropical diseases. Infect Dis Poverty. 2014;3(1):17. 10.1186/2049-9957-3-17. 24971165 10.1186/2049-9957-3-17 PMC4071800

[88] Cao J, Sturrock HJW, Cotter C, Zhou S, Zhou H, Liu Y, et al. Communicating and monitoring surveillance and response activities for malaria elimination: China’s “1-3-7” strategy. PLoS Med. 2014;11(5):e1001642. 10.1371/journal.pmed.1001642. 24824170 10.1371/journal.pmed.1001642 PMC4019513

[89] Mari L, Gatto M, Ciddio M, Dia ED, Sokolow SH, De Leo GA, et al. Big-data-driven modeling unveils country-wide drivers of endemic schistosomiasis. Sci Rep. 2017;7(1):489. 10.1038/s41598-017-00493-1. 28352101 10.1038/s41598-017-00493-1 PMC5428445

[90] Brooker S, Clements ACA, Bundy DAP. Global epidemiology, ecology and control of soil-transmitted helminth infections. Adv Parasitol. 2006;62:221–61. 10.1016/s0065-308x(05)62007-6. 16647972 10.1016/S0065-308X(05)62007-6 PMC1976253

