# Supplementary material for: Current status, challenges and control of human sparganosis worldwide: a scoping review
Source: Infect Dis Poverty. 2026 Mar 31;15:38. doi: 10.1186/s40249-026-01434-9 (PMC13037078; doi:10.1186/s40249-026-01434-9)
Supplement: Supplementary file 1 — Additional file 1. [file 40249_2026_1434_MOESM1_ESM.docx]

**Literature search strategy**

CNKI: (Title, Abstract, Keywords: lietouyou (exact)) OR (Title, Abstract, Keywords: diegongshu (exact)) OR (Title, Abstract, Keywords: diegongtaochong (exact))

WanFang: Topic:("lietouyou") OR Topic:("diegongshu") OR Topic:("diegongtaochong")

PubMed: ((sparganosis[Title/Abstract]) OR (spirometra[Title/Abstract])) OR (plerocercoid[Title/Abstract])

Web of Science: sparganosis (Topic) OR spirometra (Topic) OR plerocercoid (Topic)

Scopus: TITLE-ABS-KEY(sparganosis OR spirometra OR plerocercoid)

Embase: sparganosis: ab, ti OR spirometra: ab, ti OR plerocercoid: ab, ti
