# Supplementary material for: Current status, challenges and control of human sparganosis worldwide: a scoping review
Source: Infect Dis Poverty. 2026 Mar 31;15:38. doi: 10.1186/s40249-026-01434-9 (PMC13037078; doi:10.1186/s40249-026-01434-9)
Supplement: Supplementary file 2 — Additional file 2. [file 40249_2026_1434_MOESM2_ESM.docx]

# Quality Assessment Report of Articles Included in the Review

The quality of the included articles was assessed using the Joanna Briggs Institute (JBI) Prevalence Critical Appraisal Tool. The tool assessed each article on the following:

1. Sample representative of the target population
2. Study participants recruited in an appropriate way
3. Sample size adequate
4. Study subjects and setting described in detail
5. Data analysis was conducted with enough coverage of the identified sample
6. Validity of methods used to identify the condition of interest
7. Condition measured in a standard, reliable way for all participants
8. Appropriate statistical analysis
9. Adequacy of response rate; were low response rates managed?
10. Sub-population of interest identified using objective criteria

**Table** The Joanna Briggs Institute (JBI) Prevalence Critical Appraisal Tool

| **Author(s) and Year** | **Was the sample representative of the target population?** | **Were study participants recruited in an appropriate way?** | **Was the sample size adequate?** | **Were the study subjects and setting described in detail?** | **Was data analysis conducted with sufficient coverage of the identified sample?** | **Were valid methods used for the identification of the condition?** | **Was the condition measured in a standard, reliable way for all participants?** | **Was there appropriate statistical analysis?** | **Was the response rate adequate, and if not, was the low response rate managed appropriately?** | **Were target subpopulations identified using objective criteria?** | **Quality score** |
| --- | --- | --- | --- | --- | --- | --- | --- | --- | --- | --- | --- |
| Chen XY et al., 2022 | Yes | Yes | Yes | Yes | Not specified | Yes | Yes | NA | NA | Not specified | 6 |
| Xie X et al., 2018 | Yes | Yes | Yes | Yes | Not specified | Yes | Yes | NA | NA | Not specified | 6 |
| Li H et al., 2015 | Yes | Yes | Yes | Yes | Not specified | Yes | Yes | NA | NA | Not specified | 6 |
| Li H et al., 2019 | Yes | Yes | Yes | Yes | Not specified | Yes | Yes | NA | NA | Not specified | 6 |
| Ho T H et al., 2013 | Yes | Yes | Yes | Yes | Not specified | Yes | Yes | NA | NA | Not specified | 6 |
| Li QY et al., 2011 | Yes | Yes | Yes | Yes | Not specified | Yes | Yes | NA | NA | Not specified | 6 |
| Lou YJ et al., 2013 | Yes | Yes | Yes | Yes | Not specified | Yes | Yes | NA | NA | Not specified | 6 |
| Cai Y et al., 2009 | Yes | Yes | Yes | Yes | Not specified | Yes | Yes | NA | NA | Not specified | 6 |
| Zhu SJ et al., 2008 | Yes | Yes | Yes | Yes | Not specified | Yes | Yes | NA | NA | Not specified | 6 |
| Li YR et al., 2008 | Yes | Yes | Yes | Yes | Not specified | Yes | Yes | NA | NA | Not specified | 6 |
| Chen BJ et al., 2007 | Yes | Yes | Yes | Yes | Not specified | Yes | Yes | NA | NA | Not specified | 6 |
| Liang F, 2012 | Yes | Yes | Yes | Yes | Not specified | Yes | Yes | NA | NA | Not specified | 6 |
| Wu LM et al., 2007 | Yes | Yes | Yes | Yes | Not specified | Yes | Yes | NA | NA | Not specified | 6 |
| Peng T et al., 2006 | Yes | Yes | Yes | Yes | Not specified | Yes | Yes | NA | NA | Not specified | 6 |
| Zhang J et al., 2005 | Yes | Yes | Yes | Yes | Not specified | Yes | Yes | NA | NA | Not specified | 6 |
| Shao YN et al., 2004 | Yes | Yes | Yes | Yes | Not specified | Yes | Yes | NA | NA | Not specified | 6 |
| Li LS et al., 2009 | Yes | Yes | Yes | Yes | Not specified | Yes | Yes | NA | NA | Not specified | 6 |
| Wang XC, 2003 | Yes | Yes | Yes | Yes | Not specified | Yes | Yes | NA | NA | Not specified | 6 |
| Xiu HW et al., 2003 | Yes | Yes | Yes | Yes | Not specified | Yes | Yes | NA | NA | Not specified | 6 |
| Lin JX et al., 2002 | Yes | Yes | Yes | Yes | Not specified | Yes | Yes | NA | NA | Not specified | 6 |
| Gui YJ, 2001 | Yes | Yes | Yes | Yes | Not specified | Yes | Yes | NA | NA | Not specified | 6 |
| Li JS, 2003 | Yes | Yes | Yes | Yes | Not specified | Yes | Yes | NA | NA | Not specified | 6 |
| Du YZ, 2002 | Yes | Yes | Yes | Yes | Not specified | Yes | Yes | NA | NA | Not specified | 6 |
| Zhou P et al., 2001 | Yes | Yes | Yes | Yes | Not specified | Yes | Yes | NA | NA | Not specified | 6 |
| Zhou H et al., 1997 | Yes | Yes | Yes | Yes | Not specified | Yes | Yes | NA | NA | Not specified | 6 |
| Ye J, 1997 | Yes | Yes | Yes | Yes | Not specified | Yes | Yes | NA | NA | Not specified | 6 |
| Qu WY, 1997 | Yes | Yes | Yes | Yes | Not specified | Yes | Yes | NA | NA | Not specified | 6 |
| Xie HP et al., 1996 | Yes | Yes | Yes | Yes | Not specified | Yes | Yes | NA | NA | Not specified | 6 |
| Dai JH et al., 1998 | Yes | Yes | Yes | Yes | Not specified | Yes | Yes | NA | NA | Not specified | 6 |
| Wu XH et al., 1991 | Yes | Yes | Yes | Yes | Not specified | Yes | Yes | NA | NA | Not specified | 6 |
| Li KX, 1992 | Yes | Yes | Yes | Yes | Not specified | Yes | Yes | NA | NA | Not specified | 6 |
| Ding YQ, 1995 | Yes | Yes | Yes | Yes | Not specified | Yes | Yes | NA | NA | Not specified | 6 |
| Li YZ et al., 1988 | Yes | Yes | Yes | Yes | Not specified | Yes | Yes | NA | NA | Not specified | 6 |
| Shen SY, 1989 | Yes | Yes | Yes | Yes | Not specified | Yes | Yes | NA | NA | Not specified | 6 |
| Zheng XH, 1989 | Yes | Yes | Yes | Yes | Not specified | Yes | Yes | NA | NA | Not specified | 6 |
| Mo JG, 1985 | Yes | Yes | Yes | Yes | Not specified | Yes | Yes | NA | NA | Not specified | 6 |
| Wen CF, 1985 | Yes | Yes | Yes | Yes | Not specified | Yes | Yes | NA | NA | Not specified | 6 |
| Xie NP et al., 1983 | Yes | Yes | Yes | Yes | Not specified | Yes | Yes | NA | NA | Not specified | 6 |
| Chen FC, 1985 | Yes | Yes | Yes | Yes | Not specified | Yes | Yes | NA | NA | Not specified | 6 |
| Liu GC et al., 1981 | Yes | Yes | Yes | Yes | Not specified | Yes | Yes | NA | NA | Not specified | 6 |
| Qiu YN et al., 1984 | Yes | Yes | Yes | Yes | Not specified | Yes | Yes | NA | NA | Not specified | 6 |
| Li Y et al., 1981 | Yes | Yes | Yes | Yes | Not specified | Yes | Yes | NA | NA | Not specified | 6 |
| Xu JG, 1980 | Yes | Yes | Yes | Yes | Not specified | Yes | Yes | NA | NA | Not specified | 6 |
| Ye JC, 1995 | Yes | Yes | Yes | Yes | Not specified | Yes | Yes | NA | NA | Not specified | 6 |
| Zhao ZP et al., 1979 | Yes | Yes | Yes | Yes | Not specified | Yes | Yes | NA | NA | Not specified | 6 |
| Xu H, 1992 | Yes | Yes | Yes | Yes | Not specified | Yes | Yes | NA | NA | Not specified | 6 |
| Chen CX, 1981 | Yes | Yes | Yes | Yes | Not specified | Yes | Yes | NA | NA | Not specified | 6 |
| Niu D, 1984 | Yes | Yes | Yes | Yes | Not specified | Yes | Yes | NA | NA | Not specified | 6 |
| Zhen KT, 1981 | Yes | Yes | Yes | Yes | Not specified | Yes | Yes | NA | NA | Not specified | 6 |
| Xia Q et al., 2019. | Yes | Yes | Yes | Yes | Not specified | Yes | Yes | NA | NA | Not specified | 6 |
| Li XL et al., 2014 | Yes | Yes | Yes | Yes | Not specified | Yes | Yes | NA | NA | Not specified | 6 |
| Ye H et al., 2012 | Yes | Yes | Yes | Yes | Not specified | Yes | Yes | NA | NA | Not specified | 6 |
| Liu GY, 2006 | Yes | Yes | Yes | No | Not specified | Yes | Yes | NA | NA | Not specified | 5 |
| Zheng JP, 2003 | Yes | Yes | Yes | Yes | Not specified | Yes | Yes | NA | NA | Not specified | 6 |
| Wang XH et al., 2001 | Yes | Yes | Yes | Yes | Not specified | Yes | Yes | NA | NA | Not specified | 6 |
| Li JF et al., 2001 | Yes | Yes | Yes | Yes | Not specified | Yes | Yes | NA | NA | Not specified | 6 |
| Zhou H et al., 1994 | Yes | Yes | Yes | Yes | Not specified | Yes | Yes | NA | NA | Not specified | 6 |
| Tang SX et al., 1991 | Yes | Yes | Yes | Yes | Not specified | Yes | Yes | NA | NA | Not specified | 6 |
| Zhang RL et al., 1990 | Yes | Yes | Yes | Yes | Not specified | Yes | Yes | NA | NA | Not specified | 6 |
| Pang YJ et al., 1987 | Yes | Yes | Yes | Yes | Not specified | Yes | Yes | NA | NA | Not specified | 6 |
| Wang KZ et al., 1986 | Yes | Yes | Yes | Yes | Not specified | Yes | Yes | NA | NA | Not specified | 6 |
| Hong WX, 1985 | Yes | Yes | Yes | Yes | Not specified | Yes | Yes | NA | NA | Not specified | 6 |
| Huang HB, 1984 | Yes | Yes | Yes | Yes | Not specified | Yes | Yes | NA | NA | Not specified | 6 |
| Gong XM, 1981 | Yes | Yes | Yes | Yes | Not specified | Yes | Yes | NA | NA | Not specified | 6 |
| Wang CY et al., 1979 | Yes | Yes | Yes | Yes | Not specified | Yes | Yes | NA | NA | Not specified | 6 |
| Deng XY et al., 1979 | Yes | Yes | Yes | Yes | Not specified | Yes | Yes | NA | NA | Not specified | 6 |
| Guangxi Health,1974(06):31-32 | Yes | Yes | Yes | Yes | Not specified | Yes | Yes | NA | NA | Not specified | 6 |
| Zhao YM et al., 1965 | Yes | Yes | Yes | Yes | Not specified | Yes | Yes | NA | NA | Not specified | 6 |
| Huang YQ et al., 2019 | Yes | Yes | Yes | No | Not specified | Yes | Yes | NA | NA | Not specified | 5 |
| Liu SZ et al., 1994 | Yes | Yes | Yes | No | Not specified | Yes | Yes | NA | NA | Not specified | 5 |
| Lin FS et al., 1991 | Yes | Yes | Yes | Yes | Not specified | Yes | Yes | NA | NA | Not specified | 6 |
| Song ZY, 1988 | Yes | Yes | Yes | Yes | Not specified | Yes | Yes | NA | NA | Not specified | 6 |
| Deng ZF et al., 1984 | Yes | Yes | Yes | No | Not specified | Yes | Yes | NA | NA | Not specified | 5 |
| Tang Y et al., 1984 | Yes | Yes | Yes | Yes | Not specified | Yes | Yes | NA | NA | Not specified | 6 |
| Chen YY et al., 1992 | Yes | Yes | Yes | Yes | Not specified | Yes | Yes | NA | NA | Not specified | 6 |
| Li GS, 1987 | Yes | Yes | Yes | Yes | Not specified | Yes | Yes | NA | NA | Not specified | 6 |
| Zhong YX, 1983 | Yes | Yes | Yes | Yes | Not specified | Yes | Yes | NA | NA | Not specified | 6 |
| Xue BJ, 1984 | Yes | Yes | Yes | Yes | Not specified | Yes | Yes | NA | NA | Not specified | 6 |
| Shi YL, 1978 | Yes | Yes | Yes | Yes | Not specified | Yes | Yes | NA | NA | Not specified | 6 |
| Zhang XF, 2011 | Yes | Yes | Yes | Yes | Not specified | Yes | Yes | NA | NA | Not specified | 6 |
| Chen BD et al., 1966 | Yes | Yes | Yes | Yes | Not specified | Yes | Yes | NA | NA | Not specified | 6 |
| Jiang HW et al., 2023 | Yes | Yes | Yes | Yes | Not specified | Yes | Yes | NA | NA | Not specified | 6 |
| Meng H et al., 2019 | Yes | Yes | Yes | Yes | Not specified | Yes | Yes | NA | NA | Not specified | 6 |
| Zhong HL et al., 1983 | Yes | Yes | Yes | No | Not specified | Yes | Yes | NA | NA | Not specified | 5 |
| Cho D et al., 2024 | Yes | Yes | Yes | Yes | Not specified | Yes | Yes | NA | NA | Not specified | 6 |
| Yang JW et al., 2007 | Yes | Yes | Yes | Yes | Not specified | Yes | Yes | NA | NA | Not specified | 6 |
| Yoon KC et al., 2004 | Yes | Yes | Yes | Yes | Not specified | Yes | Yes | NA | NA | Not specified | 6 |
| 최은수 et al., 2005 | Yes | Yes | Yes | Yes | Not specified | Yes | Yes | NA | NA | Not specified | 6 |
| Kubota T et al., 2007 | Yes | Yes | Yes | Yes | Not specified | Yes | Yes | NA | NA | Not specified | 6 |
| Saksirisampant W et al., 2020 | Yes | Yes | Yes | Yes | Not specified | Yes | Yes | NA | NA | Not specified | 6 |
| Preechawat P. et al., 2011 | Yes | Yes | Yes | Yes | Not specified | Yes | Yes | NA | NA | Not specified | 6 |
| Nath R et al., 2015 | Yes | Yes | Yes | Yes | Not specified | Yes | Yes | NA | NA | Not specified | 6 |
| Subudhi BN et al., 2006 | Yes | Yes | Yes | Yes | Not specified | Yes | Yes | NA | NA | Not specified | 6 |
| Botterel F et al., 2003 | Yes | Yes | Yes | Yes | Not specified | Yes | Yes | NA | NA | Not specified | 6 |
| Beltrán Fabián M et al., 2015 | Yes | Yes | Yes | Yes | Not specified | Yes | Yes | NA | NA | Not specified | 6 |
| Mentz MB et al., 2011 | Yes | Yes | Yes | Yes | Not specified | Yes | Yes | NA | NA | Not specified | 6 |
| Sah R et al., 2020 | Yes | Yes | Yes | Yes | Not specified | Yes | Yes | NA | NA | Not specified | 6 |
| De Meulder et al., 2017 | Yes | Yes | Yes | No | Not specified | Yes | Yes | NA | NA | Not specified | 5 |
| Rehák M et al., 2006 | Yes | Yes | Yes | Yes | Not specified | Yes | Yes | NA | NA | Not specified | 6 |
| Jones DW et al., 1962 | Yes | Yes | Yes | No | Not specified | Yes | Yes | NA | NA | Not specified | 5 |
| Leon LA et al., 1972 | Yes | Yes | Yes | Yes | Not specified | Yes | Yes | NA | NA | Not specified | 6 |
| BOTERO D et al., 1958 | Yes | Yes | Yes | Yes | Not specified | Yes | Yes | NA | NA | Not specified | 6 |
| Mougeot G et al., 1999 | Yes | Yes | Yes | No | Not specified | Yes | Yes | NA | NA | Not specified | 5 |
| Fang TF et al., 2012 | Yes | Yes | Yes | Yes | Not specified | Yes | Yes | NA | NA | Not specified | 6 |
| Long X et al., 2008 | Yes | Yes | Yes | Yes | Not specified | Yes | Yes | NA | NA | Not specified | 6 |
| He ZJ et al., 2008 | Yes | Yes | Yes | Yes | Not specified | Yes | Yes | NA | NA | Not specified | 6 |
| Wang KX et al., 2005 | Yes | Yes | Yes | Yes | Not specified | Yes | Yes | NA | NA | Not specified | 6 |
| Deng ZG, 2000 | Yes | Yes | Yes | Yes | Not specified | Yes | Yes | NA | NA | Not specified | 6 |
| Zheng YF, 1999 | Yes | Yes | Yes | Yes | Not specified | Yes | Yes | NA | NA | Not specified | 6 |
| Li SY et al., 2001 | Yes | Yes | Yes | Yes | Not specified | Yes | Yes | NA | NA | Not specified | 6 |
| Dai CL et al., 1996 | Yes | Yes | Yes | Yes | Not specified | Yes | Yes | NA | NA | Not specified | 6 |
| Jing XD et al., 1995 | Yes | Yes | Yes | Yes | Not specified | Yes | Yes | NA | NA | Not specified | 6 |
| Fang XH et al., 1991 | Yes | Yes | Yes | Yes | Not specified | Yes | Yes | NA | NA | Not specified | 6 |
| Yu WB et al., 1989 | Yes | Yes | Yes | Yes | Not specified | Yes | Yes | NA | NA | Not specified | 6 |
| Xu GC et al., 1986 | Yes | Yes | Yes | Yes | Not specified | Yes | Yes | NA | NA | Not specified | 6 |
| Mao DS et al., 1985 | Yes | Yes | Yes | Yes | Not specified | Yes | Yes | NA | NA | Not specified | 6 |
| Xu BL, 1983 | Yes | Yes | Yes | Yes | Not specified | Yes | Yes | NA | NA | Not specified | 6 |
| Ji JB, 1992 | Yes | Yes | Yes | Yes | Not specified | Yes | Yes | NA | NA | Not specified | 6 |
| Chen GY, 1979 | Yes | Yes | Yes | No | Not specified | Yes | Yes | NA | NA | Not specified | 5 |
| Chongqing Medicine,1978(01):52-56 | Yes | Yes | Yes | Yes | Not specified | Yes | Yes | NA | NA | Not specified | 6 |
| Li QY, 2001 | Yes | Yes | Yes | Yes | Not specified | Yes | Yes | NA | NA | Not specified | 6 |
| Zhong ZM, 1979 | Yes | Yes | Yes | Yes | Not specified | Yes | Yes | NA | NA | Not specified | 6 |
| Chen HM et al., 1979 | Yes | Yes | Yes | Yes | Not specified | Yes | Yes | NA | NA | Not specified | 6 |
| Huang LH et al., 2013 | Yes | Yes | Yes | Yes | Not specified | Yes | Yes | NA | NA | Not specified | 6 |
| Chen sh et al., 1998 | Yes | Yes | Yes | Yes | Not specified | Yes | Yes | NA | NA | Not specified | 6 |
| Chen RY, 1991 | Yes | Yes | Yes | Yes | Not specified | Yes | Yes | NA | NA | Not specified | 6 |
| Zhang GH, 1990 | Yes | Yes | Yes | Yes | Not specified | Yes | Yes | NA | NA | Not specified | 6 |
| Liang ZM et al., 1979 | Yes | Yes | Yes | Yes | Not specified | Yes | Yes | NA | NA | Not specified | 6 |
| Li SP et al., 1995 | Yes | Yes | Yes | Yes | Not specified | Yes | Yes | NA | NA | Not specified | 6 |
| Li XD et al., 2010 | Yes | Yes | Yes | Yes | Not specified | Yes | Yes | NA | NA | Not specified | 6 |
| Zhao K et al., 2008 | Yes | Yes | Yes | Yes | Not specified | Yes | Yes | NA | NA | Not specified | 6 |
| Lee et al., 2010 | Yes | Yes | Yes | Yes | Not specified | Yes | Yes | NA | NA | Not specified | 6 |
| Iamaroon A et al., 2002 | Yes | Yes | Yes | Yes | Not specified | Yes | Yes | NA | NA | Not specified | 6 |
| Rywlin AM et al., 1968 | Yes | Yes | Yes | Yes | Not specified | Yes | Yes | NA | NA | Not specified | 6 |
| Ping Yang et al., 2022 | Yes | Yes | Yes | Yes | Not specified | Yes | Yes | NA | NA | Not specified | 6 |
| Fu XY et al., 2023 | Yes | Yes | Yes | Yes | Not specified | Yes | Yes | NA | NA | Not specified | 6 |
| Yuepeng Zhang et al., 2021 | Yes | Yes | Yes | Yes | Not specified | Yes | Yes | NA | NA | Not specified | 6 |
| Li ZY et al., 2020 | Yes | Yes | Yes | Yes | Not specified | Yes | Yes | NA | NA | Not specified | 6 |
| Liu GD et al., 2021 | Yes | Yes | Yes | Yes | Not specified | Yes | Yes | NA | NA | Not specified | 6 |
| Shi Q et al., 2019 | Yes | Yes | Yes | Yes | Not specified | Yes | Yes | NA | NA | Not specified | 6 |
| Zhou M et al., 2020 | Yes | Yes | Yes | Yes | Not specified | Yes | Yes | NA | NA | Not specified | 6 |
| Jiang SF et al., 2018 | Yes | Yes | Yes | Yes | Not specified | Yes | Yes | NA | NA | Not specified | 6 |
| Zuo Jun et al., 2019 | Yes | Yes | Yes | Yes | Not specified | Yes | Yes | NA | NA | Not specified | 6 |
| Nathavitharana Ruvandhi R et al., 2015 | Yes | Yes | Yes | Yes | Not specified | Yes | Yes | NA | NA | Not specified | 6 |
| Guo WL et al., 2017 | Yes | Yes | Yes | Yes | Not specified | Yes | Yes | NA | NA | Not specified | 6 |
| Wang N et al., 2016 | Yes | Yes | Yes | Yes | Not specified | Yes | Yes | NA | NA | Not specified | 6 |
| Hu K et al., 2014 | Yes | Yes | Yes | Yes | Not specified | Yes | Yes | NA | NA | Not specified | 6 |
| Jin YS et al., 2012 | Yes | Yes | Yes | Yes | Not specified | Yes | Yes | NA | NA | Not specified | 6 |
| Tan Y et al., 2012 | Yes | Yes | Yes | Yes | Not specified | Yes | Yes | NA | NA | Not specified | 6 |
| Tao CY et al., 2012 | Yes | Yes | Yes | Yes | Not specified | Yes | Yes | NA | NA | Not specified | 6 |
| Chen Y, 2011 | Yes | Yes | Yes | Yes | Not specified | Yes | Yes | NA | NA | Not specified | 6 |
| Wang Q et al., 2011 | Yes | Yes | Yes | Yes | Not specified | Yes | Yes | NA | NA | Not specified | 6 |
| Chen CX, 2011 | Yes | Yes | Yes | Yes | Not specified | Yes | Yes | NA | NA | Not specified | 6 |
| Tian H et al., 2011 | Yes | Yes | Yes | Yes | Not specified | Yes | Yes | NA | NA | Not specified | 6 |
| Zhang YN et al., 2011 | Yes | Yes | Yes | Yes | Not specified | Yes | Yes | NA | NA | Not specified | 6 |
| Tian JQ et al., 2009 | Yes | Yes | Yes | Yes | Not specified | Yes | Yes | NA | NA | Not specified | 6 |
| Zhang KJ et al., 2009 | Yes | Yes | Yes | Yes | Not specified | Yes | Yes | NA | NA | Not specified | 6 |
| A B W Chan et al., 2004 | Yes | Yes | Yes | Yes | Not specified | Yes | Yes | NA | NA | Not specified | 6 |
| Qiu XL et al., 2005 | Yes | Yes | Yes | Yes | Not specified | Yes | Yes | NA | NA | Not specified | 6 |
| Tung C C et al., 2005 | Yes | Yes | Yes | Yes | Not specified | Yes | Yes | NA | NA | Not specified | 6 |
| Teng HF et al., 2000 | Yes | Yes | Yes | Yes | Not specified | Yes | Yes | NA | NA | Not specified | 6 |
| Yang CY, 1995 | Yes | Yes | Yes | Yes | Not specified | Yes | Yes | NA | NA | Not specified | 6 |
| Yang KQ et al., 1993 | Yes | Yes | Yes | Yes | Not specified | Yes | Yes | NA | NA | Not specified | 6 |
| Jiang J et al., 1991 | Yes | Yes | Yes | Yes | Not specified | Yes | Yes | NA | NA | Not specified | 6 |
| Zhou KH et al., 1988 | Yes | Yes | Yes | Yes | Not specified | Yes | Yes | NA | NA | Not specified | 6 |
| Feng SS, 1989 | Yes | Yes | Yes | Yes | Not specified | Yes | Yes | NA | NA | Not specified | 6 |
| Du JG et al., 1988 | Yes | Yes | Yes | Yes | Not specified | Yes | Yes | NA | NA | Not specified | 6 |
| Chen BM et al., 1985 | Yes | Yes | Yes | Yes | Not specified | Yes | Yes | NA | NA | Not specified | 6 |
| Ni SL et al., 1985 | Yes | Yes | Yes | Yes | Not specified | Yes | Yes | NA | NA | Not specified | 6 |
| Song Y et al., 2020 | Yes | Yes | Yes | Yes | Not specified | Yes | Yes | NA | NA | Not specified | 6 |
| Wang JS et al., 1980 | Yes | Yes | Yes | Yes | Not specified | Yes | Yes | NA | NA | Not specified | 6 |
| Wu D et al., 2021 | Yes | Yes | Yes | Yes | Not specified | Yes | Yes | NA | NA | Not specified | 6 |
| Chan AB et al., 2004 | Yes | Yes | Yes | Yes | Not specified | Yes | Yes | NA | NA | Not specified | 6 |
| Tung CC et al., 2005 | Yes | Yes | Yes | Yes | Not specified | Yes | Yes | NA | NA | Not specified | 6 |
| Xin Huang et al., 2023 | Yes | Yes | Yes | Yes | Not specified | Yes | Yes | NA | NA | Not specified | 6 |
| Yan TK et al., 2021 | Yes | Yes | Yes | Yes | Not specified | Yes | Yes | NA | NA | Not specified | 6 |
| Wu DY . et al., 2021 | Yes | Yes | Yes | Yes | Not specified | Yes | Yes | NA | NA | Not specified | 6 |
| Ling P et al., 2022 | Yes | Yes | Yes | Yes | Not specified | Yes | Yes | NA | NA | Not specified | 6 |
| Weng PL et al., 2020 | Yes | Yes | Yes | Yes | Not specified | Yes | Yes | NA | NA | Not specified | 6 |
| Qin LP et al., 2019 | Yes | Yes | Yes | Yes | Not specified | Yes | Yes | NA | NA | Not specified | 6 |
| Diefei Hu et al., 2023 | Yes | Yes | Yes | Yes | Not specified | Yes | Yes | NA | NA | Not specified | 6 |
| Guan ZH et al., 2021 | Yes | Yes | Yes | Yes | Not specified | Yes | Yes | NA | NA | Not specified | 6 |
| Ding HF et al., 2018 | Yes | Yes | Yes | Yes | Not specified | Yes | Yes | NA | NA | Not specified | 6 |
| Yuan Qianqian et al., 2019 | Yes | Yes | Yes | Yes | Not specified | Yes | Yes | NA | NA | Not specified | 6 |
| Liu DX et al., 2018 | Yes | Yes | Yes | Yes | Not specified | Yes | Yes | NA | NA | Not specified | 6 |
| Bailu Du et al., 2018. | Yes | Yes | Yes | Yes | Not specified | Yes | Yes | NA | NA | Not specified | 6 |
| Ma L et al., 2017 | Yes | Yes | Yes | Yes | Not specified | Yes | Yes | NA | NA | Not specified | 6 |
| Xiao B et al., 2019 | Yes | Yes | Yes | Yes | Not specified | Yes | Yes | NA | NA | Not specified | 6 |
| Duan Y et al., 2016 | Yes | Yes | Yes | Yes | Not specified | Yes | Yes | NA | NA | Not specified | 6 |
| Zhong TT et al., 2016 | Yes | Yes | Yes | Yes | Not specified | Yes | Yes | NA | NA | Not specified | 6 |
| Shen MQ et al., 2015 | Yes | Yes | Yes | Yes | Not specified | Yes | Yes | NA | NA | Not specified | 6 |
| Hao H et al. et al., 2014 | Yes | Yes | Yes | Yes | Not specified | Yes | Yes | NA | NA | Not specified | 6 |
| Dong AS et al., 2014 | Yes | Yes | Yes | Yes | Not specified | Yes | Yes | NA | NA | Not specified | 6 |
| Liu D et al. et al., 2016 | Yes | Yes | Yes | Yes | Not specified | Yes | Yes | NA | NA | Not specified | 6 |
| Xu Z, 2013 | Yes | Yes | Yes | Yes | Not specified | Yes | Yes | NA | NA | Not specified | 6 |
| Lan ZH et al., 2013 | Yes | Yes | Yes | Yes | Not specified | Yes | Yes | NA | NA | Not specified | 6 |
| Chen RM, 2014 | Yes | Yes | Yes | Yes | Not specified | Yes | Yes | NA | NA | Not specified | 6 |
| Zhang YM et al., 2013 | Yes | Yes | Yes | Yes | Not specified | Yes | Yes | NA | NA | Not specified | 6 |
| Xu YL et al., 2012 | Yes | Yes | Yes | Yes | Not specified | Yes | Yes | NA | NA | Not specified | 6 |
| Cheng YL et al., 2018 | Yes | Yes | Yes | Yes | Not specified | Yes | Yes | NA | NA | Not specified | 6 |
| Liu ZF et al., 2011 | Yes | Yes | Yes | Yes | Not specified | Yes | Yes | NA | NA | Not specified | 6 |
| Yu WL et al., 2011 | Yes | Yes | Yes | Yes | Not specified | Yes | Yes | NA | NA | Not specified | 6 |
| Wang LM, 2012 | Yes | Yes | Yes | Yes | Not specified | Yes | Yes | NA | NA | Not specified | 6 |
| Zhang J et al., 2011 | Yes | Yes | Yes | Yes | Not specified | Yes | Yes | NA | NA | Not specified | 6 |
| Qi JJ et al., 2011 | Yes | Yes | Yes | Yes | Not specified | Yes | Yes | NA | NA | Not specified | 6 |
| Wang DJ et al., 2011 | Yes | Yes | Yes | Yes | Not specified | Yes | Yes | NA | NA | Not specified | 6 |
| Zhang L, 2012 | Yes | Yes | Yes | Yes | Not specified | Yes | Yes | NA | NA | Not specified | 6 |
| Wu W et al., 2009 | Yes | Yes | Yes | Yes | Not specified | Yes | Yes | NA | NA | Not specified | 6 |
| Li CH, 2009 | Yes | Yes | Yes | Yes | Not specified | Yes | Yes | NA | NA | Not specified | 6 |
| Shi CB, 2009 | Yes | Yes | Yes | Yes | Not specified | Yes | Yes | NA | NA | Not specified | 6 |
| Fu LM et al., 2009 | Yes | Yes | Yes | Yes | Not specified | Yes | Yes | NA | NA | Not specified | 6 |
| Yu MY et al., 2010 | Yes | Yes | Yes | Yes | Not specified | Yes | Yes | NA | NA | Not specified | 6 |
| Xu YH et al., 2009 | Yes | Yes | Yes | Yes | Not specified | Yes | Yes | NA | NA | Not specified | 6 |
| Li JA et al., 2008 | Yes | Yes | Yes | Yes | Not specified | Yes | Yes | NA | NA | Not specified | 6 |
| Wu HB et al., 2008 | Yes | Yes | Yes | Yes | Not specified | Yes | Yes | NA | NA | Not specified | 6 |
| Zhang YS et al., 2008 | Yes | Yes | Yes | Yes | Not specified | Yes | Yes | NA | NA | Not specified | 6 |
| Deng RJ et al., 2007 | Yes | Yes | Yes | Yes | Not specified | Yes | Yes | NA | NA | Not specified | 6 |
| Pan F et al., 2007 | Yes | Yes | Yes | Yes | Not specified | Yes | Yes | NA | NA | Not specified | 6 |
| Yang LJ et al., 2007 | Yes | Yes | Yes | Yes | Not specified | Yes | Yes | NA | NA | Not specified | 6 |
| Jiang YR, 2007 | Yes | Yes | Yes | Yes | Not specified | Yes | Yes | NA | NA | Not specified | 6 |
| Wang JX et al., 2006 | Yes | Yes | Yes | Yes | Not specified | Yes | Yes | NA | NA | Not specified | 6 |
| Popular Science News,2008-01-31(A06) | Yes | Yes | Yes | Yes | Not specified | Yes | Yes | NA | NA | Not specified | 6 |
| Wang JX, 2006 | Yes | Yes | Yes | Yes | Not specified | Yes | Yes | NA | NA | Not specified | 6 |
| Zhang YN et al., 2006 | Yes | Yes | Yes | Yes | Not specified | Yes | Yes | NA | NA | Not specified | 6 |
| Yi D et al., 2005 | Yes | Yes | Yes | Yes | Not specified | Yes | Yes | NA | NA | Not specified | 6 |
| Chen JY et al., 2005 | Yes | Yes | Yes | Yes | Not specified | Yes | Yes | NA | NA | Not specified | 6 |
| Wang CQ et al., 2005 | Yes | Yes | Yes | Yes | Not specified | Yes | Yes | NA | NA | Not specified | 6 |
| Wang DP et al., 2004 | Yes | Yes | Yes | Yes | Not specified | Yes | Yes | NA | NA | Not specified | 6 |
| Wang Q et al., 2004 | Yes | Yes | Yes | Yes | Not specified | Yes | Yes | NA | NA | Not specified | 6 |
| Zhuo R et al., 2007 | Yes | Yes | Yes | Yes | Not specified | Yes | Yes | NA | NA | Not specified | 6 |
| Ouyang HY et al., 2003 | Yes | Yes | Yes | Yes | Not specified | Yes | Yes | NA | NA | Not specified | 6 |
| Zhang LY et al., 2003 | Yes | Yes | Yes | Yes | Not specified | Yes | Yes | NA | NA | Not specified | 6 |
| Cao HX et al., 2003 | Yes | Yes | Yes | Yes | Not specified | Yes | Yes | NA | NA | Not specified | 6 |
| Peng JY et al., 2002 | Yes | Yes | Yes | Yes | Not specified | Yes | Yes | NA | NA | Not specified | 6 |
| Li SH et al., 2003 | Yes | Yes | Yes | Yes | Not specified | Yes | Yes | NA | NA | Not specified | 6 |
| Zhou BJ et al., 2005 | Yes | Yes | Yes | Yes | Not specified | Yes | Yes | NA | NA | Not specified | 6 |
| Ouyang LM et al., 2001 | Yes | Yes | Yes | Yes | Not specified | Yes | Yes | NA | NA | Not specified | 6 |
| Zhang RY et al., 2000 | Yes | Yes | Yes | Yes | Not specified | Yes | Yes | NA | NA | Not specified | 6 |
| Qiao HC et al., 1999 | Yes | Yes | Yes | Yes | Not specified | Yes | Yes | NA | NA | Not specified | 6 |
| Huang XL et al., 1999 | Yes | Yes | Yes | Yes | Not specified | Yes | Yes | NA | NA | Not specified | 6 |
| Lin Z et al., 2001 | Yes | Yes | Yes | Yes | Not specified | Yes | Yes | NA | NA | Not specified | 6 |
| Chang HM et al., 1999 | Yes | Yes | Yes | Yes | Not specified | Yes | Yes | NA | NA | Not specified | 6 |
| Zhang SY, 1998 | Yes | Yes | Yes | Yes | Not specified | Yes | Yes | NA | NA | Not specified | 6 |
| Zhao WC et al., 1998 | Yes | Yes | Yes | Yes | Not specified | Yes | Yes | NA | NA | Not specified | 6 |
| Li BS, 1998 | Yes | Yes | Yes | Yes | Not specified | Yes | Yes | NA | NA | Not specified | 6 |
| Lu JQ, 1998 | Yes | Yes | Yes | Yes | Not specified | Yes | Yes | NA | NA | Not specified | 6 |
| Yang ZG et al., 1998 | Yes | Yes | Yes | Yes | Not specified | Yes | Yes | NA | NA | Not specified | 6 |
| Xu ZX, 1994 | Yes | Yes | Yes | Yes | Not specified | Yes | Yes | NA | NA | Not specified | 6 |
| Feng SS, 1993 | Yes | Yes | Yes | Yes | Not specified | Yes | Yes | NA | NA | Not specified | 6 |
| Li DF, 1996 | Yes | Yes | Yes | Yes | Not specified | Yes | Yes | NA | NA | Not specified | 6 |
| Chen XL, 1992 | Yes | Yes | Yes | Yes | Not specified | Yes | Yes | NA | NA | Not specified | 6 |
| Liang XC, 1991 | Yes | Yes | Yes | Yes | Not specified | Yes | Yes | NA | NA | Not specified | 6 |
| Zhang MH et al., 1992 | Yes | Yes | Yes | Yes | Not specified | Yes | Yes | NA | NA | Not specified | 6 |
| Tan JM, 1990 | Yes | Yes | Yes | Yes | Not specified | Yes | Yes | NA | NA | Not specified | 6 |
| Zhuge CD, 1990 | Yes | Yes | Yes | Yes | Not specified | Yes | Yes | NA | NA | Not specified | 6 |
| Wen WH et al., 1989 | Yes | Yes | Yes | Yes | Not specified | Yes | Yes | NA | NA | Not specified | 6 |
| Yin AT, 1991 | Yes | Yes | Yes | Yes | Not specified | Yes | Yes | NA | NA | Not specified | 6 |
| Lin QM, 1990 | Yes | Yes | Yes | Yes | Not specified | Yes | Yes | NA | NA | Not specified | 6 |
| Ling LS, 1988 | Yes | Yes | Yes | Yes | Not specified | Yes | Yes | NA | NA | Not specified | 6 |
| Wen SQ, 1990 | Yes | Yes | Yes | Yes | Not specified | Yes | Yes | NA | NA | Not specified | 6 |
| Zhou CF et al., 1989 | Yes | Yes | Yes | Yes | Not specified | Yes | Yes | NA | NA | Not specified | 6 |
| Zhang TF, 1990 | Yes | Yes | Yes | Yes | Not specified | Yes | Yes | NA | NA | Not specified | 6 |
| Xing XG, 1988 | Yes | Yes | Yes | Yes | Not specified | Yes | Yes | NA | NA | Not specified | 6 |
| Zhou CJ et al., 1987 | Yes | Yes | Yes | Yes | Not specified | Yes | Yes | NA | NA | Not specified | 6 |
| Zhang KD, 1985 | Yes | Yes | Yes | Yes | Not specified | Yes | Yes | NA | NA | Not specified | 6 |
| Cao YP et al., 1985 | Yes | Yes | Yes | Yes | Not specified | Yes | Yes | NA | NA | Not specified | 6 |
| Dai YS et al., 1985 | Yes | Yes | Yes | Yes | Not specified | Yes | Yes | NA | NA | Not specified | 6 |
| Zheng FL et al., 1984 | Yes | Yes | Yes | Yes | Not specified | Yes | Yes | NA | NA | Not specified | 6 |
| Shen DR et al., 1984 | Yes | Yes | Yes | Yes | Not specified | Yes | Yes | NA | NA | Not specified | 6 |
| He CZ, 1987 | Yes | Yes | Yes | Yes | Not specified | Yes | Yes | NA | NA | Not specified | 6 |
| Feng YZ, 1983 | Yes | Yes | Yes | Yes | Not specified | Yes | Yes | NA | NA | Not specified | 6 |
| Journal of Parasitology and Parasitic Diseases,1983(02):15 | Yes | Yes | Yes | Yes | Not specified | Yes | Yes | NA | NA | Not specified | 6 |
| Xiao EX et al., 1992 | Yes | Yes | Yes | Yes | Not specified | Yes | Yes | NA | NA | Not specified | 6 |
| Li NY, 1984 | Yes | Yes | Yes | Yes | Not specified | Yes | Yes | NA | NA | Not specified | 6 |
| Yi PR et al., 1983 | Yes | Yes | Yes | Yes | Not specified | Yes | Yes | NA | NA | Not specified | 6 |
| Liu R et al., 1980 | Yes | Yes | Yes | Yes | Not specified | Yes | Yes | NA | NA | Not specified | 6 |
| Zhou SL et al., 1979 | Yes | Yes | Yes | Yes | Not specified | Yes | Yes | NA | NA | Not specified | 6 |
| Sun XL, 1984 | Yes | Yes | Yes | Yes | Not specified | Yes | Yes | NA | NA | Not specified | 6 |
| Zhu DY et al., 1998 | Yes | Yes | Yes | Yes | Not specified | Yes | Yes | NA | NA | Not specified | 6 |
| Dai NF et al., 1996 | Yes | Yes | Yes | Yes | Not specified | Yes | Yes | NA | NA | Not specified | 6 |
| Li HZ et al., 1993 | Yes | Yes | Yes | Yes | Not specified | Yes | Yes | NA | NA | Not specified | 6 |
| Zhang R, 1993 | Yes | Yes | Yes | Yes | Not specified | Yes | Yes | NA | NA | Not specified | 6 |
| Qiu MS et al., 1987 | Yes | Yes | Yes | Yes | Not specified | Yes | Yes | NA | NA | Not specified | 6 |
| Zhao CX et al., 2023 | Yes | Yes | Yes | Yes | Not specified | Yes | Yes | NA | NA | Not specified | 6 |
| Pang CM et al., 2022 | Yes | Yes | Yes | Yes | Not specified | Yes | Yes | NA | NA | Not specified | 6 |
| Tsai CC et al., 2024 | Yes | Yes | Yes | Yes | Not specified | Yes | Yes | NA | NA | Not specified | 6 |
| He YM et al., 1984 | Yes | Yes | Yes | Yes | Not specified | Yes | Yes | NA | NA | Not specified | 6 |
| Chen TY et al., 1975 | Yes | Yes | Yes | Yes | Not specified | Yes | Yes | NA | NA | Not specified | 6 |
| Tsou MH et al., 1993 | Yes | Yes | Yes | Yes | Not specified | Yes | Yes | NA | NA | Not specified | 6 |
| Oh MY et al., 2019 | Yes | Yes | Yes | Yes | Not specified | Yes | Yes | NA | NA | Not specified | 6 |
| Kwon OC et al., 2018 | Yes | Yes | Yes | Yes | Not specified | Yes | Yes | NA | NA | Not specified | 6 |
| Kim HS et al., 2017 | Yes | Yes | Yes | Yes | Not specified | Yes | Yes | NA | NA | Not specified | 6 |
| Min KW et al., 2013 | Yes | Yes | Yes | Yes | Not specified | Yes | Yes | NA | NA | Not specified | 6 |
| Ha S et al., 2013 | Yes | Yes | Yes | Yes | Not specified | Yes | Yes | NA | NA | Not specified | 6 |
| Koo M et al., 2011 | Yes | Yes | Yes | Yes | Not specified | Yes | Yes | NA | NA | Not specified | 6 |
| Moon HG et al., 2008 | Yes | Yes | Yes | Yes | Not specified | Yes | Yes | NA | NA | Not specified | 6 |
| Park JH et al., 2006 | Yes | Yes | Yes | Yes | Not specified | Yes | Yes | NA | NA | Not specified | 6 |
| Sim S et al., 2002 | Yes | Yes | Yes | Yes | Not specified | Yes | Yes | NA | NA | Not specified | 6 |
| Jeong et al., 2017 | Yes | Yes | Yes | Yes | Not specified | Yes | Yes | NA | NA | Not specified | 6 |
| 원태완 et al., 2015 | Yes | Yes | Yes | Yes | Not specified | Yes | Yes | NA | NA | Not specified | 6 |
| WOO OK HEE et al., 2015 | Yes | Yes | Yes | Yes | Not specified | Yes | Yes | NA | NA | Not specified | 6 |
| Ji-Young et al., 2015 | Yes | Yes | Yes | Yes | Not specified | Yes | Yes | NA | NA | Not specified | 6 |
| 김재운 et al., 2011 | Yes | Yes | Yes | Yes | Not specified | Yes | Yes | NA | NA | Not specified | 6 |
| Okino T et al., 2021 | Yes | Yes | Yes | Yes | Not specified | Yes | Yes | NA | NA | Not specified | 6 |
| Kazemi A et al., 2018 | Yes | Yes | Yes | Yes | Not specified | Yes | Yes | NA | NA | Not specified | 6 |
| Graham RP et al., 2014 | Yes | Yes | Yes | Yes | Not specified | Yes | Yes | NA | NA | Not specified | 6 |
| Samkeliso Blundell et al., 2022 | Yes | Yes | Yes | Yes | Not specified | Yes | Yes | NA | NA | Not specified | 6 |
| Chung SY et al., 1995 | Yes | Yes | Yes | Yes | Not specified | Yes | Yes | NA | NA | Not specified | 6 |
| Moreira MA et al., 1997 | Yes | Yes | Yes | Yes | Not specified | Yes | Yes | NA | NA | Not specified | 6 |
| Garin YJ et al., 1997 | Yes | Yes | Yes | Yes | Not specified | Yes | Yes | NA | NA | Not specified | 6 |
| Hwang M et al., 2020 | Yes | Yes | Yes | Yes | Not specified | Yes | Yes | NA | NA | Not specified | 6 |
| Kim BM et al., 2019 | Yes | Yes | Yes | Yes | Not specified | Yes | Yes | NA | NA | Not specified | 6 |
| Hwang JM et al., 2019 | Yes | Yes | Yes | Yes | Not specified | Yes | Yes | NA | NA | Not specified | 6 |
| Yang YJ et al., 2018 | Yes | Yes | Yes | Yes | Not specified | Yes | Yes | NA | NA | Not specified | 6 |
| Song EJ et al., 2015 | Yes | Yes | Yes | Yes | Not specified | Yes | Yes | NA | NA | Not specified | 6 |
| Lee EK et al., 2014 | Yes | Yes | Yes | Yes | Not specified | Yes | Yes | NA | NA | Not specified | 6 |
| Choi SJ et al., 2014 | Yes | Yes | Yes | Yes | Not specified | Yes | Yes | NA | NA | Not specified | 6 |
| Lee HM et al., 2014 | Yes | Yes | Yes | Yes | Not specified | Yes | Yes | NA | NA | Not specified | 6 |
| Lee YI et al., 2014 | Yes | Yes | Yes | Yes | Not specified | Yes | Yes | NA | NA | Not specified | 6 |
| Kim JI et al., 2014 | Yes | Yes | Yes | Yes | Not specified | Yes | Yes | NA | NA | Not specified | 6 |
| Roh SY et al., 2013 | Yes | Yes | Yes | Yes | Not specified | Yes | Yes | NA | NA | Not specified | 6 |
| Yoon HS et al., 2013 | Yes | Yes | Yes | Yes | Not specified | Yes | Yes | NA | NA | Not specified | 6 |
| Kim WY et al., 2013 | Yes | Yes | Yes | Yes | Not specified | Yes | Yes | NA | NA | Not specified | 6 |
| Yeo JY et al., 2012 | Yes | Yes | Yes | Yes | Not specified | Yes | Yes | NA | NA | Not specified | 6 |
| Wiwanitkit S et al., 2012 | Yes | Yes | Yes | Yes | Not specified | Yes | Yes | NA | NA | Not specified | 6 |
| Ha KY et al., 2011 | Yes | Yes | Yes | Yes | Not specified | Yes | Yes | NA | NA | Not specified | 6 |
| Lee KJ et al., 2010 | Yes | Yes | Yes | Yes | Not specified | Yes | Yes | NA | NA | Not specified | 6 |
| Kim HY et al., 2008 | Yes | Yes | Yes | Yes | Not specified | Yes | Yes | NA | NA | Not specified | 6 |
| Koo JH et al., 2006 | Yes | Yes | Yes | Yes | Not specified | Yes | Yes | NA | NA | Not specified | 6 |
| Lee JH et al., 2005 | Yes | Yes | Yes | Yes | Not specified | Yes | Yes | NA | NA | Not specified | 6 |
| Chae SW et al., 2003 | Yes | Yes | Yes | Yes | Not specified | Yes | Yes | NA | NA | Not specified | 6 |
| Kim SH et al., 2001 | Yes | Yes | Yes | Yes | Not specified | Yes | Yes | NA | NA | Not specified | 6 |
| Kim JR et al., 2001 | Yes | Yes | Yes | Yes | Not specified | Yes | Yes | NA | NA | Not specified | 6 |
| Kim SM et al., 2017 | Yes | Yes | Yes | Yes | Not specified | Yes | Yes | NA | NA | Not specified | 6 |
| Jeong Guk Jin et al., 2019 | Yes | Yes | Yes | Yes | Not specified | Yes | Yes | NA | NA | Not specified | 6 |
| YEON PARK JI et al., 2016 | Yes | Yes | Yes | Yes | Not specified | Yes | Yes | NA | NA | Not specified | 6 |
| Yun Sook Jung et al., 2013 | Yes | Yes | Yes | Yes | Not specified | Yes | Yes | NA | NA | Not specified | 6 |
| Sun-Ja Park et al., 2009 | Yes | Yes | Yes | Yes | Not specified | Yes | Yes | NA | NA | Not specified | 6 |
| 이학승 et al., 2007 | Yes | Yes | Yes | Yes | Not specified | Yes | Yes | NA | NA | Not specified | 6 |
| Kudo T et al., 2017 | Yes | Yes | Yes | Yes | Not specified | Yes | Yes | NA | NA | Not specified | 6 |
| Johnson G et al., 2015 | Yes | Yes | Yes | Yes | Not specified | Yes | Yes | NA | NA | Not specified | 6 |
| Tsuda H et al., 2014 | Yes | Yes | Yes | Yes | Not specified | Yes | Yes | NA | NA | Not specified | 6 |
| Nonomura Y et al., 2012 | Yes | Yes | Yes | Yes | Not specified | Yes | Yes | NA | NA | Not specified | 6 |
| Chiba T et al., 2012 | Yes | Yes | Yes | Yes | Not specified | Yes | Yes | NA | NA | Not specified | 6 |
| Sarukawa S et al., 2007 | Yes | Yes | Yes | Yes | Not specified | Yes | Yes | NA | NA | Not specified | 6 |
| Kimura S et al., 2003 | Yes | Yes | Yes | Yes | Not specified | Yes | Yes | NA | NA | Not specified | 6 |
| Fukatsu T et al., 2000 | Yes | Yes | Yes | Yes | Not specified | Yes | Yes | NA | NA | Not specified | 6 |
| Sabu L et al., 2015 | Yes | Yes | Yes | Yes | Not specified | Yes | Yes | NA | NA | Not specified | 6 |
| Ahn SK et al., 2019 | Yes | Yes | Yes | Yes | Not specified | Yes | Yes | NA | NA | Not specified | 6 |
| Tappe D et al., 2013 | Yes | Yes | Yes | Yes | Not specified | Yes | Yes | NA | NA | Not specified | 6 |
| Muigg V et al., 2019 | Yes | Yes | Yes | Yes | Not specified | Yes | Yes | NA | NA | Not specified | 6 |
| Omar HM et al., 2023 | Yes | Yes | Yes | Yes | Not specified | Yes | Yes | NA | NA | Not specified | 6 |
| Alvarez P et al., 2022 | Yes | Yes | Yes | Yes | Not specified | Yes | Yes | NA | NA | Not specified | 6 |
| Czyżewska J et al., 2019 | Yes | Yes | Yes | Yes | Not specified | Yes | Yes | NA | NA | Not specified | 6 |
| Schauer F et al., 2014 | Yes | Yes | Yes | Yes | Not specified | Yes | Yes | NA | NA | Not specified | 6 |
| Pampiglione S et al., 2003 | Yes | Yes | Yes | Yes | Not specified | Yes | Yes | NA | NA | Not specified | 6 |
| Bracaglia G et al., 2015 | Yes | Yes | Yes | Yes | Not specified | Yes | Yes | NA | NA | Not specified | 6 |
| Meric R et al., 2010 | Yes | Yes | Yes | Yes | Not specified | Yes | Yes | NA | NA | Not specified | 6 |
| Kim J et al., 1994 | Yes | Yes | Yes | Yes | Not specified | Yes | Yes | NA | NA | Not specified | 6 |
| Nakamura T et al., 1990 | Yes | Yes | Yes | Yes | Not specified | Yes | Yes | NA | NA | Not specified | 6 |
| Yamane Y et al., 1975 | Yes | Yes | Yes | Yes | Not specified | Yes | Yes | NA | NA | Not specified | 6 |
| Griffin MP et al., 1996 | Yes | Yes | Yes | Yes | Not specified | Yes | Yes | NA | NA | Not specified | 6 |
| Corrall CJ et al., 1987 | Yes | Yes | Yes | Yes | Not specified | Yes | Yes | NA | NA | Not specified | 6 |
| Sarma DP et al., 1986 | Yes | Yes | Yes | Yes | Not specified | Yes | Yes | NA | NA | Not specified | 6 |
| Taylor RL et al., 1976 | Yes | Yes | Yes | Yes | Not specified | Yes | Yes | NA | NA | Not specified | 6 |
| MARKELL EK et al., 1964 | Yes | Yes | Yes | Yes | Not specified | Yes | Yes | NA | NA | Not specified | 6 |
| STRAUSS WG et al., 1964 | Yes | Yes | Yes | Yes | Not specified | Yes | Yes | NA | NA | Not specified | 6 |
| SHORT RB et al., 1964 | Yes | Yes | Yes | Yes | Not specified | Yes | Yes | NA | NA | Not specified | 6 |
| WIRTH WA et al., 1961 | Yes | Yes | Yes | Yes | Not specified | Yes | Yes | NA | NA | Not specified | 6 |
| BROOKS TJ Jr et al., 1960 | Yes | Yes | Yes | Yes | Not specified | Yes | Yes | NA | NA | Not specified | 6 |
| WEINSTEIN PP et al., 1954 | Yes | Yes | Yes | Yes | Not specified | Yes | Yes | NA | NA | Not specified | 6 |
| Vortel V et al., 1995 | Yes | Yes | Yes | Yes | Not specified | Yes | Yes | NA | NA | Not specified | 6 |
| Campbell EW et al., 1977 | Yes | Yes | Yes | Yes | Not specified | Yes | Yes | NA | NA | Not specified | 6 |
| Ali-Khan Z et al., 1973 | Yes | Yes | Yes | Yes | Not specified | Yes | Yes | NA | NA | Not specified | 6 |
| FAIN A et al., 1959 | Yes | Yes | Yes | Yes | Not specified | Yes | Yes | NA | NA | Not specified | 6 |
| FOSTER R et al., 1965 | Yes | Yes | Yes | No | Not specified | Yes | Yes | NA | NA | Not specified | 5 |
| ALVES WD et al., 1954 | Yes | Yes | Yes | Yes | Not specified | Yes | Yes | NA | NA | Not specified | 6 |
| de Roodt AR et al., 1993 | Yes | Yes | Yes | Yes | Not specified | Yes | Yes | NA | NA | Not specified | 6 |
| Kron MA et al., 1991 | Yes | Yes | Yes | Yes | Not specified | Yes | Yes | NA | NA | Not specified | 6 |
| Guderian R et al., 1990 | Yes | Yes | Yes | Yes | Not specified | Yes | Yes | NA | NA | Not specified | 6 |
| Wijesundera MS et al., 1997 | Yes | Yes | Yes | No | Not specified | Yes | Yes | NA | NA | Not specified | 5 |
| YOGORE MG Jr et al., 1953 | Yes | Yes | Yes | No | Not specified | Yes | Yes | NA | NA | Not specified | 5 |
| GARCIA EY et al., 1950 | Yes | Yes | Yes | No | Not specified | Yes | Yes | NA | NA | Not specified | 5 |
| Berdonosova TI et al., 1968 | Yes | Yes | Yes | No | Not specified | Yes | Yes | NA | NA | Not specified | 5 |
| READ CP et al., 1952 | Yes | Yes | Yes | Yes | Not specified | Yes | Yes | NA | NA | Not specified | 6 |
| Cooke RA et al., 1983 | Yes | Yes | Yes | Yes | Not specified | Yes | Yes | NA | NA | Not specified | 6 |
| SANDARS DF et al., 1954 | Yes | Yes | Yes | Yes | Not specified | Yes | Yes | NA | NA | Not specified | 6 |
| Xu YW et al., 2021 | Yes | Yes | Yes | Yes | Not specified | Yes | Yes | NA | NA | Not specified | 6 |
| Niu Z et al., 2024 | Yes | Yes | Yes | Yes | Not specified | Yes | Yes | NA | NA | Not specified | 6 |
| Wu D et al., 2024 | Yes | Yes | Yes | Yes | Not specified | Yes | Yes | NA | NA | Not specified | 6 |
| Mo G et al., 2020 | Yes | Yes | Yes | Yes | Not specified | Yes | Yes | NA | NA | Not specified | 6 |
| Pan SH et al., 2017 | Yes | Yes | Yes | Yes | Not specified | Yes | Yes | NA | NA | Not specified | 6 |
| Fan XY, 2016 | Yes | Yes | Yes | Yes | Not specified | Yes | Yes | NA | NA | Not specified | 6 |
| Shen JH, 2012 | Yes | Yes | Yes | Yes | Not specified | Yes | Yes | NA | NA | Not specified | 6 |
| Chen XH, 2012 | Yes | Yes | Yes | Yes | Not specified | Yes | Yes | NA | NA | Not specified | 6 |
| Cheng K B et al., 2014 | Yes | Yes | Yes | Yes | Not specified | Yes | Yes | NA | NA | Not specified | 6 |
| Huang F et al., 2012 | Yes | Yes | Yes | Yes | Not specified | Yes | Yes | NA | NA | Not specified | 6 |
| Cheng KB et al., 2011 | Yes | Yes | Yes | Yes | Not specified | Yes | Yes | NA | NA | Not specified | 6 |
| Chen HN et al., 2002 | Yes | Yes | Yes | Yes | Not specified | Yes | Yes | NA | NA | Not specified | 6 |
| Wang RL et al., 1999 | Yes | Yes | Yes | Yes | Not specified | Yes | Yes | NA | NA | Not specified | 6 |
| Zhao DJ et al., 2014 | Yes | Yes | Yes | Yes | Not specified | Yes | Yes | NA | NA | Not specified | 6 |
| Li N et al., 2015 | Yes | Yes | Yes | No | Not specified | Yes | Yes | NA | NA | Not specified | 5 |
| Zhao ZH et al., 2023 | Yes | Yes | Yes | Yes | Not specified | Yes | Yes | NA | NA | Not specified | 6 |
| Zong LB et al., 2023 | Yes | Yes | Yes | Yes | Not specified | Yes | Yes | NA | NA | Not specified | 6 |
| Deng B et al., 2023 | Yes | Yes | Yes | Yes | Not specified | Yes | Yes | NA | NA | Not specified | 6 |
| Chen Q et al., 2020 | Yes | Yes | Yes | Yes | Not specified | Yes | Yes | NA | NA | Not specified | 6 |
| Deng K et al., 2019 | Yes | Yes | Yes | Yes | Not specified | Yes | Yes | NA | NA | Not specified | 6 |
| Huang M et al., 2019 | Yes | Yes | Yes | Yes | Not specified | Yes | Yes | NA | NA | Not specified | 6 |
| Zhu YN et al., 2018 | Yes | Yes | Yes | Yes | Not specified | Yes | Yes | NA | NA | Not specified | 6 |
| Tao LS, 2018, | Yes | Yes | Yes | Yes | Not specified | Yes | Yes | NA | NA | Not specified | 6 |
| Qian QM, 2018 | Yes | Yes | Yes | Yes | Not specified | Yes | Yes | NA | NA | Not specified | 6 |
| Cui J et al., 2017 | Yes | Yes | Yes | Yes | Not specified | Yes | Yes | NA | NA | Not specified | 6 |
| Zhu H et al., 2019 | Yes | Yes | Yes | Yes | Not specified | Yes | Yes | NA | NA | Not specified | 6 |
| Lin Quan et al., 2015 | Yes | Yes | Yes | Yes | Not specified | Yes | Yes | NA | NA | Not specified | 6 |
| Tan ZR et al., 2015 | Yes | Yes | Yes | Yes | Not specified | Yes | Yes | NA | NA | Not specified | 6 |
| Ouyang JS et al., 2014 | Yes | Yes | Yes | Yes | Not specified | Yes | Yes | NA | NA | Not specified | 6 |
| Yang Y et al., 2015 | Yes | Yes | Yes | Yes | Not specified | Yes | Yes | NA | NA | Not specified | 6 |
| Wang H et al., 2011 | Yes | Yes | Yes | No | Not specified | Yes | Yes | NA | NA | Not specified | 5 |
| Zheng NC et al., 2007 | Yes | Yes | Yes | Yes | Not specified | Yes | Yes | NA | NA | Not specified | 6 |
| Xie XB et al., 2010 | Yes | Yes | Yes | Yes | Not specified | Yes | Yes | NA | NA | Not specified | 6 |
| Cui LJ et al., 1992 | Yes | Yes | Yes | Yes | Not specified | Yes | Yes | NA | NA | Not specified | 6 |
| Wang Y et al., 2024 | Yes | Yes | Yes | Yes | Not specified | Yes | Yes | NA | NA | Not specified | 6 |
| Jo GD et al., 2016 | Yes | Yes | Yes | Yes | Not specified | Yes | Yes | NA | NA | Not specified | 6 |
| Kim JK et al., 2016 | Yes | Yes | Yes | Yes | Not specified | Yes | Yes | NA | NA | Not specified | 6 |
| Oh Y et al., 2014 | Yes | Yes | Yes | Yes | Not specified | Yes | Yes | NA | NA | Not specified | 6 |
| Chung SW et al., 2012 | Yes | Yes | Yes | Yes | Not specified | Yes | Yes | NA | NA | Not specified | 6 |
| Lee JH et al., 2011 | Yes | Yes | Yes | Yes | Not specified | Yes | Yes | NA | NA | Not specified | 6 |
| Jang, Ji-Woong et al., 2012 | Yes | Yes | Yes | Yes | Not specified | Yes | Yes | NA | NA | Not specified | 6 |
| 오윤정 et al., 2009 | Yes | Yes | Yes | Yes | Not specified | Yes | Yes | NA | NA | Not specified | 6 |
| Kim Hyo Jong et al., 2009 | Yes | Yes | Yes | Yes | Not specified | Yes | Yes | NA | NA | Not specified | 6 |
| Youn Hyo-Chul et al., 2006 | Yes | Yes | Yes | Yes | Not specified | Yes | Yes | NA | NA | Not specified | 6 |
| 허진 et al., 2003 | Yes | Yes | Yes | Yes | Not specified | Yes | Yes | NA | NA | Not specified | 6 |
| Matsuki M et al., 2021 | Yes | Yes | Yes | Yes | Not specified | Yes | Yes | NA | NA | Not specified | 6 |
| Takeda K et al., 2016 | Yes | Yes | Yes | Yes | Not specified | Yes | Yes | NA | NA | Not specified | 6 |
| Iwatani K et al., 2006 | Yes | Yes | Yes | Yes | Not specified | Yes | Yes | NA | NA | Not specified | 6 |
| Kamiya H et al., 2004 | Yes | Yes | Yes | Yes | Not specified | Yes | Yes | NA | NA | Not specified | 6 |
| Ishii H et al., 2001 | Yes | Yes | Yes | Yes | Not specified | Yes | Yes | NA | NA | Not specified | 6 |
| Phunmanee A et al., 2001 | Yes | Yes | Yes | Yes | Not specified | Yes | Yes | NA | NA | Not specified | 6 |
| Khurana S et al., 2012 | Yes | Yes | Yes | Yes | Not specified | Yes | Yes | NA | NA | Not specified | 6 |
| Duggal S et al., 2011 | Yes | Yes | Yes | Yes | Not specified | Yes | Yes | NA | NA | Not specified | 6 |
| Cho KJ et al., 1987 | Yes | Yes | Yes | Yes | Not specified | Yes | Yes | NA | NA | Not specified | 6 |
| Min HK et al., 1976 | Yes | Yes | Yes | Yes | Not specified | Yes | Yes | NA | NA | Not specified | 6 |
| Li YS et al., 2023 | Yes | Yes | Yes | Yes | Not specified | Yes | Yes | NA | NA | Not specified | 6 |
| Rong ZL et al., 2022 | Yes | Yes | Yes | Yes | Not specified | Yes | Yes | NA | NA | Not specified | 6 |
| Jialing Hu et al., 2022 | Yes | Yes | Yes | Yes | Not specified | Yes | Yes | NA | NA | Not specified | 6 |
| Liang CY et al., 2021 | Yes | Yes | Yes | Yes | Not specified | Yes | Yes | NA | NA | Not specified | 6 |
| Yanming Meng et al., 2019 | Yes | Yes | Yes | Yes | Not specified | Yes | Yes | NA | NA | Not specified | 6 |
| You L et al., 2022 | Yes | Yes | Yes | Yes | Not specified | Yes | Yes | NA | NA | Not specified | 6 |
| Liu C et al., 2021 | Yes | Yes | Yes | Yes | Not specified | Yes | Yes | NA | NA | Not specified | 6 |
| Zhu YL et al., 2019 | Yes | Yes | Yes | Yes | Not specified | Yes | Yes | NA | NA | Not specified | 6 |
| Pan LY et al., 2021 | Yes | Yes | Yes | Yes | Not specified | Yes | Yes | NA | NA | Not specified | 6 |
| Tang YM et al., 2017 | Yes | Yes | Yes | Yes | Not specified | Yes | Yes | NA | NA | Not specified | 6 |
| Wu L et al., 2017 | Yes | Yes | Yes | Yes | Not specified | Yes | Yes | NA | NA | Not specified | 6 |
| Xie D et al., 2019 | Yes | Yes | Yes | Yes | Not specified | Yes | Yes | NA | NA | Not specified | 6 |
| Wei TH et al., 2017 | Yes | Yes | Yes | Yes | Not specified | Yes | Yes | NA | NA | Not specified | 6 |
| Chen XW et al., 2023 | Yes | Yes | Yes | Yes | Not specified | Yes | Yes | NA | NA | Not specified | 6 |
| Huang WB et al., 2020 | Yes | Yes | Yes | Yes | Not specified | Yes | Yes | NA | NA | Not specified | 6 |
| Ma Y et al., 2018 | Yes | Yes | Yes | Yes | Not specified | Yes | Yes | NA | NA | Not specified | 6 |
| Wang Y et al., 2017 | Yes | Yes | Yes | Yes | Not specified | Yes | Yes | NA | NA | Not specified | 6 |
| Du J et al., 2015 | Yes | Yes | Yes | Yes | Not specified | Yes | Yes | NA | NA | Not specified | 6 |
| Cai YC et al., 2015 | Yes | Yes | Yes | Yes | Not specified | Yes | Yes | NA | NA | Not specified | 6 |
| Bennett H M et al., 2014 | Yes | Yes | Yes | Yes | Not specified | Yes | Yes | NA | NA | Not specified | 6 |
| Xiong YB et al., 2018 | Yes | Yes | Yes | Yes | Not specified | Yes | Yes | NA | NA | Not specified | 6 |
| Liang Y et al., 2015 | Yes | Yes | Yes | Yes | Not specified | Yes | Yes | NA | NA | Not specified | 6 |
| Dong QW et al., 2014 | Yes | Yes | Yes | Yes | Not specified | Yes | Yes | NA | NA | Not specified | 6 |
| Huang DW et al., 2014 | Yes | Yes | Yes | Yes | Not specified | Yes | Yes | NA | NA | Not specified | 6 |
| Pan F et al., 2013 | Yes | Yes | Yes | Yes | Not specified | Yes | Yes | NA | NA | Not specified | 6 |
| Li DT et al., 2012 | Yes | Yes | Yes | Yes | Not specified | Yes | Yes | NA | NA | Not specified | 6 |
| Gao B et al., 2012 | Yes | Yes | Yes | Yes | Not specified | Yes | Yes | NA | NA | Not specified | 6 |
| Zeng T et al., 2012 | Yes | Yes | Yes | Yes | Not specified | Yes | Yes | NA | NA | Not specified | 6 |
| Zhu XZ et al., 2012 | Yes | Yes | Yes | No | Not specified | Yes | Yes | NA | NA | Not specified | 5 |
| Chen HF et al., 2012 | Yes | Yes | Yes | Yes | Not specified | Yes | Yes | NA | NA | Not specified | 6 |
| Wang P et al., 2012 | Yes | Yes | Yes | Yes | Not specified | Yes | Yes | NA | NA | Not specified | 6 |
| Xu SQ et al., 2011 | Yes | Yes | Yes | Yes | Not specified | Yes | Yes | NA | NA | Not specified | 6 |
| Xu WJ et al., 2012 | Yes | Yes | Yes | Yes | Not specified | Yes | Yes | NA | NA | Not specified | 6 |
| Chen H et al., 2011 | Yes | Yes | Yes | Yes | Not specified | Yes | Yes | NA | NA | Not specified | 6 |
| Yi XM et al., 2010 | Yes | Yes | Yes | Yes | Not specified | Yes | Yes | NA | NA | Not specified | 6 |
| Wang XP et al., 2010 | Yes | Yes | Yes | Yes | Not specified | Yes | Yes | NA | NA | Not specified | 6 |
| Ou Q et al., 2010 | Yes | Yes | Yes | Yes | Not specified | Yes | Yes | NA | NA | Not specified | 6 |
| Chiu C H et al., 2010 | Yes | Yes | Yes | Yes | Not specified | Yes | Yes | NA | NA | Not specified | 6 |
| Lai PF et al., 2010 | Yes | Yes | Yes | Yes | Not specified | Yes | Yes | NA | NA | Not specified | 6 |
| Huang CC et al., 2010 | Yes | Yes | Yes | Yes | Not specified | Yes | Yes | NA | NA | Not specified | 6 |
| Wang Y et al., 2009 | Yes | Yes | Yes | Yes | Not specified | Yes | Yes | NA | NA | Not specified | 6 |
| Jiang BD et al., 2009 | Yes | Yes | Yes | Yes | Not specified | Yes | Yes | NA | NA | Not specified | 6 |
| Lei ZY et al., 2010 | Yes | Yes | Yes | Yes | Not specified | Yes | Yes | NA | NA | Not specified | 6 |
| Li SJ et al., 2010 | Yes | Yes | Yes | Yes | Not specified | Yes | Yes | NA | NA | Not specified | 6 |
| Jin ZG et al., 2008 | Yes | Yes | Yes | Yes | Not specified | Yes | Yes | NA | NA | Not specified | 6 |
| Ya Y, 2008 | Yes | Yes | Yes | Yes | Not specified | Yes | Yes | NA | NA | Not specified | 6 |
| Deng L et al., 2009 | Yes | Yes | Yes | Yes | Not specified | Yes | Yes | NA | NA | Not specified | 6 |
| Li HF et al., 2008 | Yes | Yes | Yes | Yes | Not specified | Yes | Yes | NA | NA | Not specified | 6 |
| Bo YL et al., 2007 | Yes | Yes | Yes | Yes | Not specified | Yes | Yes | NA | NA | Not specified | 6 |
| Cheng ZC et al., 2007 | Yes | Yes | Yes | Yes | Not specified | Yes | Yes | NA | NA | Not specified | 6 |
| Lv T et al., 2007 | Yes | Yes | Yes | Yes | Not specified | Yes | Yes | NA | NA | Not specified | 6 |
| Zhou JG et al., 2008 | Yes | Yes | Yes | Yes | Not specified | Yes | Yes | NA | NA | Not specified | 6 |
| Liang XQ et al., 2008 | Yes | Yes | Yes | Yes | Not specified | Yes | Yes | NA | NA | Not specified | 6 |
| Gong YX, 2007 | Yes | Yes | Yes | Yes | Not specified | Yes | Yes | NA | NA | Not specified | 6 |
| Gao B et al., 2006. | Yes | Yes | Yes | Yes | Not specified | Yes | Yes | NA | NA | Not specified | 6 |
| Gao LM et al., 2005 | Yes | Yes | Yes | Yes | Not specified | Yes | Yes | NA | NA | Not specified | 6 |
| Zheng Z et al., 2007 | Yes | Yes | Yes | Yes | Not specified | Yes | Yes | NA | NA | Not specified | 6 |
| Zheng YG et al., 2005 | Yes | Yes | Yes | Yes | Not specified | Yes | Yes | NA | NA | Not specified | 6 |
| Gao XL et al., 2006 | Yes | Yes | Yes | Yes | Not specified | Yes | Yes | NA | NA | Not specified | 6 |
| Lu YH et al., 2003 | Yes | Yes | Yes | Yes | Not specified | Yes | Yes | NA | NA | Not specified | 6 |
| Zheng Z, 2003 | Yes | Yes | Yes | Yes | Not specified | Yes | Yes | NA | NA | Not specified | 6 |
| Ke ZB et al., 2004 | Yes | Yes | Yes | Yes | Not specified | Yes | Yes | NA | NA | Not specified | 6 |
| Huang CH et al., 2003 | Yes | Yes | Yes | Yes | Not specified | Yes | Yes | NA | NA | Not specified | 6 |
| Fang ZM et al., 2003 | Yes | Yes | Yes | Yes | Not specified | Yes | Yes | NA | NA | Not specified | 6 |
| Zhao XL et al., 2002 | Yes | Yes | Yes | Yes | Not specified | Yes | Yes | NA | NA | Not specified | 6 |
| Jin XX et al., 2002 | Yes | Yes | Yes | Yes | Not specified | Yes | Yes | NA | NA | Not specified | 6 |
| Nie CX, 2002 | Yes | Yes | Yes | Yes | Not specified | Yes | Yes | NA | NA | Not specified | 6 |
| Li XJ et al., 2001 | Yes | Yes | Yes | Yes | Not specified | Yes | Yes | NA | NA | Not specified | 6 |
| Zhao YG et al., 1999 | Yes | Yes | Yes | Yes | Not specified | Yes | Yes | NA | NA | Not specified | 6 |
| Zhou YQ et al., 1996 | Yes | Yes | Yes | Yes | Not specified | Yes | Yes | NA | NA | Not specified | 6 |
| You D L et al., 1997 | Yes | Yes | Yes | Yes | Not specified | Yes | Yes | NA | NA | Not specified | 6 |
| Liu JC, 1996 | Yes | Yes | Yes | Yes | Not specified | Yes | Yes | NA | NA | Not specified | 6 |
| Chen MZ et al., 1995 | Yes | Yes | Yes | Yes | Not specified | Yes | Yes | NA | NA | Not specified | 6 |
| Liang C et al., 1999 | Yes | Yes | Yes | Yes | Not specified | Yes | Yes | NA | NA | Not specified | 6 |
| Wong CW et al., 1994 | Yes | Yes | Yes | Yes | Not specified | Yes | Yes | NA | NA | Not specified | 6 |
| Liang SM et al., 1994 | Yes | Yes | Yes | Yes | Not specified | Yes | Yes | NA | NA | Not specified | 6 |
| Deng YF et al., 1995 | Yes | Yes | Yes | Yes | Not specified | Yes | Yes | NA | NA | Not specified | 6 |
| Hu WA, 1994 | Yes | Yes | Yes | Yes | Not specified | Yes | Yes | NA | NA | Not specified | 6 |
| Pan L et al., 1994 | Yes | Yes | Yes | Yes | Not specified | Yes | Yes | NA | NA | Not specified | 6 |
| Wang SC, 1994 | Yes | Yes | Yes | Yes | Not specified | Yes | Yes | NA | NA | Not specified | 6 |
| Xu T et al., 1992 | Yes | Yes | Yes | Yes | Not specified | Yes | Yes | NA | NA | Not specified | 6 |
| Xuan HS et al., 1991 | Yes | Yes | Yes | Yes | Not specified | Yes | Yes | NA | NA | Not specified | 6 |
| Liu ZH et al., 1990 | Yes | Yes | Yes | Yes | Not specified | Yes | Yes | NA | NA | Not specified | 6 |
| Du B, 1988 | Yes | Yes | Yes | Yes | Not specified | Yes | Yes | NA | NA | Not specified | 6 |
| Chan S T et al., 1987 | Yes | Yes | Yes | Yes | Not specified | Yes | Yes | NA | NA | Not specified | 6 |
| Niu Q, 1995 | Yes | Yes | Yes | Yes | Not specified | Yes | Yes | NA | NA | Not specified | 6 |
| Ru JS et al., 1985 | Yes | Yes | Yes | Yes | Not specified | Yes | Yes | NA | NA | Not specified | 6 |
| Jiang JN et al., 1987 | Yes | Yes | Yes | Yes | Not specified | Yes | Yes | NA | NA | Not specified | 6 |
| Zhang FL et al., 1983 | Yes | Yes | Yes | Yes | Not specified | Yes | Yes | NA | NA | Not specified | 6 |
| Wang GB et al., 2024 | Yes | Yes | Yes | Yes | Not specified | Yes | Yes | NA | NA | Not specified | 6 |
| Xie HQ et al., 2022 | Yes | Yes | Yes | Yes | Not specified | Yes | Yes | NA | NA | Not specified | 6 |
| Li F et al., 2022 | Yes | Yes | Yes | Yes | Not specified | Yes | Yes | NA | NA | Not specified | 6 |
| Yan XQ et al., 2021 | Yes | Yes | Yes | Yes | Not specified | Yes | Yes | NA | NA | Not specified | 6 |
| He XH et al., 2021 | Yes | Yes | Yes | Yes | Not specified | Yes | Yes | NA | NA | Not specified | 6 |
| Chen LF et al., 2019 | Yes | Yes | Yes | Yes | Not specified | Yes | Yes | NA | NA | Not specified | 6 |
| Zhang P et al., 2019 | Yes | Yes | Yes | Yes | Not specified | Yes | Yes | NA | NA | Not specified | 6 |
| Fu PC et al., 2019 | Yes | Yes | Yes | Yes | Not specified | Yes | Yes | NA | NA | Not specified | 6 |
| Liu CH et al., 2019 | Yes | Yes | Yes | Yes | Not specified | Yes | Yes | NA | NA | Not specified | 6 |
| Ye SK et al., 2018 | Yes | Yes | Yes | Yes | Not specified | Yes | Yes | NA | NA | Not specified | 6 |
| Cao G, 2018 | Yes | Yes | Yes | No | Not specified | Yes | Yes | NA | NA | Not specified | 5 |
| Hong DJ et al., 2018 | Yes | Yes | Yes | No | Not specified | Yes | Yes | NA | NA | Not specified | 5 |
| Ding YJ et al., 2017 | Yes | Yes | Yes | Yes | Not specified | Yes | Yes | NA | NA | Not specified | 6 |
| Yang C et al., 2017 | Yes | Yes | Yes | Yes | Not specified | Yes | Yes | NA | NA | Not specified | 6 |
| Wang L et al., 2016 | Yes | Yes | Yes | Yes | Not specified | Yes | Yes | NA | NA | Not specified | 6 |
| Liao HY et al., 2016 | Yes | Yes | Yes | Yes | Not specified | Yes | Yes | NA | NA | Not specified | 6 |
| Yu Y et al., 2016 | Yes | Yes | Yes | Yes | Not specified | Yes | Yes | NA | NA | Not specified | 6 |
| Xie HQ et al., 2015 | Yes | Yes | Yes | Yes | Not specified | Yes | Yes | NA | NA | Not specified | 6 |
| Chen F et al., 2014 | Yes | Yes | Yes | No | Not specified | Yes | Yes | NA | NA | Not specified | 5 |
| Liu RW, 2014 | Yes | Yes | Yes | Yes | Not specified | Yes | Yes | NA | NA | Not specified | 6 |
| Yang LX et al., 2014 | Yes | Yes | Yes | No | Not specified | Yes | Yes | NA | NA | Not specified | 5 |
| Luo ZY, 2013 | Yes | Yes | Yes | Yes | Not specified | Yes | Yes | NA | NA | Not specified | 6 |
| Chu S et al., 2013 | Yes | Yes | Yes | Yes | Not specified | Yes | Yes | NA | NA | Not specified | 6 |
| Li YX et al., 2013 | Yes | Yes | Yes | Yes | Not specified | Yes | Yes | NA | NA | Not specified | 6 |
| Tu YR, 2012 | Yes | Yes | Yes | No | Not specified | Yes | Yes | NA | NA | Not specified | 5 |
| Gong C et al., 2012 | Yes | Yes | Yes | Yes | Not specified | Yes | Yes | NA | NA | Not specified | 6 |
| Hou XY et al., 2012 | Yes | Yes | Yes | Yes | Not specified | Yes | Yes | NA | NA | Not specified | 6 |
| Kang Z et al., 2011 | Yes | Yes | Yes | No | Not specified | Yes | Yes | NA | NA | Not specified | 5 |
| Ding YJ et al., 2011 | Yes | Yes | Yes | Yes | Not specified | Yes | Yes | NA | NA | Not specified | 6 |
| Jiang MC et al., 2011 | Yes | Yes | Yes | Yes | Not specified | Yes | Yes | NA | NA | Not specified | 6 |
| Chen XH et al., 2010 | Yes | Yes | Yes | Yes | Not specified | Yes | Yes | NA | NA | Not specified | 6 |
| Wang SM et al., 2009 | Yes | Yes | Yes | No | Not specified | Yes | Yes | NA | NA | Not specified | 5 |
| Zhang L, 2009 | Yes | Yes | Yes | Yes | Not specified | Yes | Yes | NA | NA | Not specified | 6 |
| Gong CG et al., 2008 | Yes | Yes | Yes | Yes | Not specified | Yes | Yes | NA | NA | Not specified | 6 |
| Guo EP et al., 2004 | Yes | Yes | Yes | Yes | Not specified | Yes | Yes | NA | NA | Not specified | 6 |
| Xia ZM et al., 2008 | Yes | Yes | Yes | Yes | Not specified | Yes | Yes | NA | NA | Not specified | 6 |
| Song T et al., 2007 | Yes | Yes | Yes | Yes | Not specified | Yes | Yes | NA | NA | Not specified | 6 |
| Gong CG et al., 2006 | Yes | Yes | Yes | Yes | Not specified | Yes | Yes | NA | NA | Not specified | 6 |
| Qin F et al., 2003 | Yes | Yes | Yes | No | Not specified | Yes | Yes | NA | NA | Not specified | 5 |
| Chen h et al., 2003 | Yes | Yes | Yes | Yes | Not specified | Yes | Yes | NA | NA | Not specified | 6 |
| Huang XH, 2002 | Yes | Yes | Yes | Yes | Not specified | Yes | Yes | NA | NA | Not specified | 6 |
| Yi CY et al., 2002 | Yes | Yes | Yes | No | Not specified | Yes | Yes | NA | NA | Not specified | 5 |
| Chen h et al., 1999 | Yes | Yes | Yes | Yes | Not specified | Yes | Yes | NA | NA | Not specified | 6 |
| Gu YJ, 1997 | Yes | Yes | Yes | Yes | Not specified | Yes | Yes | NA | NA | Not specified | 6 |
| Chu SG et al., 2006 | Yes | Yes | Yes | No | Not specified | Yes | Yes | NA | NA | Not specified | 5 |
| Wang HD et al., 1996 | Yes | Yes | Yes | Yes | Not specified | Yes | Yes | NA | NA | Not specified | 6 |
| Yang QC, 1994 | Yes | Yes | Yes | No | Not specified | Yes | Yes | NA | NA | Not specified | 5 |
| Chen YS et al., 2022 | Yes | Yes | Yes | Yes | Not specified | Yes | Yes | NA | NA | Not specified | 6 |
| Zhu YZ et al., 2012 | Yes | Yes | Yes | Yes | Not specified | Yes | Yes | NA | NA | Not specified | 6 |
| Chen XW et al., 2022 | Yes | Yes | Yes | Yes | Not specified | Yes | Yes | NA | NA | Not specified | 6 |
| Hameed NUF et al., 2018 | Yes | Yes | Yes | Yes | Not specified | Yes | Yes | NA | NA | Not specified | 6 |
| Tsai MD et al., 1993 | Yes | Yes | Yes | Yes | Not specified | Yes | Yes | NA | NA | Not specified | 6 |
| Bo G et al., 2006 | Yes | Yes | Yes | Yes | Not specified | Yes | Yes | NA | NA | Not specified | 6 |
| Zhao QQ et al., 2023 | Yes | Yes | Yes | Yes | Not specified | Yes | Yes | NA | NA | Not specified | 6 |
| Bao XY et al., 2008 | Yes | Yes | Yes | Yes | Not specified | Yes | Yes | NA | NA | Not specified | 6 |
| Guo J et al., 2007 | Yes | Yes | Yes | Yes | Not specified | Yes | Yes | NA | NA | Not specified | 6 |
| Zhao HM et al., 1998 | Yes | Yes | Yes | Yes | Not specified | Yes | Yes | NA | NA | Not specified | 6 |
| Li XR, 2001 | Yes | Yes | Yes | No | Not specified | Yes | Yes | NA | NA | Not specified | 5 |
| Liao LQ et al., 1991 | Yes | Yes | Yes | Yes | Not specified | Yes | Yes | NA | NA | Not specified | 6 |
| Wu GY et al., 1990 | Yes | Yes | Yes | Yes | Not specified | Yes | Yes | NA | NA | Not specified | 6 |
| Cao Y et al., 2021 | Yes | Yes | Yes | Yes | Not specified | Yes | Yes | NA | NA | Not specified | 6 |
| Hou Z et al., 2017 | Yes | Yes | Yes | Yes | Not specified | Yes | Yes | NA | NA | Not specified | 6 |
| Liu YJ, 2006 | Yes | Yes | Yes | Yes | Not specified | Yes | Yes | NA | NA | Not specified | 6 |
| Zhang XZ et al., 1987 | Yes | Yes | Yes | Yes | Not specified | Yes | Yes | NA | NA | Not specified | 6 |
| Yang JH et al., 2021 | Yes | Yes | Yes | Yes | Not specified | Yes | Yes | NA | NA | Not specified | 6 |
| Liu J et al., 2022 | Yes | Yes | Yes | Yes | Not specified | Yes | Yes | NA | NA | Not specified | 6 |
| Meng YM et al., 2023 | Yes | Yes | Yes | Yes | Not specified | Yes | Yes | NA | NA | Not specified | 6 |
| Fan JF et al., 2021 | Yes | Yes | Yes | Yes | Not specified | Yes | Yes | NA | NA | Not specified | 6 |
| Xing WR et al., 2021 | Yes | Yes | Yes | Yes | Not specified | Yes | Yes | NA | NA | Not specified | 6 |
| Fung CF et al., 1989 | Yes | Yes | Yes | Yes | Not specified | Yes | Yes | NA | NA | Not specified | 6 |
| Lo YK et al., 1987 | Yes | Yes | Yes | Yes | Not specified | Yes | Yes | NA | NA | Not specified | 6 |
| Tang TW et al., 2011 | Yes | Yes | Yes | Yes | Not specified | Yes | Yes | NA | NA | Not specified | 6 |
| Huang CT et al., 2012 | Yes | Yes | Yes | Yes | Not specified | Yes | Yes | NA | NA | Not specified | 6 |
| Liao SW et al., 1984 | Yes | Yes | Yes | No | Not specified | Yes | Yes | NA | NA | Not specified | 5 |
| Wen GJ et al., 2023 | Yes | Yes | Yes | Yes | Not specified | Yes | Yes | NA | NA | Not specified | 6 |
| Hwang YH et al., 2020 | Yes | Yes | Yes | Yes | Not specified | Yes | Yes | NA | NA | Not specified | 6 |
| Adler BL et al., 2020 | Yes | Yes | Yes | Yes | Not specified | Yes | Yes | NA | NA | Not specified | 6 |
| Hong SH et al., 2017 | Yes | Yes | Yes | Yes | Not specified | Yes | Yes | NA | NA | Not specified | 6 |
| Jang SY et al., 2012 | Yes | Yes | Yes | Yes | Not specified | Yes | Yes | NA | NA | Not specified | 6 |
| Kim IY et al., 2007 | Yes | Yes | Yes | Yes | Not specified | Yes | Yes | NA | NA | Not specified | 6 |
| Kim J et al., 2006 | Yes | Yes | Yes | Yes | Not specified | Yes | Yes | NA | NA | Not specified | 6 |
| Han SR et al., 2001 | Yes | Yes | Yes | Yes | Not specified | Yes | Yes | NA | NA | Not specified | 6 |
| 김우준 et al., 2006 | Yes | Yes | Yes | Yes | Not specified | Yes | Yes | NA | NA | Not specified | 6 |
| 박상혁 et al., 2004 | Yes | Yes | Yes | Yes | Not specified | Yes | Yes | NA | NA | Not specified | 6 |
| Nkwerem S et al., 2017 | Yes | Yes | Yes | Yes | Not specified | Yes | Yes | NA | NA | Not specified | 6 |
| Shirakawa K et al., 2010 | Yes | Yes | Yes | Yes | Not specified | Yes | Yes | NA | NA | Not specified | 6 |
| Murata K et al., 2007 | Yes | Yes | Yes | Yes | Not specified | Yes | Yes | NA | NA | Not specified | 6 |
| Nobayashi M et al., 2006 | Yes | Yes | Yes | Yes | Not specified | Yes | Yes | NA | NA | Not specified | 6 |
| Rathore A et al., 2024 | Yes | Yes | Yes | Yes | Not specified | Yes | Yes | NA | NA | Not specified | 6 |
| Kaur S et al., 2023 | Yes | Yes | Yes | Yes | Not specified | Yes | Yes | NA | NA | Not specified | 6 |
| Rengarajan S et al., 2008 | Yes | Yes | Yes | Yes | Not specified | Yes | Yes | NA | NA | Not specified | 6 |
| Sundaram C et al., 2003 | Yes | Yes | Yes | Yes | Not specified | Yes | Yes | NA | NA | Not specified | 6 |
| Cummings TJ et al., 2000 | Yes | Yes | Yes | Yes | Not specified | Yes | Yes | NA | NA | Not specified | 6 |
| Park HR et al., 2025 | Yes | Yes | Yes | Yes | Not specified | Yes | Yes | NA | NA | Not specified | 6 |
| Nam Seung-Joo et al., 2018 | Yes | Yes | Yes | Yes | Not specified | Yes | Yes | NA | NA | Not specified | 6 |
| Walker P et al., 2007 | Yes | Yes | Yes | Yes | Not specified | Yes | Yes | NA | NA | Not specified | 6 |
| Jones MC et al., 2013 | Yes | Yes | Yes | Yes | Not specified | Yes | Yes | NA | NA | Not specified | 6 |
| Gonzenbach RR et al., 2013 | Yes | Yes | Yes | Yes | Not specified | Yes | Yes | NA | NA | Not specified | 6 |
| Alibhoy AT et al., 2007 | Yes | Yes | Yes | Yes | Not specified | Yes | Yes | NA | NA | Not specified | 6 |
| Lo Presti A et al., 2015 | Yes | Yes | Yes | Yes | Not specified | Yes | Yes | NA | NA | Not specified | 6 |
| Caballero J et al., 2015 | Yes | Yes | Yes | Yes | Not specified | Yes | Yes | NA | NA | Not specified | 6 |
| Jeong SC et al., 1998 | Yes | Yes | Yes | Yes | Not specified | Yes | Yes | NA | NA | Not specified | 6 |
| Kim CY et al., 1997 | Yes | Yes | Yes | Yes | Not specified | Yes | Yes | NA | NA | Not specified | 6 |
| Kim DG et al., 1996 | Yes | Yes | Yes | Yes | Not specified | Yes | Yes | NA | NA | Not specified | 6 |
| Moon WK et al., 1993 | Yes | Yes | Yes | Yes | Not specified | Yes | Yes | NA | NA | Not specified | 6 |
| Chang KH et al., 1992 | Yes | Yes | Yes | Yes | Not specified | Yes | Yes | NA | NA | Not specified | 6 |
| Chang KH et al., 1987 | Yes | Yes | Yes | Yes | Not specified | Yes | Yes | NA | NA | Not specified | 6 |
| Kim Seong Ho et al., 1988 | Yes | Yes | Yes | Yes | Not specified | Yes | Yes | NA | NA | Not specified | 6 |
| Okamura T et al., 1995 | Yes | Yes | Yes | Yes | Not specified | Yes | Yes | NA | NA | Not specified | 6 |
| Yamashita K et al., 1990 | Yes | Yes | Yes | Yes | Not specified | Yes | Yes | NA | NA | Not specified | 6 |
| Anegawa S et al., 1989 | Yes | Yes | Yes | Yes | Not specified | Yes | Yes | NA | NA | Not specified | 6 |
| Yamashita K et al., 1989 | Yes | Yes | Yes | Yes | Not specified | Yes | Yes | NA | NA | Not specified | 6 |
| Haruzono A et al., 1988 | Yes | Yes | Yes | Yes | Not specified | Yes | Yes | NA | NA | Not specified | 6 |
| Nakajima H et al., 1984 | Yes | Yes | Yes | Yes | Not specified | Yes | Yes | NA | NA | Not specified | 6 |
| Chamadol W et al., 1992 | Yes | Yes | Yes | Yes | Not specified | Yes | Yes | NA | NA | Not specified | 6 |
| Kradel J et al., 1993 | Yes | Yes | Yes | Yes | Not specified | Yes | Yes | NA | NA | Not specified | 6 |
| Mitchell A et al., 1990 | Yes | Yes | Yes | Yes | Not specified | Yes | Yes | NA | NA | Not specified | 6 |
| Anders K et al., 1984 | Yes | Yes | Yes | Yes | Not specified | Yes | Yes | NA | NA | Not specified | 6 |
| Munckhof WJ et al., 1994 | Yes | Yes | Yes | Yes | Not specified | Yes | Yes | NA | NA | Not specified | 6 |
| Holodniy M et al., 1991 | Yes | Yes | Yes | Yes | Not specified | Yes | Yes | NA | NA | Not specified | 6 |
| Mineura K et al., 1980 | Yes | Yes | Yes | Yes | Not specified | Yes | Yes | NA | NA | Not specified | 6 |
| Boero AM et al., 1991 | Yes | Yes | Yes | Yes | Not specified | Yes | Yes | NA | NA | Not specified | 6 |
| Fan KJ et al., 1986 | Yes | Yes | Yes | Yes | Not specified | Yes | Yes | NA | NA | Not specified | 6 |
| Landero A et al., 1991 | Yes | Yes | Yes | Yes | Not specified | Yes | Yes | NA | NA | Not specified | 6 |
| Oh SI et al., 2011 | Yes | Yes | Yes | Yes | Not specified | Yes | Yes | NA | NA | Not specified | 6 |
| Park JH et al., 2011 | Yes | Yes | Yes | Yes | Not specified | Yes | Yes | NA | NA | Not specified | 6 |
| Kwon JH et al., 2004 | Yes | Yes | Yes | Yes | Not specified | Yes | Yes | NA | NA | Not specified | 6 |
| Chotmongkol V et al., 2021 | Yes | Yes | Yes | Yes | Not specified | Yes | Yes | NA | NA | Not specified | 6 |
| Iampreechakul P et al., 2020 | Yes | Yes | Yes | Yes | Not specified | Yes | Yes | NA | NA | Not specified | 6 |
| Chotmongkol V et al., 2018 | Yes | Yes | Yes | Yes | Not specified | Yes | Yes | NA | NA | Not specified | 6 |
| Noiphithak R et al., 2016 | Yes | Yes | Yes | Yes | Not specified | Yes | Yes | NA | NA | Not specified | 6 |
| Boonyasiri A et al., 2013 | Yes | Yes | Yes | Yes | Not specified | Yes | Yes | NA | NA | Not specified | 6 |
| Cho YD et al., 1992 | Yes | Yes | Yes | Yes | Not specified | Yes | Yes | NA | NA | Not specified | 6 |
| Kudesia S et al., 1998 | Yes | Yes | Yes | Yes | Not specified | Yes | Yes | NA | NA | Not specified | 6 |
| Luo B et al., 2018 | Yes | Yes | Yes | Yes | Not specified | Yes | Yes | NA | NA | Not specified | 6 |
| Xu JM et al., 2021 | Yes | Yes | Yes | Yes | Not specified | Yes | Yes | NA | NA | Not specified | 6 |
| Li Z et al., 2017 | Yes | Yes | Yes | Yes | Not specified | Yes | Yes | NA | NA | Not specified | 6 |
| Luo QP et al., 2016 | Yes | Yes | Yes | Yes | Not specified | Yes | Yes | NA | NA | Not specified | 6 |
| Wang G et al., 2014 | Yes | Yes | Yes | Yes | Not specified | Yes | Yes | NA | NA | Not specified | 6 |
| Zheng L et al., 2014 | Yes | Yes | Yes | Yes | Not specified | Yes | Yes | NA | NA | Not specified | 6 |
| Jin Y et al., 2010 | Yes | Yes | Yes | Yes | Not specified | Yes | Yes | NA | NA | Not specified | 6 |
| Niu HL et al., 2010 | Yes | Yes | Yes | Yes | Not specified | Yes | Yes | NA | NA | Not specified | 6 |
| Huang AM et al., 2009 | Yes | Yes | Yes | Yes | Not specified | Yes | Yes | NA | NA | Not specified | 6 |
| Wang XD, 2004 | Yes | Yes | Yes | Yes | Not specified | Yes | Yes | NA | NA | Not specified | 6 |
| Li CP et al., 2003 | Yes | Yes | Yes | Yes | Not specified | Yes | Yes | NA | NA | Not specified | 6 |
| Liu HC et al., 2002 | Yes | Yes | Yes | Yes | Not specified | Yes | Yes | NA | NA | Not specified | 6 |
| Yang GF et al., 2002 | Yes | Yes | Yes | Yes | Not specified | Yes | Yes | NA | NA | Not specified | 6 |
| Wu KS, 1999 | Yes | Yes | Yes | Yes | Not specified | Yes | Yes | NA | NA | Not specified | 6 |
| Jin CH, 1998 | Yes | Yes | Yes | Yes | Not specified | Yes | Yes | NA | NA | Not specified | 6 |
| Zhu Y et al., 1994 | Yes | Yes | Yes | Yes | Not specified | Yes | Yes | NA | NA | Not specified | 6 |
| Xia SQ et al., 1994 | Yes | Yes | Yes | Yes | Not specified | Yes | Yes | NA | NA | Not specified | 6 |
| Ning JP et al., 1986 | Yes | Yes | Yes | Yes | Not specified | Yes | Yes | NA | NA | Not specified | 6 |
| Zan YJ, 1990 | Yes | Yes | Yes | Yes | Not specified | Yes | Yes | NA | NA | Not specified | 6 |
| Yu ZF et al., 1980 | Yes | Yes | Yes | Yes | Not specified | Yes | Yes | NA | NA | Not specified | 6 |
| Yi W, 1980 | Yes | Yes | Yes | Yes | Not specified | Yes | Yes | NA | NA | Not specified | 6 |
| Fan PF et al., 1976 | Yes | Yes | Yes | Yes | Not specified | Yes | Yes | NA | NA | Not specified | 6 |
| Wang GL et al., 2007 | Yes | Yes | Yes | Yes | Not specified | Yes | Yes | NA | NA | Not specified | 6 |
| Chen X et al., 1995 | Yes | Yes | Yes | Yes | Not specified | Yes | Yes | NA | NA | Not specified | 6 |
| Jin HY, 2006 | Yes | Yes | Yes | No | Not specified | Yes | Yes | NA | NA | Not specified | 5 |
| Park WH et al., 2014 | Yes | Yes | Yes | Yes | Not specified | Yes | Yes | NA | NA | Not specified | 6 |
| Yun SJ et al., 2010 | Yes | Yes | Yes | Yes | Not specified | Yes | Yes | NA | NA | Not specified | 6 |
| Kim SH et al., 2008 | Yes | Yes | Yes | Yes | Not specified | Yes | Yes | NA | NA | Not specified | 6 |
| Lim Dh et al., 2007 | Yes | Yes | Yes | Yes | Not specified | Yes | Yes | NA | NA | Not specified | 6 |
| Kim YJ et al., 2007 | Yes | Yes | Yes | Yes | Not specified | Yes | Yes | NA | NA | Not specified | 6 |
| Jeong HJ et al., 2004 | Yes | Yes | Yes | Yes | Not specified | Yes | Yes | NA | NA | Not specified | 6 |
| Eun Tak Kim et al., 2012 | Yes | Yes | Yes | Yes | Not specified | Yes | Yes | NA | NA | Not specified | 6 |
| Lee Hae-Hyeog et al., 2010 | Yes | Yes | Yes | Yes | Not specified | Yes | Yes | NA | NA | Not specified | 6 |
| Cha Sang-Hoon et al., 2004 | Yes | Yes | Yes | Yes | Not specified | Yes | Yes | NA | NA | Not specified | 6 |
| Trupti B et al., 2018 | Yes | Yes | Yes | Yes | Not specified | Yes | Yes | NA | NA | Not specified | 6 |
| Sakamoto T et al., 2003 | Yes | Yes | Yes | Yes | Not specified | Yes | Yes | NA | NA | Not specified | 6 |
| Oh SJ et al., 1993 | Yes | Yes | Yes | Yes | Not specified | Yes | Yes | NA | NA | Not specified | 6 |
| Seo BS et al., 1964 | Yes | Yes | Yes | Yes | Not specified | Yes | Yes | NA | NA | Not specified | 6 |
| Fang LJ et al.,2020 | Yes | Yes | Yes | Yes | Not specified | Yes | Yes | NA | NA | Not specified | 6 |
| Chen XY et al., 2015 | Yes | Yes | Yes | Yes | Not specified | Yes | Yes | NA | NA | Not specified | 6 |
| Xu P et al., 2009 | Yes | Yes | Yes | Yes | Not specified | Yes | Yes | NA | NA | Not specified | 6 |
| Gan JH et al.,2002 | Yes | Yes | Yes | Yes | Not specified | Yes | Yes | NA | NA | Not specified | 6 |
| Liu HX et al., 1959 | Yes | Yes | Yes | Yes | Not specified | Yes | Yes | NA | NA | Not specified | 6 |
| Yang E et al., 2022 | Yes | Yes | Yes | Yes | Not specified | Yes | Yes | NA | NA | Not specified | 6 |
| Ishida Y et al., 1996 | Yes | Yes | Yes | Yes | Not specified | Yes | Yes | NA | NA | Not specified | 6 |
| Khamboonruang C et al., 1974 | Yes | Yes | Yes | Yes | Not specified | Yes | Yes | NA | NA | Not specified | 6 |
| He M,2012 | Yes | Yes | Yes | No | Not specified | Yes | Yes | NA | NA | Not specified | 5 |
| Shen MQ et al.,2011 | Yes | Yes | Yes | Yes | Not specified | Yes | Yes | NA | NA | Not specified | 6 |
| He M et al., 2010 | Yes | Yes | Yes | Yes | Not specified | Yes | Yes | NA | NA | Not specified | 6 |
| Zhou JJ, 2011 | Yes | Yes | Yes | Yes | Not specified | Yes | Yes | NA | NA | Not specified | 6 |
| Wen Y et al.,1996 | Yes | Yes | Yes | Yes | Not specified | Yes | Yes | NA | NA | Not specified | 6 |
| Chen SL et al.,1995 | Yes | Yes | Yes | Yes | Not specified | Yes | Yes | NA | NA | Not specified | 6 |
| Shen SM,1994 | Yes | Yes | Yes | Yes | Not specified | Yes | Yes | NA | NA | Not specified | 6 |
| Zhang YM et al.,1991 | Yes | Yes | Yes | Yes | Not specified | Yes | Yes | NA | NA | Not specified | 6 |
| Yang CY,1990 | Yes | Yes | Yes | Yes | Not specified | Yes | Yes | NA | NA | Not specified | 6 |
| Chen XZ et al.,1987 | Yes | Yes | Yes | Yes | Not specified | Yes | Yes | NA | NA | Not specified | 6 |
| Song WJ et al.,2022 | Yes | Yes | Yes | Yes | Not specified | Yes | Yes | NA | NA | Not specified | 6 |
| Zhang KR et al.,2021 | Yes | Yes | Yes | Yes | Not specified | Yes | Yes | NA | NA | Not specified | 6 |
| Shi DM et al.,2020 | Yes | Yes | Yes | Yes | Not specified | Yes | Yes | NA | NA | Not specified | 6 |
| Fang ZM et al.,2020 | Yes | Yes | Yes | Yes | Not specified | Yes | Yes | NA | NA | Not specified | 6 |
| Luo LJ et al., 2019 | Yes | Yes | Yes | Yes | Not specified | Yes | Yes | NA | NA | Not specified | 6 |
| Song Y et al., 2019 | Yes | Yes | Yes | Yes | Not specified | Yes | Yes | NA | NA | Not specified | 6 |
| He Li et al.,2019 | Yes | Yes | Yes | No | Not specified | Yes | Yes | NA | NA | Not specified | 5 |
| He HP et al.,2019 | Yes | Yes | Yes | Yes | Not specified | Yes | Yes | NA | NA | Not specified | 6 |
| Xu Y et al.,2017 | Yes | Yes | Yes | Yes | Not specified | Yes | Yes | NA | NA | Not specified | 6 |
| Tommy H.C. et al., 2017 | Yes | Yes | Yes | Yes | Not specified | Yes | Yes | NA | NA | Not specified | 6 |
| Zhang HF et al., 2017 | Yes | Yes | Yes | Yes | Not specified | Yes | Yes | NA | NA | Not specified | 6 |
| Wang HB et al., 2015 | Yes | Yes | Yes | No | Not specified | Yes | Yes | NA | NA | Not specified | 5 |
| Mo ZS et al., 2013 | Yes | Yes | Yes | Yes | Not specified | Yes | Yes | NA | NA | Not specified | 6 |
| Zeng QR et al.,2012 | Yes | Yes | Yes | Yes | Not specified | Yes | Yes | NA | NA | Not specified | 6 |
| Qin YH et al.,2012 | Yes | Yes | Yes | Yes | Not specified | Yes | Yes | NA | NA | Not specified | 6 |
| Bai J et al., 2012 | Yes | Yes | Yes | Yes | Not specified | Yes | Yes | NA | NA | Not specified | 6 |
| Li RY et al.,2011 | Yes | Yes | Yes | Yes | Not specified | Yes | Yes | NA | NA | Not specified | 6 |
| Lin XM et al., 2009 | Yes | Yes | Yes | Yes | Not specified | Yes | Yes | NA | NA | Not specified | 6 |
| Xu YM et al.,2008 | Yes | Yes | Yes | Yes | Not specified | Yes | Yes | NA | NA | Not specified | 6 |
| Zheng SZ et al.,2005 | Yes | Yes | Yes | Yes | Not specified | Yes | Yes | NA | NA | Not specified | 6 |
| Lin BL et al.,2003 | Yes | Yes | Yes | Yes | Not specified | Yes | Yes | NA | NA | Not specified | 6 |
| Liu JC et al.,2003 | Yes | Yes | Yes | Yes | Not specified | Yes | Yes | NA | NA | Not specified | 6 |
| Huang JM,2003 | Yes | Yes | Yes | Yes | Not specified | Yes | Yes | NA | NA | Not specified | 6 |
| Fan SQ et al.,2001 | Yes | Yes | Yes | Yes | Not specified | Yes | Yes | NA | NA | Not specified | 6 |
| Long YG et al.,1998 | Yes | Yes | Yes | Yes | Not specified | Yes | Yes | NA | NA | Not specified | 6 |
| Pan CW,1993 | Yes | Yes | Yes | Yes | Not specified | Yes | Yes | NA | NA | Not specified | 6 |
| Wang L et al.,1992 | Yes | Yes | Yes | Yes | Not specified | Yes | Yes | NA | NA | Not specified | 6 |
| Hong JH et al.,1990 | Yes | Yes | Yes | Yes | Not specified | Yes | Yes | NA | NA | Not specified | 6 |
| Ng T H et al.,1989 | Yes | Yes | Yes | Yes | Not specified | Yes | Yes | NA | NA | Not specified | 6 |
| Li SR,1988 | Yes | Yes | Yes | Yes | Not specified | Yes | Yes | NA | NA | Not specified | 6 |
| Chinese Journal of Parasitology and Parasitic Diseases,1988(01):77. | Yes | Yes | Yes | Yes | Not specified | Yes | Yes | NA | NA | Not specified | 6 |
| Liu JC et al.,1988 | Yes | Yes | Yes | Yes | Not specified | Yes | Yes | NA | NA | Not specified | 6 |
| Chinese Journal of Parasitology and Parasitic Diseases,1987(01):78. | Yes | Yes | Yes | Yes | Not specified | Yes | Yes | NA | NA | Not specified | 6 |
| Pu DM et al.,1987 | Yes | Yes | Yes | Yes | Not specified | Yes | Yes | NA | NA | Not specified | 6 |
| Feng NS,1986 | Yes | Yes | Yes | Yes | Not specified | Yes | Yes | NA | NA | Not specified | 6 |
| Zhang QQ,1984 | Yes | Yes | Yes | Yes | Not specified | Yes | Yes | NA | NA | Not specified | 6 |
| Li GL et al.,1982 | Yes | Yes | Yes | No | Not specified | Yes | Yes | NA | NA | Not specified | 5 |
| Li R et al.,1981 | Yes | Yes | Yes | Yes | Not specified | Yes | Yes | NA | NA | Not specified | 6 |
| Wu DM et al.,1980 | Yes | Yes | Yes | No | Not specified | Yes | Yes | NA | NA | Not specified | 5 |
| Yang ZX et al.,1979 | Yes | Yes | Yes | Yes | Not specified | Yes | Yes | NA | NA | Not specified | 6 |
| Lu CL et al.,1976 | Yes | Yes | Yes | Yes | Not specified | Yes | Yes | NA | NA | Not specified | 6 |
| Cai HQ et al.,1983 | Yes | Yes | Yes | Yes | Not specified | Yes | Yes | NA | NA | Not specified | 6 |
| Kim JG et al., 2018 | Yes | Yes | Yes | Yes | Not specified | Yes | Yes | NA | NA | Not specified | 6 |
| Hong SJ et al., 2010 | Yes | Yes | Yes | Yes | Not specified | Yes | Yes | NA | NA | Not specified | 6 |
| Choi WH et al., 2010 | Yes | Yes | Yes | No | Not specified | Yes | Yes | NA | NA | Not specified | 5 |
| Cho JH et al., 2000 | Yes | Yes | Yes | Yes | Not specified | Yes | Yes | NA | NA | Not specified | 6 |
| Chi JG et al., 1988 | Yes | Yes | Yes | No | Not specified | Yes | Yes | NA | NA | Not specified | 5 |
| Cho SY et al., 1975 | Yes | Yes | Yes | Yes | Not specified | Yes | Yes | NA | NA | Not specified | 6 |
| Boonyasiri A et al., 2014 | Yes | Yes | Yes | Yes | Not specified | Yes | Yes | NA | NA | Not specified | 6 |
| Koonmee S et al., 2011 | Yes | Yes | Yes | Yes | Not specified | Yes | Yes | NA | NA | Not specified | 6 |
| Anantaphruti MT et al., 2011 | Yes | Yes | Yes | Yes | Not specified | Yes | Yes | NA | NA | Not specified | 6 |
| Wiwanitkit V et al., 2005 | Yes | Yes | Yes | Yes | Not specified | Yes | Yes | NA | NA | Not specified | 6 |
| Norman SH et al., 1980 | Yes | Yes | Yes | Yes | Not specified | Yes | Yes | NA | NA | Not specified | 6 |
| Nawa Y et al., 2024 | Yes | Yes | Yes | Yes | Not specified | Yes | Yes | NA | NA | Not specified | 6 |
| Kikuchi T et al., 2020 | Yes | Yes | Yes | Yes | Not specified | Yes | Yes | NA | NA | Not specified | 6 |
| Tran QR et al., 2019 | Yes | Yes | Yes | Yes | Not specified | Yes | Yes | NA | NA | Not specified | 6 |
| Eberhard ML et al., 2015 | Yes | Yes | Yes | Yes | Not specified | Yes | Yes | NA | NA | Not specified | 6 |
| Cao Q et al.,2016 | Yes | Yes | Yes | No | Not specified | Yes | Yes | NA | NA | Not specified | 5 |
| Cao S et al.,2013 | Yes | Yes | Yes | Yes | Not specified | Yes | Yes | NA | NA | Not specified | 6 |
| Chen J, 2012 | Yes | Yes | Yes | Yes | Not specified | Yes | Yes | NA | NA | Not specified | 6 |
| Chen HN et al.,2009 | Yes | Yes | Yes | No | Not specified | Yes | Yes | NA | NA | Not specified | 5 |
| Chen SH et al.,2014 | Yes | Yes | Yes | No | Not specified | Yes | Yes | NA | NA | Not specified | 5 |
| Zhang RL et al.,2003 | Yes | Yes | Yes | No | Not specified | Yes | Yes | NA | NA | Not specified | 5 |
| Lu L et al.,2019 | Yes | Yes | Yes | Yes | Not specified | Yes | Yes | NA | NA | Not specified | 6 |
| Wang Z Q et al., 2014 | Yes | Yes | Yes | Yes | Not specified | Yes | Yes | NA | NA | Not specified | 6 |
| Li XM et al.,1994 | Yes | Yes | Yes | Yes | Not specified | Yes | Yes | NA | NA | Not specified | 6 |
| Lee KJ et al., 2002 | Yes | Yes | Yes | Yes | Not specified | Yes | Yes | NA | NA | Not specified | 6 |
| Moon JR et al., 1976 | Yes | Yes | Yes | No | Not specified | Yes | Yes | NA | NA | Not specified | 5 |
| SWARTZWELDER JC et al., 1964 | Yes | Yes | Yes | No | Not specified | Yes | Yes | NA | NA | Not specified | 5 |
| OSIMANI JJ et al., 1954 | Yes | Yes | Yes | No | Not specified | Yes | Yes | NA | NA | Not specified | 5 |
| COX PS et al., 1965 | Yes | Yes | Yes | No | Not specified | Yes | Yes | NA | NA | Not specified | 5 |
| Rolón PA et al., 1976 | Yes | Yes | Yes | No | Not specified | Yes | Yes | NA | NA | Not specified | 5 |
| GARCIA OCAMPO N et al., 1961 | Yes | Yes | Yes | No | Not specified | Yes | Yes | NA | NA | Not specified | 5 |
| Ordoñez-Chinguel, A et al. 2025 | Yes | Yes | Yes | Yes | Not specified | Yes | Yes | NA | NA | Not specified | 6 |
| Hawkins et al., 2024 | Yes | Yes | Yes | Yes | Not specified | Yes | Yes | NA | NA | Not specified | 6 |
| Feigenbaum L et al., 2016 | Yes | Yes | Yes | No | Not specified | Yes | Yes | NA | NA | Not specified | 5 |
| Jin et al., 2014 | Yes | Yes | Yes | No | Not specified | Yes | Yes | NA | NA | Not specified | 5 |
| Nakasone N et al., 2012 | Yes | Yes | Yes | Yes | Not specified | Yes | Yes | NA | NA | Not specified | 6 |
| Yamanaka T et al., 2010 | Yes | Yes | Yes | Yes | Not specified | Yes | Yes | NA | NA | Not specified | 6 |
| Gotoh K et al., 2007 | Yes | Yes | Yes | Yes | Not specified | Yes | Yes | NA | NA | Not specified | 6 |
| Yang KM et al., 2005 | Yes | Yes | Yes | No | Not specified | Yes | Yes | NA | NA | Not specified | 5 |
| Lee HW et al., 2005 | Yes | Yes | Yes | No | Not specified | Yes | Yes | NA | NA | Not specified | 5 |
| KittiponghansaS et al., 1988 | Yes | Yes | Yes | No | Not specified | Yes | Yes | NA | NA | Not specified | 5 |
| Oda T et al., 1982 | Yes | Yes | Yes | Yes | Not specified | Yes | Yes | NA | NA | Not specified | 6 |
| Ru SS et al., 2024 | Yes | Yes | Yes | Yes | Not specified | Yes | Yes | NA | NA | Not specified | 6 |
| Mao C et al., 2021 | Yes | Yes | Yes | Yes | Not specified | Yes | Yes | NA | NA | Not specified | 6 |
| Nomura Y et al., 2020 | Yes | Yes | Yes | Yes | Not specified | Yes | Yes | NA | NA | Not specified | 6 |
| Schauer F et al., 2013 | Yes | Yes | Yes | Yes | Not specified | Yes | Yes | NA | NA | Not specified | 6 |
| Hsu T H et al., 2020 | Yes | Yes | Yes | Yes | Not specified | Yes | Yes | NA | NA | Not specified | 6 |
| Kim H J et al., 2012 | Yes | Yes | Yes | Yes | Not specified | Yes | Yes | NA | NA | Not specified | 6 |
| Li HX et al., 2017 | Yes | Yes | Yes | Yes | Not specified | Yes | Yes | NA | NA | Not specified | 6 |
| Sonosaki T et al., 2016 | Yes | Yes | Yes | No | Not specified | Yes | Yes | NA | NA | Not specified | 5 |
| Horiguchi Y et al., 2013 | Yes | Yes | Yes | Yes | Not specified | Yes | Yes | NA | NA | Not specified | 6 |
| Wang LT et al., 1974 | Yes | Yes | Yes | Yes | Not specified | Yes | Yes | NA | NA | Not specified | 6 |
| Yamanaka S et al., 2007 | Yes | Yes | Yes | Yes | Not specified | Yes | Yes | NA | NA | Not specified | 6 |
| Shin D J, 2023 | Yes | Yes | Yes | Yes | Not specified | Yes | Yes | NA | NA | Not specified | 6 |
| Aoshima M et al., 1989 | Yes | Yes | Yes | Yes | Not specified | Yes | Yes | NA | NA | Not specified | 6 |
| McQuay R M et al., 1966 | Yes | Yes | Yes | No | Not specified | Yes | Yes | NA | NA | Not specified | 5 |
| Choi MinHo C M H et al., 2012 | Yes | Yes | Yes | Yes | Not specified | Yes | Yes | NA | NA | Not specified | 6 |
| Albán M F et al., 2009 | Yes | Yes | Yes | No | Not specified | Yes | Yes | NA | NA | Not specified | 5 |
| Fukatsu T et al., 2000 | Yes | Yes | Yes | Yes | Not specified | Yes | Yes | NA | NA | Not specified | 6 |
| Zhang YL et al., 2024 | Yes | Yes | Yes | No | Not specified | Yes | Yes | NA | NA | Not specified | 5 |
| Zhang ZN et al., 2021 | Yes | Yes | Yes | No | Not specified | Yes | Yes | NA | NA | Not specified | 5 |
| Zhang YL et al., 2020 | Yes | Yes | Yes | No | Not specified | Yes | Yes | NA | NA | Not specified | 5 |
| Song P et al., 2018 | Yes | Yes | Yes | No | Not specified | Yes | Yes | NA | NA | Not specified | 5 |
| Jiang DX et al., 2017 | Yes | Yes | Yes | No | Not specified | Yes | Yes | NA | NA | Not specified | 5 |
| Chen SH et al., 2014 | Yes | Yes | Yes | No | Not specified | Yes | Yes | NA | NA | Not specified | 5 |
| Wang J et al., 2010 | Yes | Yes | Yes | No | Not specified | Yes | Yes | NA | NA | Not specified | 5 |
| Huang DN et al., 2015 | Yes | Yes | Yes | No | Not specified | Yes | Yes | NA | NA | Not specified | 5 |
| Wang ZQ et al., 2014 | Yes | Yes | Yes | Yes | Not specified | Yes | Yes | NA | NA | Not specified | 6 |
| Lee MK et al., 2010 | Yes | Yes | Yes | No | Not specified | Yes | Yes | NA | NA | Not specified | 5 |
| Lee MR et al., 2017 | Yes | Yes | Yes | No | Not specified | Yes | Yes | NA | NA | Not specified | 5 |
| Park HY et al., 2001 | Yes | Yes | Yes | No | Not specified | Yes | Yes | NA | NA | Not specified | 5 |
| Kong Y et al., 1994 | Yes | Yes | Yes | Yes | Not specified | Yes | Yes | NA | NA | Not specified | 6 |
| Kavana N et al., 2016 | Yes | Yes | Yes | Yes | Not specified | Yes | Yes | NA | NA | Not specified | 6 |
| Lee KJ et al., 2002 | Yes | Yes | Yes | Yes | Not specified | Yes | Yes | NA | NA | Not specified | 6 |
| Jin Y et al., 2017 | Yes | Yes | Yes | No | Not specified | Yes | Yes | NA | NA | Not specified | 5 |

**Reference**

1. Munn Z, Moola S, Riitano D, Lisy K. The development of a critical appraisal tool for use in systematic reviews addressing questions of prevalence. Int J Health Policy Manag. 2014;3(3):123-8.
