# Supplementary material for: Current status, challenges and control of human sparganosis worldwide: a scoping review
Source: Infect Dis Poverty. 2026 Mar 31;15:38. doi: 10.1186/s40249-026-01434-9 (PMC13037078; doi:10.1186/s40249-026-01434-9)
Supplement: Supplementary file 3 — Additional file 3. [file 40249_2026_1434_MOESM3_ESM.docx]

**Table** Misdiagnosed cases of CNS sparganosis

| Author(s) and Year | Number of misdiagnosed cases |
| --- | --- |
| Rong ZL et al.,2022 | 1 |
| Pan LY et al.,2021 | 1 |
| Wu L et al., 2017 | 2 |
| Ma Y et al.,2018 | 1 |
| Li DT et al.,2012 | 4 |
| Wang P et al.,2012 | 1 |
| Wang Y et al.,2009 | 3 |
| Jiang BD et al.,2009 | 4 |
| Lei ZY et al.,2010 | 1 |
| Li HF et al., 2008 | 1 |
| Zhou JG et al.,2008 | 1 |
| Liang XQ et al., 2008 | 1 |
| Gong YX et al.,2007 | 1 |
| Gao B et al., 2006. | 1 |
| Gao LM et al.,2005 | 1 |
| Lu YH et al.,2003 | 1 |
| Ke ZB et al.,2004 | 1 |
| Huang CH et al.,2003 | 1 |
| Fang ZM et al.,2003 | 1 |
| Zhao XL et al.,2002 | 1 |
| Jin XX et al.,2002 | 1 |
| Zhao YG et al.,1999 | 1 |
| Liang C et al.,1999 | 1 |
| Deng YF et al.,1995 | 1 |
| Hu WA et al.,1994 | 1 |
| Pan L et al., 1994 | 1 |
| Liu ZH et al.,1990 | 1 |
| Chan S T et al.,1987 | 2 |
| Niu Q et al.,1995 | 2 |
| Ru JS et al.,1985 | 1 |
| Jiang JN et al.,1987 | 1 |
| Zhang FL et al.,1983 | 1 |
| Ding YJ et al.,2017 | 8 |
| Liu RW et al., 2014 | 7 |
| Gong CG et al.,2008 | 4 |
| Guo EP et al.,2004 | 2 |
| Xie ZM et al., 2008 | 2 |
| Chen H et al.,1999 | 6 |
| Wang HD et al.,1996 | 1 |
| Yusi Chen et al.,2022 | 1 |
| Tsai MD et al., 1993 | 1 |
| Qianqian Zhao et al.,2023 | 1 |
| Guo J et al.,2007 | 1 |
| Zhao HM et al.,1998 | 1 |
| Li XR,2001 | 1 |
| Zhang XZ et al.,1987 | 2 |
| Yang JH et al.,2021 | 1 |
| Jun Liu et al.,2022 | 1 |
| Yiming Meng et al.,2023 | 1 |
| JianFeng Fan et al.,2021 | 1 |
| Xing WR et al.,2021 | 1 |
| Shi DM et al.,2020 | 13 |
| Fang ZM et al.,2020 | 1 |
| Liu JC et al.,2003 | 1 |
| Wang L et al.,1992 | 1 |
| Jeong SC et al., 1998 | 1 |
| 김우준 et al.,2006 | 1 |
| Nkwerem S et al.,2017 | 1 |
| Murata K et al., 2007 | 1 |
| Nobayashi M et al., 2006 | 1 |
| Okamura T et al., 1995 | 1 |
| Yamashita K et al., 1990 | 2 |
| Anegawa S et al., 1989 | 1 |
| Yamashita K et al., 1989 | 1 |
| Haruzono A et al., 1988 | 1 |
| Nakajima H et al., 1984 | 1 |
| Rathore A et al., 2024 | 1 |
| Kaur S et al., 2023 | 1 |
| Rengarajan S et al., 2008 | 1 |
| Sundaram C et al., 2003 | 1 |
| Mitchell A et al., 1990 | 1 |
| Anders K et al., 1984 | 1 |
| Park HR et al., 2025 | 1 |
| Bennett HM et al., 2014 | 1 |
| Walker P et al., 2007 | 1 |
| Munckhof WJ et al., 1994 | 1 |
| Jones MC et al.,2013 | 1 |
| Boero AM et al., 1991 | 1 |
| Lo Presti A et al., 2015 | 1 |
| Landero A et al., 1991 | 1 |
| Huang WB et al., 2020 | 1 |
| Wang SC，1995 | 1 |
| Chen H et al.,2003 | 10 |
| Bao XY et al.,2008 | 1 |
| Liu YJ, 2006 | 1 |
| Mao C et al.,2021 | 1 |
